# Dissemination of public health research to prevent non-communicable diseases: a scoping review

**DOI:** 10.1186/s12889-023-15622-x

**Published:** 2023-04-24

**Authors:** Heidi Turon, Luke Wolfenden, Meghan Finch, Sam McCrabb, Shaan Naughton, Sean R O’Connor, Ana Renda, Emily Webb, Emma Doherty, Eloise Howse, Cheryce L Harrison, Penelope Love, Natasha Smith, Rachel Sutherland, Sze Lin Yoong

**Affiliations:** 1 School of Medicine and Public Health, College of Health, Medicine and Wellbeing, The University of Newcastle, Callaghan, NSW Australia; 2 Hunter Medical Research Institute, New Lambton Heights, NSW 2305 Australia; 3 Population Health, Hunter New England Local Health District, Wallsend, NSW 2287 Australia; 4 Global Centre for Preventive Health and Nutrition, Institute for Health Transformation, School of Health and Social Development, Faculty of Health, Deakin University, Geelong, 3220 Australia; 5 School of Psychology, Queen’s University Belfast, Malone Road, Belfast, BT9 5BN Northern Ireland; 6 Population Health, Sydney Local Health District, Camperdown, NSW 2050 Australia; 7 The Australian Prevention Partnership Centre, Sax Institute, Glebe, NSW 2037 Australia; 8 Monash Centre for Health Research and Implementation, School of Public Health and Preventive Medicine, Monash University, Clayton, VIC 3168 Australia; 9 Faculty of Health, School of Exercise and Nutrition Sciences (SENS), Institute for Physical Activity and Nutrition (IPAN), Deakin University, Geelong, VIC Australia; 10 School of Health Sciences, Swinburne University of Technology, Hawthorn, VIC 3122 Australia

**Keywords:** Dissemination, Public health, Non-communicable disease, Prevention, Scoping review

## Abstract

**Background:**

Dissemination is a critical element of the knowledge translation pathway, and a necessary step to ensure research evidence is adopted and implemented by key end users in order to improve health outcomes. However, evidence-based guidance to inform dissemination activities in research is limited. This scoping review aimed to identify and describe the scientific literature examining strategies to disseminate public health evidence related to the prevention of non-communicable diseases.

**Methods:**

Medline, PsycInfo and EBSCO Search Ultimate were searched in May 2021 for studies published between January 2000 and the search date that reported on the dissemination of evidence to end users of public health evidence, within the context of the prevention of non-communicable diseases. Studies were synthesised according to the four components of Brownson and colleagues’ Model for Dissemination of Research (source, message, channel and audience), as well as by study design.

**Results:**

Of the 107 included studies, only 14% (n = 15) directly tested dissemination strategies using experimental designs. The remainder primarily reported on dissemination preferences of different populations, or outcomes such as awareness, knowledge and intentions to adopt following evidence dissemination. Evidence related to diet, physical activity and/or obesity prevention was the most disseminated topic. Researchers were the source of disseminated evidence in over half the studies, and study findings/knowledge summaries were more frequently disseminated as the message compared to guidelines or an evidence-based program/intervention. A broad range of dissemination channels were utilised, although peer-reviewed publications/conferences and presentations/workshops predominated. Practitioners were the most commonly reported target audience.

**Conclusions:**

There is a significant gap in the peer reviewed literature, with few experimental studies published that analyse and evaluate the effect of different sources, messages and target audiences on the determinants of uptake of public health evidence for prevention. Such studies are important as they can help inform and improve the effectiveness of current and future dissemination practices in public health contexts.

**Supplementary Information:**

The online version contains supplementary material available at 10.1186/s12889-023-15622-x.

## Background

Governments and non-government funders have invested substantially in a range of effective interventions to improve public health, demonstrated by significant improvements in preventive health behaviours when tested in empirical trials [1–3]. However, knowledge produced in the course of public health research frequently fails to be adopted into routine practice, or takes an unacceptably long period of time to do so, with estimates of a gap up to 17 years [4]. Knowledge translation (KT) covers a continuum of activities that span knowledge synthesis, dissemination, exchange and application of knowledge, in this context to improve health [5]. The activity of dissemination is defined as an “active approach of spreading evidence-based interventions or knowledge to the target audience via determined channels using planned strategies” [6]. Dissemination is primarily aimed at increasing end users’ awareness and knowledge of evidence, influencing intentions to use evidence, and increasing the likelihood of evidence adoption. Dissemination science therefore is defined as a systematic approach to determining effective strategies to communicate evidence with target audiences, for the purpose of changing these dissemination outcomes [7].

**Fig. 1:**
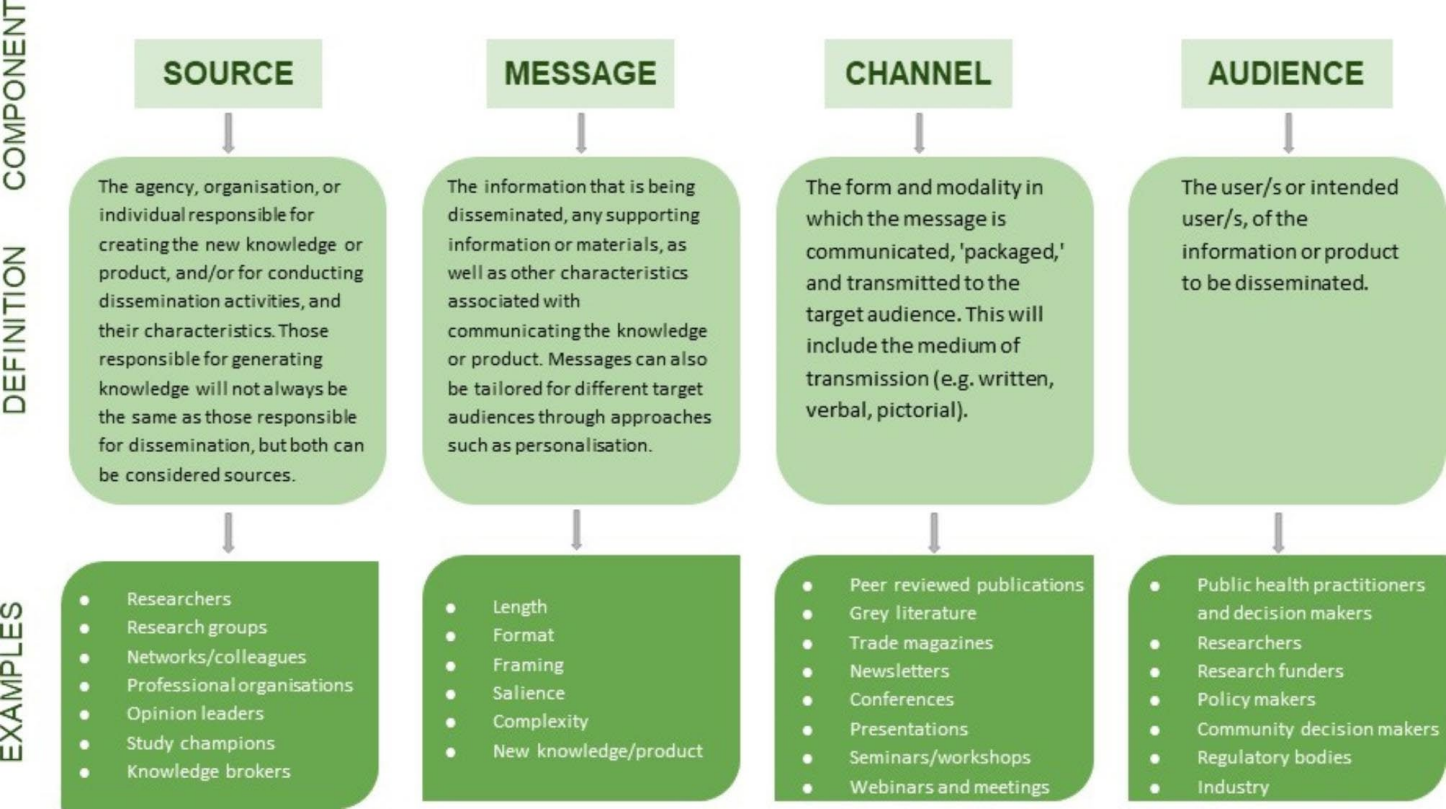
Brownson and colleagues’ Model for Dissemination of Research with examples

A number of reviews have described and synthesised various dissemination theories, models and frameworks [8, 9] that can be used to better support the dissemination of evidence to public health policy makers and practitioners. One model used in the field of public health and policy decision making is Brownson and colleagues’ Model for Dissemination of Research [10]. It is based on multiple theories including communication theory [11], and diffusion of innovations theory [12]. The framework describes four key factors that may influence the impact of a dissemination strategy [10, 13]; namely, the source (who is disseminating the information), the message (the information being communicated), the channel (how the information is communicated, e.g., modality), and the audience (the intended users of the information) (see Fig. 1) (adapted from Wilson et al. [8]):

Strategies to disseminate evidence will vary depending on the target end user and the way they use research evidence. There are a variety of end users of research evidence. For example, the dissemination of new school-based program to prevent adolescent uptake of e-cigarettes may be primarily targeted at policymakers in education and school principals, however additional tailored strategies will be important to communicate the information to other potential end users such as adolescents, parents, and school teachers. Within a field as diverse as public health, potential end users could include, but are not limited to, the community, practitioners, researchers, funders, industry bodies, and policymakers. Policymakers and practitioners are frequently the target audiences of dissemination activities as they are usually responsible for setting public health priorities, and financing and supporting the provision of public health services, as well implementing the policies. Policymakers and practitioners value evidence [14], and consider it in their decision making [15]. However, they also commonly report issues with timely access to evidence that is both relevant and useful to help inform decision making [16, 17]. Therefore, there is increasing recognition in the scientific community that dissemination efforts must go beyond presenting research findings using traditional academic methods (such as peer-reviewed journals) to ensure they are tailored and presented to the needs of different end users.[18, 19].

The field of dissemination science is a relatively new field of study and this is reflected in the debate in the literature regarding key terminology [20, 21], the importance, consistency and validity of outcomes used in research studies [22, 23] and the importance of better co-ordination of dissemination research to collectively progress the field [7]. In the field of public health specifically, dissemination, scale up and implementation strategies are often conflated or not distinguished from knowledge translation more broadly [24, 25], making it difficult to draw conclusions about the effects of specific dissemination strategies. It is also important to distinguish dissemination from the broader health communication and scale up literature. While the fields have some commonalities, health communication has been defined by the Centers for Disease Control and Prevention as “the study and use of communication strategies to inform and influence individual decisions that enhance health*”* [26] which reflects that it more commonly targets the general public as the audience in an effort to motivate behaviour change for the purpose of improving health [27–29]. This communication is distinct from that targeting decision makers and practitioners who are responsible for supporting others to use and apply evidence (e.g., through their actions in implementing evidence guidelines and programs, such as a cancer screening service, or physical activity guidelines in schools). It is this latter communication that we are classifying as dissemination.

While recognising the very broad scope of these constructs of public health, end users and dissemination, this scoping review will focus on the dissemination of evidence related to the prevention of non-communicable diseases (NCDs). NCDs are typically chronic conditions arising from genetic, behavioural or environmental factors, as opposed to infectious factors, and include conditions such as cardiovascular disease, many forms of cancer and diabetes. They impose a substantial and growing burden of disease on the global population [30], and are responsible for 74% of deaths globally each year [31]. A reduction in premature mortality due to NCDs has been identified as one of the 2030 Sustainable Development Goals by the United Nations [32] highlighting the importance of this issue. Further, consistent with our focus on dissemination, as opposed to health communication, this review will consider audiences responsible for adoption of this evidence at a community or population level. This will include policymakers, practitioners, researchers, public health administrators and other decision-makers, but will exclude the general public.

Aim.

In order for the field of dissemination science to progress and to support policy makers, practitioners and other end-users to adopt evidence in a more timely manner, it is essential that current evidence regarding dissemination strategies is mapped to determine the focus of future empirical research. As such, the primary aim of this scoping review is to identify and describe the literature examining strategies to disseminate public health evidence related to the prevention of NCDs. Secondary to this, we aimed to map studies against the components of Brownson et al’s Model for Dissemination of Research [10] and according to their research design and methods (i.e., qualitative, quantitative, interventions) in order to provide insight into the levels of evidence available for different dissemination components.

## Methods

### Protocol and registration

The methods of this scoping review were conducted in accordance with the guidance issued by JBI [33]. The findings are reported in accordance with the Preferred Reporting Items for Systematic Reviews and Meta-analyses extension for Scoping Reviews (PRISMA-ScR) [34]. The development of the scoping review protocol was overseen by a multidisciplinary advisory group consisting of national and international experts in knowledge translation and NCD prevention from various academic institutions including The National Centre of Implementation Science (https://ncois.org.au/) and the Collaboration for Enhanced Research Impact (https://preventioncentre.org.au/resources/collaboration-for-enhanced-research-impact-ceri/). The protocol was prospectively deposited in Open Science framework at: 10.17605/OSF.IO/YJTN5 on 24th May, 2021 [35].

### Inclusion and exclusion criteria

This scoping review targeted the dissemination of knowledge outputs related to the prevention of NCDs. Specifically, we were interested in strategies aiming to disseminate knowledge outputs, relating to public health research evidence and/or interventions, to stakeholders and policy and practitioner end users (i.e., end of project-KT) and their potential influence on evidence use or adoption, and determinants thereof such as knowledge, motivation and awareness. Further details of the included populations of interest are described in Table 1. We included dissemination related outcomes based on an adapted version of the outcomes in Leeman et al’s (2017) framework [36]. These include:

- *Reach*: the number or proportion of individuals that information is disseminated to.
- *Awareness*: of the disseminated information.
- *Knowledge*: familiarity and understanding of the disseminated information.
- *Attitudes*: beliefs, feelings and behavioural tendencies about the disseminated information.
- *Preferences*: indication of a hypothetical choice for particular dissemination strategies over others.
- *Intention to adopt*: the probability of changing behaviour based on the disseminated information.
- *Research adoption or uptake*: if the disseminated information was used/implemented.
- *“Experiences” of dissemination*: data where participants reported which dissemination strategies (or components of Brownson’s model) they had previously used either to disseminate or to access disseminated information. For example, a sample of researchers reporting which channels were most commonly used for dissemination was considered as reporting experiences of dissemination. Although related to preferences, data suggests that preferred methods for dissemination do not always align with actual experiences [14], hence we treated these as two separate constructs.

Measures could be objective (e.g., audit data of a particular public health practice following dissemination) or subjective (e.g., self-reported use of disseminated research, or intentions to use).

#### Context

Given the breadth of public health as a field of research and the substantial burden of disease imposed by NCDs [31], we limited the context of studies included in this review to those examining the primary and/or secondary prevention [37] of NCDs defined as those on the Lancet’s Global Burden of Disease cause and risk summaries [38], such as cancer, cardiovascular diseases and mental disorders. Studies discussing dissemination in communicable, maternal, neonatal and nutritional diseases, as well as injuries were excluded as this review was primarily focused on NCDs. In addition, to ensure we comprehensively captured the evidence base in relation to the prevention of NCDs, studies in which more general perceptions and experiences of dissemination of public health evidence where NCD prevention were covered were also included.

**Table 1:** Details of included populations of interest

*Populations of interest*
*Public health practitioners*: healthcare providers responsible for the direct provision of primary or secondary prevention services (i.e. health promotion) to the general public. May include dietitians, community nurses and general practitioners (GPs).
*Public health manager and administrators*: individuals responsible for managing public health services including decisions regarding resource allocation.
*Researchers*: academic and clinician/public health researchers involved in the conduct and reporting of public health research.
*Research funders*: bodies, organisations (government or other) or individuals who provide funding to academic and/or health organisations to conduct research.
*Regulatory bodies*: government appointed organisations that oversee and/or provide recommendations or standards relevant to public health (such as food safety).
*Industry*: organisations that are affected by or have the capacity to influence public health policy and programs, or are involved in the delivery of public health. This may include the food and beverage industry, alcohol or tobacco manufacturers, or pharmaceutical companies.
*Policy makers*: those who are responsible for the development and revision of broad public health strategy, policy and practice, and monitor its implementation (such as those employed by government health departments).
*Politicians*: elected officials who have input into government policy and legislation decisions and are also responsible for the development and revision of broad public health strategy and policy.

#### Study designs

Given the broad aim of this scoping review, studies were not restricted by design. All empirical work was considered for inclusion including quantitative studies, which included cross-sectional, pre-post designs, controlled before after studies, quasi-randomised controlled trials, and randomised controlled trials (RCTS), as well as qualitative designs and mixed methods approaches including case studies. We excluded papers that did not provide new data, such as commentaries, editorials, letters to the editor, studies describing conceptual models or frameworks, and studies describing measurement tools.

### Search strategy

Given the well documented challenges with searching the knowledge translation literature and lack of consistency in terminology [20], we created a list of keywords and search terms used in previous reviews [39, 40] and used this to develop our search strategy in collaboration with an information specialist (see Supplementary Material 1). We searched Medline, Psycinfo, and EBSCO Search Ultimate:health, communications and business/marketing databases, up to 25th May, 2021. As a number of potentially relevant reports of dissemination studies were expected to be in the grey literature, we also searched Open Grey (https://opengrey.eu/) and key government public health websites in Australia, New Zealand, United Kingdom, Canada and the United States of America (USA). Consistent with Haddaway et al’s recommendation [41], we searched the top 200 results in Google and Google Scholar using the search terms dissemination strategy and public health. We also searched the reference lists of relevant evidence reviews to find additional primary studies. Following advice in the Cochrane handbook [42] we hand searched the Journal of Science Communication as it was not indexed in the databases searched. Our search was limited to studies published from January 2000 onwards following the Canadian Institutes of Health Research definition of knowledge translation, dissemination and implementation in 2000 [5] a substantial increase in work occurred in this area. Due to resource limitations, we excluded studies in which the full text was not available in English.

### Evidence screening and selection

Duplicate citations were removed in Endnote and an initial title screen was conducted (HT) to exclude studies that clearly did not meet the inclusion criteria (e.g., studies focusing on infectious disease). Remaining citations were uploaded into Covidence where title and abstract screening was conducted independently and in duplicate by two members of the review team (HT, NS, SO’C, SN, EW, SMc, AR, CH, SY). The full text of potentially relevant studies was sourced, and evaluated independently and in duplicate against the inclusion/exclusion criteria by two members of the review team (MF, HT, AR, NS, SY). Conflicts were resolved by discussion or through consultation with a third reviewer if needed.

### Data extraction

A data extraction template containing the data items was developed and piloted by members of the research team. This template was then used by two independent data extractors (HT, SN, ED, PL, EH, RS, SO’C). A third team member (SMc) was responsible for checking the extracted data. Any disagreements were resolved by the data extractors. The following data fields were extracted for studies deemed to meet inclusion criteria: citation details, study design, population group (policy makers, public health practitioners, community, etc.), sample size, country, setting (community, clinical or both), components related to dissemination (source, audience, message, channel), NCD or risk factor targeted (e.g., physical activity, skin cancer), and measures (awareness, knowledge, use etc.).

### Departures from protocol

Although we planned to include the general public as an end-user group, following our initial search we determined that empirical dissemination efforts to the general public differ substantially to those from the end users previously described including policymakers, practitioners, researchers, and public health administrators and often take the form of mass media campaigns or similar, which have been extensively reviewed [43, 44]. We also decided to exclude reviews due to significant overlap with primary studies already identified but we searched their reference lists for additional eligible studies.

In addition, we identified several studies in which dissemination was occurring to individuals or groups responsible for making decisions about program adoption in settings such as schools or community groups. Although these disseminated programs may have been aimed at individuals (e.g., a program to increase the physical activity level of children in schools), the dissemination activity was targeted at those who could make a decision regarding the program adoption in their setting. We included these studies as an additional population group of interest and classified this group as “community decision makers”. Examples included school principals, and workplace health committees.

### Data analysis

In order to describe the scope of the research base, identify gaps, and in accordance with JBI guidance for data analysis of scoping reviews [33], frequencies and percentages were calculated for year of publication, study design, study population, country of respondents, setting, NCD/risk factor focus, and outcomes assessed. We classified studies based on their design into three broad categories. Descriptive studies, which described the nature and determinants of dissemination, were further grouped into: (1) qualitative and mixed methods studies, and (2) quantitative, non-experimental studies including cross-sectional designs and case studies. The third category included experimental studies testing dissemination strategies. Only studies which explicitly aimed to directly compare different strategies within a component of dissemination (e.g., comparing two or more different channels, or comparing a strategy versus a control) were classified as experimental studies for the purpose of this scoping review. Studies in which a single dissemination strategy was implemented were coded based on the nature of data collection (e.g., qualitative vs. quantitative). Unless otherwise noted, percentages reported in text refer to the percentage across all study types. With respect to the dissemination components, all studies were coded based on the availability of information about each component in each study. For experimental studies, information about all four components was typically present, but usually only one component was deliberately manipulated as part of the study design and this has been described in text. Study findings were narratively described based on the component factors related to dissemination. We classified these by study design as we believed this classification would allow researchers to identify gaps in the types of evidence available to inform practice.

Given the large variety of channels available for dissemination of evidence, data were coded into broad categories. These categories were loosely organised based on different communication mediums. It should also be noted that some types of channels could be delivered in multiple ways. For example, information booklets and pamphlets could be mailed, emailed or provided in person. In these cases, we did not distinguish between mode of delivery when synthesising the data.

## Results

### Study selection

**Fig. 2:**
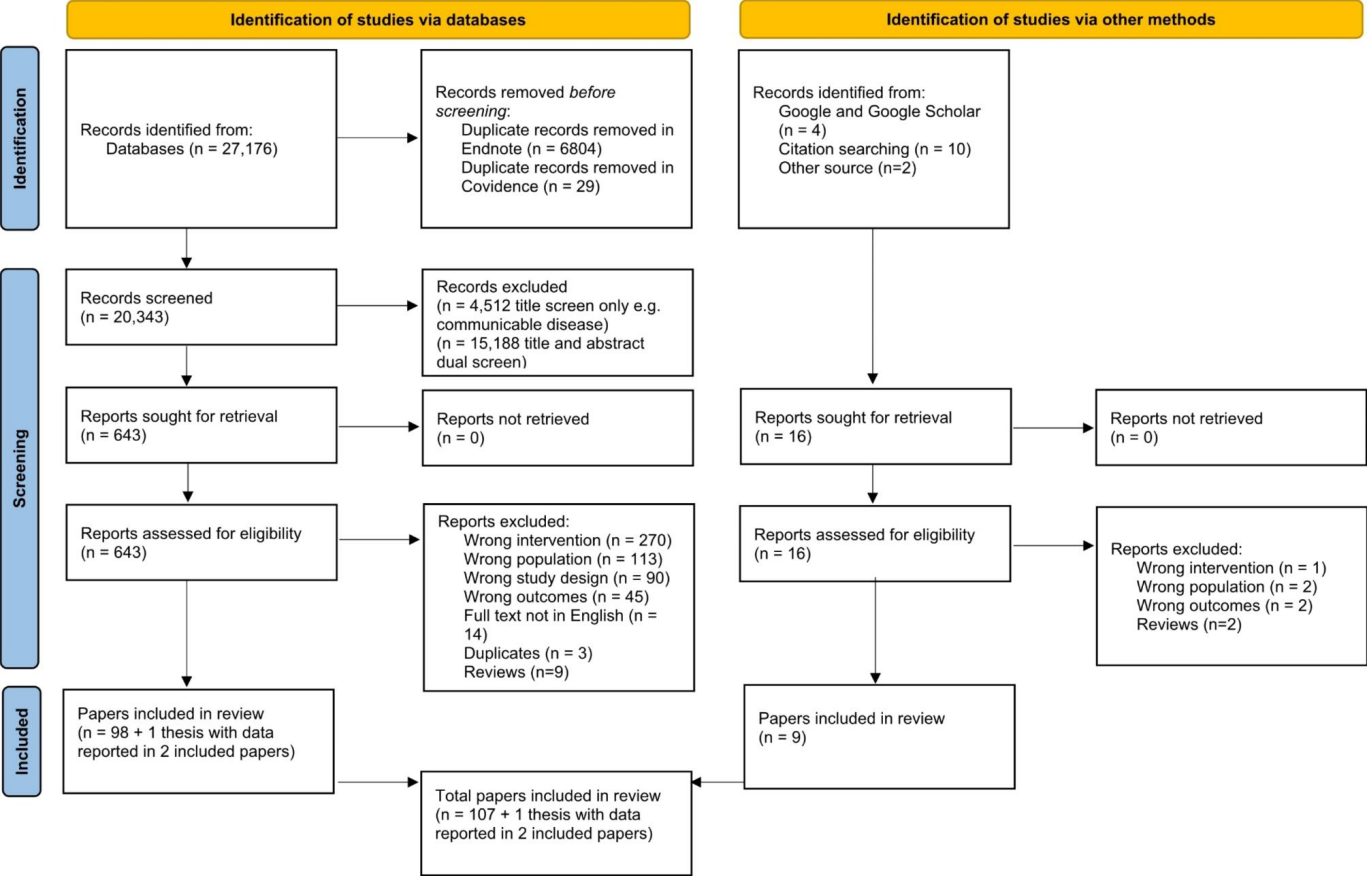
PRISMA flow diagram of studies

Following removal of duplicates, 20,343 records were screened based on their title or title and abstract, with 643 records progressing to full text screening. An additional 16 full text records identified through a manual search were also screened. Over half of the excluded studies were excluded due to wrong population or wrong intervention (see full list of excluded studies in Supplementary File 2). After full text screening, 107 studies (plus 1 thesis with data reported in 2 included studies) were selected for inclusion in the scoping review, as shown in Fig. 2.

## Characteristics of included studies

The number of studies published has increased over time. The number of publications identified in the last 10 years [period between 2011 and the time of our search (May 2021)] was 72, double that identified in the preceding period between 2000 and 2010 (n = 36).

As seen in Table 2, descriptive study designs were utilised in the majority of included studies, namely qualitative or mixed methods (n = 52, 49%) or quantitative (n = 40, 37%) study designs. Only 15 (14%) studies reported the findings of an experimental study. Please see Supplementary Material 3 for a list of references included in each design category. The most frequent countries where studies were conducted were the United States (n = 39, 36%) and Canada (n = 33, 31%), while there were few studies conducted in low or middle income countries (n = 9, 8%) and none of these were experimental studies.

The focus area or broad topic of dissemination was most commonly related to physical activity, diet or obesity (n = 44, 41%). Topics including cancer screening (n = 7, 7%), substance use (including smoking cessation; n = 23, 21%), and mental health (n = 11, 10%) were also covered. Some of the topics that were included in the “other” category included air pollution and skin cancer prevention, as well as chronic disease prevention. Within studies using qualitative, quantitative and mixed methods designs, a number of studies (n = 24, 26%) did not cover a specific focus area, but referred to dissemination of public health information in general. An example of this was studies in which public health researchers reported which channels they used to disseminate their research findings.

## Components of the Dissemination Framework

### Source

Source was conceptualised and reported in two main ways in the included studies. In many of the descriptive studies reporting preference data, the information about source was based on the types of information accessed or preferred by participants for obtaining evidence. For example, practitioners may have been asked to indicate which sources they most frequently contacted if evidence was required for a particular initiative (e.g.,[45]).

For the remainder of the descriptive studies and in the experimental studies, a specific source of the information to be disseminated was identifiable. As those most commonly generating the evidence (through research), researchers and academic groups were the most frequently identified sources (n = 35, 54%), followed by government bodies (n = 13, 29%) which were usually health departments. In some studies, multiple sources were identified. For example, researchers may have partnered with local public health bodies to disseminate research findings to stakeholders. In one study, Ferdinands et al. [46] described the involvement of an expert working group including researchers and practitioners, as well as partnership with local health services to develop and disseminate a nutrition report card on food environments for children. It was also common for individuals to take on multiple roles dependant on the nature of the evidence to be disseminated. For example, guideline development committees may include practitioners, researchers, and policymakers. Only one experimental study [47] manipulated the source as part of their study, in addition to manipulating the message and the channel.

A small number of studies, for example, those involving a survey of policymakers reporting only on channels accessed for dissemination, did not provide details or examples of the types of sources used, and therefore we were unable to categorise them in Table 2.

### Message

Across qualitative/mixed methods studies, the message or information that was the subject of dissemination was most commonly new knowledge or evidence on a specific topic (n = 21, 40%), often in the form of a review. The message was only clearly identifiable in 58% (n = 30) of the qualitative/mixed methods studies, primarily because participant preferences or barriers to dissemination were the focus of many studies. In these studies, dissemination of a defined message was not a study aim.

For the quantitative studies, in half of the studies (n = 20, 50%) the message was related to new knowledge or evidence. In some studies, the topic of the information to be disseminated varied across respondents (typically researchers), whereas for studies reporting on the outcomes of a dissemination effort the topic of information was more clearly defined. For example, the study by McVay et al. [19] reports the results from a survey of public health researchers in which they assessed how “research findings” were disseminated to local public health agencies. In contrast, Mattran et al. [48] provides an example of a study which describes the results of an effort to disseminate a report detailing state based physical activity data.

In experimental studies, dissemination of an evidence-based program or intervention made up a greater proportion of the messages (n = 6, 40%) compared to dissemination of knowledge/research evidence (n = 5, 33%) or guidelines (n = 4, 27%). The types of programs or interventions disseminated included sun protection [49, 50], workplace health including tobacco use prevention [47], and alcohol and substance abuse [51–53].

A small number of studies also described particular communication techniques, such as use of plain language, formatting (e.g., dot point summaries) and the types of information to include as part of disseminated evidence (e.g., inclusion of local data) that were preferred by different audiences (data not shown). For example, in a study with US legislators, Dodson and colleagues [54] found that there was interest in receiving cost data, as well as information about existing policies and what was occurring in other states when receiving evidence in order to inform policy decisions. In addition, three experimental studies manipulated aspects of the message as part of the design, such as the type or amount of detail provided in the dissemination materials [47, 49, 55].

### Channel

For both the qualitative/mixed methods and the quantitative studies, the most commonly reported channels included academic mediums such as journal articles and conferences (n = 25, 48% and n = 14, 35%), policy briefs (n = 13, 25% and n = 2, 5%), websites/infographics (n = 17, 33% and n = 17, 43%), information pamphlets/brochures (n = 9, 17% and n = 14, 35%), training/workshops (n = 19, 37% and n = 23, 58%), and one-on-one meetings (n = 12, 23% and n = 14, 35%). Many studies reported the use of multiple channels for communicating information, and this was particularly the case for studies in which multiple audiences were targeted. For example, studies targeting other researchers, practitioners and the public may have reported disseminating evidence through journal articles and conferences (targeting other researchers), workshops (targeting practitioners) and websites and media (targeting the public). The “other” category included channels such as knowledge brokers (which can also be considered a source) and institutional repositories or clearinghouses, as well as channels targeted at the general public such as prenatal classes, telephone helplines. Studies also presented channel information in the context of preferences or data about access frequency, rather than for the dissemination of a specific message, for example see [56, 57].

Focusing specifically on the experimental studies, channel was the most commonly manipulated component out of Brownson’s four dissemination components, with 13/15 (86%) studies comparing two or more dissemination channels (or dissemination vs. a control). Some studies compared different mediums of information presentation, for example, mailed information vs. a presentation [51–53, 58–61]. Others compared a basic dissemination strategy to an enhanced approach utilising multiple strategies [47, 49, 50, 62]. One study compared the provision of a booklet to a wait-list control [63], while a cluster RCT compared the dissemination of guidelines and relevant materials to experimental communities with control communities who received no information [64].

### Audience

Across all study designs, the most frequently identified audience was health practitioners (n = 61, 57%), such as GPs, nurses and allied health workers. For qualitative and mixed methods studies, policymakers (n = 24, 46%) and public health managers/administrators (n = 23, 44%) were targeted in almost as many studies as those targeting practitioners (n = 27, 52%). Many studies identified multiple audiences, which usually included at least one or more of these three groups, plus other relevant stakeholders such as public health managers, and researchers. For studies which disseminated evidence relevant to children and adolescents, stakeholders often included teachers, school principals and early childhood educators, which were categorised as community decision makers.

For the quantitative studies, over half of the studies (n = 24, 60%) identified practitioners as an audience, but only a third targeted policymakers (n = 13, 33%) and public health managers/administrators (n = 13, 33%). Experimental studies followed a similar pattern, with two thirds (n = 10, 67%) of the experimental studies identifying practitioners as their intended audience. Two experimental studies [55, 65], compared the effectiveness of a dissemination strategy across multiple audience groups.

As noted previously when considering channels, studies often tailored their dissemination channel to the relevant audiences. For example, Monnard and colleagues [66] described how they disseminated the findings from a large public health survey to a range of stakeholders including public health practitioners, academics and the broader community through a variety of mediums, such as community forums for the general public, and presentations and peer reviewed journal articles for researchers and public health professionals.

## Outcomes

Most studies reported findings across a variety of outcomes. Qualitative and mixed methods studies typically reported determinants of dissemination such as preferences (n = 31, 60%), and previous experiences of dissemination (e.g., what sources/channels are commonly accessed to find evidence, n = 36, 69%). In contrast, quantitative and experimental studies had a higher proportion of studies that reported outcomes that could be measured as a consequence of dissemination, such as awareness (n = 17, 31%), reach (n = 19, 35%), and intentions to use or apply the disseminated evidence (n = 20, 36%).

When considering studies that reported on the dissemination of a specific program or research evidence (i.e., all experimental studies and 44% and 70% of the qualitative/mixed methods studies and quantitative studies respectively), outcomes were generally reported from the post-dissemination phase only. A few studies reported increases in outcomes such as knowledge or awareness, based on changes in these outcomes from pre to post dissemination (e.g.,[65, 67]). Adoption/uptake of the disseminated information was the most frequently reported outcome (n = 57, 53%), while knowledge was the least frequently reported outcome (n = 21, 20%).

Standardised measures for outcomes were rarely used, with studies mostly using measures developed specifically for that study, prohibiting comparison of outcomes across studies. In addition, many studies provided minimal detail as to how outcomes were measured, further compounding difficulties in study comparisons.

**Table 2:** Attributes of studies (frequency counts and %), mapped by study design

		Qualitative/Mixed methods N = 52 n (%)	Quantitative studies N = 40 n (%)	Experimental N = 15 n (%)	Total n
Country*	Canada	22 (42)	8 (20)	3 (20)	33
	United States	9 (17)	22 (55)	8 (53)	39
	United Kingdom	6 (12)	2 (5)	4 (27)	12
	Australia	8 (15)	3 (8)	1 (7)	12
	Low and middle income countries	6 (12)	3 (8)	0 (0)	9
	Other high income countries	3 (6)	3 (8)	2 (13)	8
	Country/s not specified	3 (6)	1 (3)	0 (0)	4
Sample size (either of respondents or organisations/cases if not reported)	< 250	46 (88)	21 (53)	6 (40)	73
	≥250	4 (8)	15 (38)	9 (60)	28
	Unclear/not reported	2 (4)	4 (10)	0 (0)	6
Focus Area of disseminated information*	Physical Activity/Diet/Obesity	20 (38)	20 (50)	4 (27)	44
	Cancer screening	2 (4)	2 (5)	3 (20)	7
	Substance use	9 (17)	10 (25)	4 (27)	23
	Mental health	6 (12)	3 (8)	2 (13)	11
	Public health (general)	17 (33)	7 (18)	0 (0)	24
	Cardiovascular risk factors (e.g. hypertension)	1 (2)	3 (8)	0 (0)	4
	Other (including not explicitly specified)	6 (12)	2 (5)	2 (13)	10
Source (could be evidence producer or disseminator)*^#^	Researchers/Academics/Scientific Societies	23 (44)	23 (58)	12 (80)	58
	Government body/health department	18 (35)	11 (28)	2 (13)	31
	Guideline development groups	7 (13)	7 (18)	2 (13)	16
	Professional Societies	3 (6)	4 (10)	2 (13)	9
	Other	18 (35)	11 (28)	4 (27)	33
Message^#^	Evidence based program/practice (for interventions or programs)	2 (4)	12 (30)	6 (40)	20
	Guidelines (guidelines, codes or tools)	7 (13)	8 (20)	4 (27)	19
	Knowledge/research evidence/study findings including summaries	21 (40)	20 (50)	5 (33)	46
Channel*^	Academic outputs (i.e.,journal articles, conferences, reports)	25 (48)	14 (35)	0 (0)	39
	Policy briefs/summaries	13 (25)	2 (5)	1 (7)	16
	Information brochures/pamphlets/written materials	9 (17)	14 (35)	8 (53)	31
	Websites/infographics	17 (33)	17 (43)	3 (20)	37
	One-on-one meetings (in person or technology enabled)	12 (23)	14 (35)	5 (33)	31
	Training/workshop/presentations	19 (37)	23 (58)	6 (40)	48
	Media (traditional or social)	13 (25)	12 (30)	1 (7)	26
	Decision support tools or resources	6 (12)	16 (40)	5 (33)	27
	Other	22 (42)	12 (30)	4 (27)	38
Intended Audience*	Practitioners	27 (52)	24 (60)	10 (67)	61
	Policymakers	24 (46)	13 (33)	2 (13)	39
	Public Health managers/administrators	23 (44)	13 (33)	2 (13)	38
	Community Decision Makers	11 (21)	10 (25)	4 (27)	25
	General Public	10 (19)	7 (18)	1 (7)	18
	Researchers	12 (23)	7 (18)	0 (0)	19
	Others such as NGOs, advisory bodies	18 (35)	13 (33)	1 (7)	32
Outcomes*	Awareness	9 (17)	12 (30)	5 (33)	26
	Reach	9 (17)	17 (43)	2 (13)	28
	Attitudes	14 (27)	18 (45)	4 (27)	36
	Knowledge	11 (21)	6 (15)	4 (27)	21
	Intentions to adopt	9 (17)	12 (30)	8 (53)	29
	Adoption/uptake	22 (42)	24 (60)	11 (73)	57
	Preferences	31 (60)	8 (20)	1 (7)	40
	Experiences of dissemination	36 (69)	15 (38)	0 (0)	51

Note: * = categories not mutually exclusive; ^#^ = not all studies report details for this attribute, ^= for the purposes of this table, if a study reported the use of multiple channels within a single category (e.g. journal articles and conferences) this category was only counted once for that study

## Discussion

### Summary of key findings

This scoping review aimed to describe and map the literature examining dissemination of public health evidence related to the prevention of NCDs. Given the lack of consistency in the literature to describe dissemination studies, we intentionally used a broad approach to searching the literature. Our review used relatively extensive inclusion criteria including multiple study designs and covered two decades of published research, resulting in 107 studies being included in this review. Our findings are consistent with a recent scoping review of dissemination frameworks which identify variability of studies, inconsistencies and challenges with defining dissemination and few empirical studies applying dissemination specific frameworks [21].

#### There are opportunities to improve the “science” to determine ‘what works’ for dissemination

While the number of studies published in this area has increased in recent years, the results show that the majority continue to be descriptive studies examining general preferences and experiences of dissemination, or providing case study examples of dissemination efforts. Well controlled, experimental studies which compare dissemination strategies for communicating evidence are lacking, a finding echoed in several recent reviews [68, 69]. This is despite the extensive availability of rigorous evidence-based interventions that have demonstrated effectiveness (including cost-effectiveness) in the prevention of NCDs and reducing associated risk factors [70]. The findings of this review suggest it is not a lack of available evidence to be disseminated, but rather a lack of evidence to guide dissemination efforts. Additionally much of the literature has focused on the experience of dissemination rather than specific efforts to advance dissemination science.

Another area of the literature which appears to have substantial scope for future research is surrounding outcomes of dissemination. While most studies reported multiple outcomes, measures were typically poorly described and frequently collected only post-dissemination, limiting the potential to explore effectiveness of dissemination strategies. In addition, the most frequently reported measure was adoption/uptake. There is a significant opportunity for the science of dissemination outcome measurement to be improved, through more frequent measurement of key dissemination outcomes such as attitudes and knowledge [36], as well as the development of a measurement taxonomy, such as that developed for implementation research by Proctor et al.[71]. A recent review proposed a number of constructs including knowledge utilization, awareness, and changes in policy uptake, that are described in dissemination frameworks and that may be important outcomes to measure to assess the impact of dissemination strategies [21].

The topics which received the greatest amount of attention by dissemination researchers are in the areas of diet, physical activity, and obesity, followed by substance use, however the overall number of experimental research studies remains small across topics. This likely reflects the availability of high quality evidence in these areas for effective intervention approaches, as well as prevalence of risk factors. The limited number of studies exploring the dissemination of evidence related to cancer screening programs may be due to how the interventions are disseminated. Such interventions tend to be disseminated directly to individuals (i.e., members of the general public) for cancer screening such as through mass media campaigns [72, 73] and therefore were excluded from our scoping review due to the population of interest.

#### Increased variety of producers (sources) and end-users (audiences) in dissemination practice: going beyond researchers, policymakers and practitioners

Our scoping review revealed that researchers continue to be the primary disseminators (source) of evidence in this context. However, with an increased emphasis on co-production, and greater stakeholder involvement at all stages of the research cycle [74, 75], there is evidence of groups such as health departments, practitioners and professional bodies taking on the role as key sources of disseminated information. This is likely to be beneficial for increasing the reach and impact of evidence due to their perceived credibility. A previous review by the research team [76] has demonstrated the effect of using different messengers on improving implementation outcomes particularly in clinical settings. Although there were data from several descriptive studies suggesting some sources of evidence are preferred by policymakers compared to others [54, 77], there is a need for additional work to determine the effect of different sources on dissemination outcomes.

Most of the included studies targeted practitioners and/or policymakers as the identified audience. However, the scoping review revealed that there are a broad range of other stakeholders who may benefit from targeted dissemination even if they are not the primary users of the evidence. For example, groups such as politicians, advocacy groups, and professional associations may hold significant influence over decision makers and thus dissemination to these groups may prove fruitful in increasing eventual uptake of evidence. There is a need for greater evidence of the benefits of dissemination to other groups of stakeholders, as well as empirical data evaluating how manipulation of other components of dissemination (e.g., source and message) affects dissemination outcomes in different audiences.

One group that emerged as a key audience for dissemination was the ‘community decision makers’, such as principals and school teachers who have a role in determining whether and how evidence is adopted in their setting. We argue this is an important group to consider when developing, delivering, and evaluating strategies to disseminate evidence surrounding prevention of NCDs, especially given the abundance of programs focusing on this within community environments such as schools [2, 78, 79]. While groups such as public health officers, practitioners and policymakers can also influence evidence adoption in these settings (especially if acting as knowledge brokers), there may be advantages in undertaking targeted dissemination efforts to community decision makers who are embedded within the setting itself. Indeed, inclusion of all relevant end-users as part of the dissemination planning and roll-out process is a critical part of a co-creation approach to public health research [80] and improving the impact of dissemination. [81]

#### Most evidence focuses on the channel of dissemination, but clarifying the dissemination message can be difficult

Two broad aspects of the message were elicited through the scoping review: firstly, the type of evidence disseminated (e.g., a guideline, research synthesis, program/intervention), and secondly, what features of language and formatting are included as part of that communication (e.g., dot points, lay person language, presentation of local/contextual data). As the health communication literature has extensively explored this latter aspect (see for example [82, 83]), we primarily focused on the types of evidence being disseminated. Evidence or research summaries were the most common types of evidence to be disseminated in qualitative and quantitative studies, whereas evidence-based interventions/programs were disseminated in just under half the experimental studies. Of the four dissemination components, message was the most difficult to classify as some of the studies did not explicitly describe what the message was, particularly studies which used surveys or interviews examining broader experiences of dissemination. The message was much clearer to identify in studies in which a specific dissemination strategy had been enacted.

Channel appeared to receive the most attention in terms of exploration in the dissemination literature, and it dominated the experimental studies as the component most likely to be manipulated and compared in terms of effect on dissemination outcome. There is an extensive variety of available dissemination channels and mediums, and many of the studies included in this scoping review utilised multiple channels. Not surprisingly, methods targeted at fellow researchers such as peer reviewed articles and conferences were one of the most common cited channels, as well as training/workshops/presentations, which is consistent with other studies that have explored dissemination strategies utilised by researchers [84]. The decision of which channel/s to use is informed by a number of factors including cost, familiarity, access, experience, as well as other components of dissemination such as the target audience and the source [85]. This can make efforts to evaluate channel effectiveness complex, and while targeting of the channel is well acknowledged as essential in the literature [86, 87], further practical guidance based on empirical evidence would be beneficial.

### Strengths and limitations of the review

This scoping review has several strengths including the use of systematic and robust methods, from the prospective registration of the protocol, an extensive and comprehensive literature search and a dual independent screening process. We used an evidence based framework [10] to map the review findings, which has resulted in the first scoping review we are aware of that maps the evidence for dissemination in the prevention of NCD in the field of public health.

A common limitation within the dissemination literature is that dissemination as a field lacks clarity, with blurred boundaries of what constitutes dissemination compared to implementation and scale up more generally [21]. The terms used to describe dissemination studies in the literature are numerous, and selecting the most efficient yet inclusive search strategy remains challenging. Despite undertaking a systematic search, relevant studies may have been missed.

There is also some level of overlap between dissemination as we have included in this review and the related disciplines of health communication, scale up and social marketing. For example, much work has been done on message framing (e.g., gain vs. loss, [88]) but typically these studies have focussed on how health messages are communicated to patients and/or the public. Some studies on this topic may have been relevant, however exploring this vast literature was beyond the scope of this review.

Lastly, there are additional attributes that could have been extracted from included studies, such as whether the dissemination strategy used was informed by a specific theory. However, the level of detail of reporting of many attributes was extremely variable, which may be related to the broad range of study designs included. As the aim of this review was to broadly map the dissemination literature covering the prevention of NCDs, we focused on those attributes most commonly reported and could most comprehensively describe the scope of the literature. Examining attributes such as the role of theory in development of dissemination strategy could be a worthy focus of future systematic reviews. There is also opportunity to explore a number of topics in greater detail, such as the evolution of strategies over time, and by sub-groups. For example, are some channels used more frequently for communication by particular sources compared to others.

## Conclusion

In summary, this review has mapped the broad scope of the literature examining dissemination of evidence relevant to prevention of NCDs since 2000. It has identified a substantial base of qualitative and quantitative work, and opportunities for future experimental work. While there is a solid foundation of evidence when it comes to “what works” for the prevention of NCDs, there is much still to be learnt in order to determine “what works” to disseminate this evidence most effectively. If we are to reduce the evidence-practice gap in this area of public health, we need greater understanding of how to disseminate most effectively with each relevant end-user group; the audience who needs to receive the evidence. In particular, there is a need to determine the source that should deliver the information, how the message can be framed, and what channels are most appropriate for communication of the message to the audience.

## Electronic supplementary material

Below is the link to the electronic supplementary material.

Supplementary Material 1

Supplementary Material 2

Supplementary Material 3

## Data Availability

Data and materials relating to this review are available from the corresponding author on reasonable request.

## List of abbreviations

GP: general practitioner
KT: knowledge translation
NCD: non-communicable disease
NGO: non government organisation
RCT: Randomised controlled trial
US: United States
