# Supplementary material for: Dissemination of public health research to prevent non-communicable diseases: a scoping review
Source: BMC Public Health. 2023 Apr 24;23:757. doi: 10.1186/s12889-023-15622-x (PMC10123991; doi:10.1186/s12889-023-15622-x)
Supplement: Supplementary file 1 — Supplementary Material 1 [file 12889_2023_15622_MOESM1_ESM.docx]

**Dissemination of public health research to prevent non-communicable diseases: A scoping review**

Supplementary Material 1

Table 1: Search strategy

Medline/Psycinfo

| **Set** | **Search Statement** |
| --- | --- |
| 1. | Public health/ or public health.tw. |
| 2. | Community Health Services/ or community health.tw. |
| 3. | Health promotion/ |
| 4. | health promot*.tw. |
| 5. | 1 or 2 or 3 or 4 |
| 6. | "Diffusion of Innovation"/ |
| 7. | diffusion of innovation.tw. |
| 8. | Information Dissemination/ or information dissemination.tw. |
| 9. | research sharing.tw. |
| 10. | research trans*.tw. |
| 11. | data sharing.tw. |
| 12. | data trans*.tw. |
| 13. | information sharing.tw. |
| 14. | information trans*.tw. |
| 15. | knowledge sharing.tw. |
| 16. | knowledge trans*.tw. |
| 17. | Evidence-Based Medicine/ed, mt [Education, Methods] |
| 18. | Information Services/sn [Statistics & Numerical Data] |
| 19. | Practice Guidelines as Topic/st [Standards] |
| 20. | Social Marketing/ |
| 21. | Social Marketing.tw. |
| 22. | academic detailing.tw. |
| 23. | dissemination strateg*.tw. |
| 24. | disseminat*.tw. |
| 25. | Health Communication/ or health communication.tw. |
| 26. | 6 or 7 or 8 or 9 or 10 or 11 or 12 or 13 or 14 or 15 or 16 or 17 or 18 or 19 or 20 or 21 or 22 or 23 or 24 or 25 |
| 27. | knowledge.tw. |
| 28. | Reach.tw. |
| 29. | Adopt*.tw. |
| 30. | research utili*.tw. |
| 31. | uptake.tw. |
| 32. | Health Knowledge, Attitudes, Practice/ |
| 33. | motivat*.tw. |
| 34. | intention*.tw. |
| 35. | attitud*.tw. |
| 36. | awareness.tw. |
| 37. | 27 or 28 or 29 or 30 or 31 or 32 or 33 or 34 or 35 or 36 |
| 38. | exp animals/ not humans.sh. |
| 39. | 5 and 26 and 37 |
| 40. | limit 39 to yr="2000 -Current" |
| 41. | 40 not 38 |

EBSCO Ultimate

| S36 | S7 AND S24 AND S35 |
| --- | --- |
| S35 | S25 OR S26 OR S27 OR S28 OR S29 OR S30 OR S31 OR S32 OR S33 OR S34 |
| S34 | TI awareness OR AB awareness |
| S33 | TI attitud* OR AB attitud* |
| S32 | TI intention* OR AB intention* |
| S31 | TI motivat* OR AB motivat* |
| S30 | "Health Knowledge, Attitudes, Practice" |
| S29 | TI uptake OR AB uptake |
| S28 | TI research utili* OR AB research utili*. |
| S27 | TI Adopt* OR AB Adopt* |
| S26 | TI reach OR AB Reach |
| S25 | TI knowledge OR AB knowledge |
| S24 | S8 OR S9 OR S10 OR S11 OR S12 OR S13 OR S14 OR S15 OR S16 OR S17 OR S18 OR S19 OR S20 OR S21 OR S22 OR S23 |
| S23 | TI health communication OR AB health communication |
| S22 | "health communication" |
| S21 | TI disseminat* OR AB disseminat* |
| S20 | TI dissemination strateg* OR AB dissemination strateg* |
| S19 | TI academic detailing OR AB academic detailing |
| S18 | TI social marketing OR AB social marketing |
| S17 | (MH "Social Marketing") |
| S16 | (MH "Practice Guidelines/ST") |
| S15 | (MH "Information Services/MT/UT") |
| S14 | (MH "Medical Practice, Evidence-Based/ED/MT") |
| S13 | TI ( (research or data or information or knowledge) trans* ) OR AB ( (research or data or information or knowledge) trans* ) |
| S12 | TI ( (research or data or information or knowledge) sharing ) OR AB ( (research or data or information or knowledge) sharing ) |
| S11 | TI information dissemination OR AB information dissemination |
| S10 | (MH "Selective Dissemination of Information") |
| S9 | TI diffusion of innovation OR AB diffusion of innovation |
| S8 | (MH "Diffusion of Innovation") |
| S7 | S1 OR S2 OR S3 OR S4 OR S5 OR S6 |
| S6 | TI health promot* OR AB health promot* |
| S5 | community health |
| S4 | public health |
| S3 | (MH "Health Promotion") |
| S2 | (MH "Community Health Services") |
| S1 | (MH "Public Health") |
