# Supplementary material for: Dissemination of public health research to prevent non-communicable diseases: a scoping review
Source: BMC Public Health. 2023 Apr 24;23:757. doi: 10.1186/s12889-023-15622-x (PMC10123991; doi:10.1186/s12889-023-15622-x)
Supplement: Supplementary file 2 — Supplementary Material 2 [file 12889_2023_15622_MOESM2_ESM.docx]

**Supplementary Material 2 – Table of excluded studies from full text review and reason for exclusion**

| Study | Title | Authors | Published Year | Journal | Notes |
| --- | --- | --- | --- | --- | --- |
| Aarons 2012 | The organizational social context of mental health services and clinician attitudes toward evidence-based practice: a United States national study | Aarons, Gregory A.; Glisson, Charles; Green, Phillip D.; Hoagwood, Kimberly; Kelleher, Kelly J.; Landsverk, John A. | 2012 | Implementation Science | Exclusion reason: Wrong population; |
| Aaserud 2005 | Translating research into policy and practice in developing countries: a case study of magnesium sulphate for pre-eclampsia | Aaserud, Morten; Lewin, Simon; Innvaer, Simon; Paulsen, Elizabeth J.; Dahlgren, Astrid T.; Trommald, Mari; Duley, Lelia; Zwarenstein, Merrick; Oxman, Andrew D. | 2005 | BMC Health Services Research | Exclusion reason: Wrong intervention; |
| AbdulRahman 2011 | Knowledge sharing practices: A case study at Malaysiaâ€™s healthcare research institutes | Abdul Rahman, Ramlee | 2011 |  | Exclusion reason: Wrong population; |
| Abekah-Nkrumah 2018 | A review of the process of knowledge transfer and use of evidence in reproductive and child health in Ghana | Abekah-Nkrumah, Gordon; Issiaka, SombiÃ©; Virgil, Lokossou; Ermel, Johnson | 2018 | Health Research Policy & Systems | Exclusion reason: Wrong intervention; |
| Abildso 2019 | Adoption and Reach of a statewide policy, systems, and environment intervention to increase access to fresh fruits and vegetables in West Virginia | Abildso, C. G.; Bias, T. K.; Coffman, J. | 2019 | Translational Behavioral Medicine | Exclusion reason: Wrong intervention; |
| Abrahamson 2009 | Health and social care responses to the Department of Health Heatwave Plan | Abrahamson, V.; Raine, R. | 2009 | Journal of Public Health | Exclusion reason: Wrong intervention; |
| Abril 2015 | Mapping the Health Communication Infrastructure in Rural Senegal: an Assessment to Support Cervical Cancer Screening | Abril, EulÃ lia P.; Kupczyk, Magdalena; Zwicke, Grant L.; Mastarone, Ginnifer L.; Irwin, Tracy; Dykens, Andrew | 2015 | Journal of Applied Communication Research | Exclusion reason: Wrong population; |
| Acolet 2007 | The BLISS cluster randomised controlled trial of the effect of 'activedissemination of information' on standards of care for prematurebabies in England (BEADI) study protocol [ISRCTN89683698] | Acolet, Dominique; Jelphs, Kim; Davidson, Deborah; Peck, Edward; Clemens, Felicity; Houston, Rosie; Weindling, Michael; Lavis, John; Elbourne, Diana | 2007 | Implementation Science | Exclusion reason: Wrong intervention; |
| Adily 2004 | Use of on-line evidence databases by Australian public health practitioners | Adily, A.; Westbrook, J.; Coiera, E.; Ward, J. | 2004 | Medical Informatics & the Internet in Medicine | Exclusion reason: Wrong population; |
| Affret 2020 | Development of a knowledge translation taxonomy in the field of health prevention: a participative study between researchers, decision-makers and field professionals | Affret, A.; Prigent, O.; Porcherie, M.; Aromatario, O.; Cambon, L. | 2020 | Health Research Policy & Systems | Exclusion reason: Wrong study design; |
| Ahmad 2004 | Popular health promotion strategies among Chinese and East Indian immigrant women | Ahmad, F.; Shik, A.; Vanza, R.; Cheung, A.; George, U.; Stewart, D. E. | 2004 | Women & Health | Exclusion reason: Wrong population; |
| Ahuja 2018 | Challenges faced with the implementation of Web-Based Data Query Systems for population health: development of a questionnaire based on expert consensus | Ahuja, Manik; Aseltine, Robert; Warren, Nicholas; Reisine, Susan; Williams, Pam Holtzclaw; Cislo, Andy | 2018 | Pilot & Feasibility Studies | Exclusion reason: Wrong intervention; |
| Aikman 2020 | Mass media campaigns for chronic pain: a scoping review to inform design of future campaigns | Aikman, Kelda; Burtt, Lyndie; Ronde, Olivia de; Lim, Dave K. W.; Stratton, Paige; Wong, Man Hon; Grainger, Rebecca; Devan, Hemakumar | 2020 | Physical Therapy Reviews | Exclusion reason: Wrong population; |
| Ainsworth 2012 | eLab: bringing together people, data and methods to enhance knowledge discovery in healthcare settings | Ainsworth, J.; Cunningham, J.; Buchan, I. | 2012 | Studies in Health Technology & Informatics | Exclusion reason: Wrong intervention; |
| Aittasalo 2007 | From innovation to practice: initiation, implementation and evaluation of a physician-based physical activity promotion programme in Finland | Aittasalo, M.; Miilunpalo, S.; Stahl, T.; Kukkonen-Harjula, K. | 2007 | Health Promotion International | Exclusion reason: Wrong intervention; |
| Albrecht 2016 | Systematic Review of Knowledge Translation Strategies to Promote Research Uptake in Child Health Settings | Albrecht, L.; Archibald, M.; Snelgrove-Clarke, E.; Scott, S. D. | 2016 | Journal of Pediatric Nursing | Exclusion reason: Wrong intervention; |
| Alford 2009 | Promoting substance use education among generalist physicians: an evaluation of the Chief Resident Immersion Training (CRIT) program | Alford, Daniel P.; Bridden, Carly; Jackson, Angela H.; Saitz, Richard; Amodeo, Maryann; Barnes, Henrietta N.; Samet, Jeffrey H. | 2009 | JGIM: Journal of General Internal Medicine | Exclusion reason: Wrong intervention; |
| Allen 2013 | Promoting state health department evidence-based cancer and chronic disease prevention: a multi-phase dissemination study with a cluster randomized trial component | Allen, P.; Sequeira, S.; Jacob, R. R.; Hino, A. A.; Stamatakis, K. A.; Harris, J. K.; Elliott, L.; Kerner, J. F.; Jones, E.; Dobbins, M.; Baker, E. A.; Brownson, R. C. | 2013 | Implementation Science | Exclusion reason: Wrong study design; |
| Allen 2018 | Twitter use at the 2016 Conference on the Science of Dissemination and Implementation in Health: analyzing #DIScience16 | Allen, Caitlin G.; Andersen, Brittany; Chambers, David A.; Groshek, Jacob; Roberts, Megan C. | 2018 | Implementation Science | Exclusion reason: Wrong intervention; |
| Allender 2011 | The development of a network for community-based obesity prevention: the CO-OPS Collaboration | Allender, Steven; Nichols, Melanie; Foulkes, Chad; Reynolds, Rebecca; Waters, Elizabeth; King, Lesley; Gill, Tim; Armstrong, Rebecca; Swinburn, Boyd | 2011 | BMC Public Health | Exclusion reason: Wrong study design; |
| Amed 2016 | Wayfinding the Live 5-2-1-0 Initiative-At the Intersection between Systems Thinking and Community-Based Childhood Obesity Prevention | Amed, S.; Shea, S.; Pinkney, S.; Wharf Higgins, J.; Naylor, P. J. | 2016 | International Journal of Environmental Research & Public Health [Electronic Resource] | Exclusion reason: Wrong intervention; |
| Amsel 2005 | Training Therapists to Treat the Psychological Consequences of Terrorism: Disseminating Psychotherapy Research and Researching Psychotherapy Dissemination | Amsel, Lawrence V.; Neria, Yuval; Marshall, Randall D.; Eun Jung, Suh | 2005 | Journal of Aggression, Maltreatment & Trauma | Exclusion reason: Wrong population; |
| Andersen 2016 | Evidence-based psychosocial treatment in the community: considerations for dissemination and implementation | Andersen, Barbara L.; Dorfman, Caroline S. | 2016 | Psycho-Oncology | Exclusion reason: Wrong study design; |
| Anderson 2017 | Brookings supports breastfeeding: using public deliberation as a community-engaged approach to dissemination of research | Anderson, J.; Kuehl, R. A.; Mehltretter Drury, S. A.; Tschetter, L.; Schwaegerl, M.; Yoder, J.; Gullickson, H.; Lamp, J.; Bachman, C.; Hildreth, M. | 2017 | Translational Behavioral Medicine | Exclusion reason: Wrong population; |
| AndrÃ©asson 2000 | IMPLEMENTATION AND DISSEMINATION OF METHODS FOR PREVENTION OF ALCOHOL PROBLEMS IN PRIMARY HEALTH CARE: A FEASIBILITY STUDY | AndrÃ©asson, Sven; Hjalmarsson, Kerstin; Rehnman, Charlotta | 2000 | Alcohol & Alcoholism | Exclusion reason: Wrong intervention; |
| Andrade 2020 | Knowledge translation in the reality of Brazilian public health | Andrade, K. R. C.; Pereira, M. G. | 2020 | Revista de Saude Publica | Exclusion reason: Wrong study design; |
| Antle 2010 | Training the child welfare workforce in healthy couple relationships: An examination of attitudes and outcomes | Antle, Becky F.; Frey, Shannon E.; Sar, Bibhuti K.; Barbee, Anita P.; van Zyl, Michiel A. | 2010 | Children & Youth Services Review | Exclusion reason: Wrong intervention; |
| An-Wen 2014 | Increasing value and reducing waste: addressing inaccessible research | An-Wen, Chan; Fujian, Song; Vickers, Andrew; Jefferson, Tom; Dickersin, Kay; GÃ¸tzsche, Peter C.; Krumholz, Harlan M.; Ghersi, Davina; van der Worp, H. Bart | 2014 | Lancet | Exclusion reason: Wrong study design; |
| Armstrong 2014 | Shifting sands-From descriptions to solutions | Armstrong, R.; Pettman, T.; Waters, E. | 2014 | Public Health | Exclusion reason: Wrong intervention; |
| Aslani 2015 | KEY ITEMS OF INNOVATION MANAGEMENT IN THE PRIMARY HEALTHCARE CENTRES CASE STUDY: FINLAND | Aslani, Alireza; Zolfagharzadeh, Mohammad Mahdi; Naaranoja, Marja | 2015 | Central European Journal of Public Health | Exclusion reason: Wrong intervention; |
| Backer 2000 | The failure of success: Challenges of disseminating effective substance abuse prevention programs | Backer, Thomas E. | 2000 | Journal of Community Psychology | Exclusion reason: Wrong study design; |
| Barac 2014 | Scoping review of toolkits as a knowledge translation strategy in health | Barac, Raluca; Stein, Sherry; Bruce, Beth; Barwick, Melanie | 2014 | BMC Medical Informatics & Decision Making | Exclusion reason: Wrong population; |
| Barbour 2018 | Communities of practice to improve public health outcomes: A systematic review | Barbour, Liza; Armstrong, Rebecca; Condron, Patrick; Palermo, Claire | 2018 | Journal of Knowledge Management | Exclusion reason: Wrong intervention; |
| Barker 2003 | Order from Chaos: Organizational Aspects of Information, Education, and Communication (a Case Study from Mali) | Barker, Kriss | 2003 | Journal of Health Communication | Exclusion reason: Wrong intervention; |
| Barnett 2017 | Collaboration with deaf communities to conduct accessible health surveillance | Barnett, Steven L.; Matthews, Kelly A.; Sutter, Erika J.; DeWindt, Lori A.; Pransky, Jacqueline A.; O'Hearn, Amanda M.; David, Tamala M.; Pollard, Robert Q.; Samar, Vincent J.; Pearson, Thomas A. | 2017 | American Journal of Preventive Medicine | Exclusion reason: Wrong population; |
| Bartholomew 2009 | Getting clinical trial results into practice: design, implementation, and process evaluation of the ALLHAT Dissemination Project | Bartholomew, L. K.; Cushman, W. C.; Cutler, J. A.; Davis, B. R.; Dawson, G.; Einhorn, P. T.; Graumlich, J. F.; Piller, L. B.; Pressel, S.; Roccella, E. J.; Simpson, L.; Whelton, P. K.; Williard, A.; Allhat Collaborative Research, Group | 2009 | Clinical Trials | Exclusion reason: Wrong intervention; |
| Bartlett 2016 | Physicians and knowledge translation of statistics: Mind the gap | Bartlett, Gillian; Gagnon, Justin | 2016 | CMAJ: Canadian Medical Association Journal | Exclusion reason: Wrong study design; |
| Bartonova 2012 | How can scientists bring research to use: the HENVINET experience | Bartonova, A. | 2012 | Environmental Health: A Global Access Science Source | Exclusion reason: Wrong study design; |
| Barwick 2012 | Knowledge Translation Efforts in Child and Youth Mental Health: A Systematic Review | Barwick, MelanieA; Schachter, HowardM; Bennett, LindsayM; McGowan, Jessie; Ly, Mylan; Wilson, Angela; Bennett, Kathryn; Buchanan, DonH; Fergusson, Dean; Manion, Ian | 2012 | Journal of Evidence-Based Social Work | Exclusion reason: Wrong intervention; |
| Basch 2015 | A Randomized Trial to Compare Alternative Educational Interventions to Increase Colorectal Cancer Screening in a Hard-to-Reach Urban Minority Population with Health Insurance | Basch, Charles; Zybert, Patricia; Wolf, Randi; Basch, Corey; Ullman, Ralph; Shmukler, Celia; King, Fionnuala; Neugut, Alfred; Shea, Steven | 2015 | Journal of Community Health | Exclusion reason: Wrong intervention; |
| Bazyk 2015 | Building Capacity of Occupational Therapy Practitioners to Address the Mental Health Needs of Children and Youth: A Mixed-Methods Study of Knowledge Translation | Bazyk, S.; Demirjian, L.; LaGuardia, T.; Thompson-Repas, K.; Conway, C.; Michaud, P. | 2015 | American Journal of Occupational Therapy | Exclusion reason: Wrong intervention; |
| Bean 2020 | A winning combination: Collaborating with stakeholders throughout the process of planning and implementing a type 2 diabetes prevention programme in the community | Bean, C.; Sewell, K.; Jung, M. E. | 2020 | Health & Social Care in the Community | Exclusion reason: Wrong study design; |
| Beardslee 2013 | Dissemination of family-centered prevention for military and veteran families: adaptations and adoption within community and military systems of care | Beardslee, W. R.; Klosinski, L. E.; Saltzman, W.; Mogil, C.; Pangelinan, S.; McKnight, C. P.; Lester, P. | 2013 | Clinical Child & Family Psychology Review | Exclusion reason: Wrong study design; |
| Belanger-Gravel 2018 | A theory of planned behaviour perspective on practitioners' beliefs toward the integration of the WIXX communication campaign messages and activities into daily practice | Belanger-Gravel, A.; Lottinville, S.; Beaurivage, D.; Laferte, M.; Therrien, F.; Gauvin, L. | 2018 | Public Health | Exclusion reason: Wrong intervention; |
| Bellows 2007 | Adoption of system strategies for tobacco cessation by state medicaid programs | Bellows, N. M.; McMenamin, S. B.; Halpin, H. A. | 2007 | Medical Care | Exclusion reason: Wrong intervention; |
| Benedetti 2020 | Re-thinking Physical Activity Programs for Older Brazilians and the Role of Public Health Centers: A Randomized Controlled Trial Using the RE-AIM Model | Benedetti, T. R. B.; Rech, C. R.; Konrad, L. M.; Almeida, F. A.; Brito, F. A.; Chodzko-Zajko, W.; Schwingel, A. | 2020 | Frontiers in Public Health | Exclusion reason: Wrong intervention; |
| Bennett 2009 | The Delivery of Public Health Interventions via the Internet: Actualizing Their Potential | Bennett, Gary V.; Galsgow, Russell E. | 2009 | Annual Review of Public Health | Exclusion reason: Wrong population; |
| Beran 2015 | Health systems research for policy change: lessons from the implementation of rapid assessment protocols for diabetes in low- and middle-income settings | Beran, David; Miranda, J. Jaime; Cardenas, Maria Kathia; Bigdeli, Maryam | 2015 | Health Research Policy & Systems | Exclusion reason: Wrong intervention; |
| Bernhardsson 2014 | Evaluation of a tailored, multi-component intervention for implementation of evidence-based clinical practice guidelines in primary care physical therapy: a non-randomized controlled trial | Bernhardsson, Susanne; Larsson, Maria E. H.; Eggertsen, Robert; Fagevik OlsÃ©n, Monika; Johansson, Kajsa; Nilsen, Per; Nordeman, Lena; van Tulder, Maurits; Ã–berg, Birgitta | 2014 | BMC Health Services Research | Exclusion reason: Wrong intervention; |
| Bernstein 2009 | A preliminary report of knowledge translation: lessons from taking screening and brief intervention techniques from the research setting into regional systems of care | Bernstein, E.; Topp, D.; Shaw, E.; Girard, C.; Pressman, K.; Woolcock, E.; Bernstein, J. | 2009 | Academic Emergency Medicine | Exclusion reason: Wrong intervention; |
| Bhagia 2020 | Effectiveness of various health education methods amongst primary healthcare workers of western Uttar Pradesh, Delhi (National Capital Region), India: A promotive intervention study | Bhagia, Preeti; Menon, Ipseeta; Singh, Ricky; Gupta, Ritu; Goyal, Jyoti; Das, Dipshikha | 2020 | Journal of Family Medicine & Primary Care | Exclusion reason: Wrong intervention; |
| Blair 2014 | Getting evidence into practice--implementation science for paediatricians | Blair, Mitch | 2014 | Archives of Disease in Childhood | Exclusion reason: Wrong study design; |
| Blaschke 2008 | Choosing the Bright Futures Guidelines: lessons from leaders and early adopters | Blaschke, G. S.; Lopreiato, J. O.; Bedingfield, B.; Rash, F. C.; Burke, A. E.; Goldstein, R.; Shope, T. R.; Johnson, C.; Biagioli, F. E.; Beers, N. S.; Hagan, J. F., Jr. | 2008 | Pediatric Annals | Exclusion reason: Wrong study design; |
| Bobitt 2020 | Using diffusion of innovations framework to examine the dissemination and implementation of the adult protective services national voluntary consensus guidelines | Bobitt, Julie; Carter, Julie; Kuhne, Jamie | 2020 | Journal of Elder Abuse & Neglect | Exclusion reason: Wrong population; |
| Boddy 2019 | What does healthy eating mean? Australian teachers' perceptions of healthy eating in secondary school curricula | Boddy, Gail; Booth, Alison; Worsley, Anthony | 2019 | Health Education | Exclusion reason: Wrong study design; |
| Boland 2019 | Evaluation of a shared decision making educational program: The Ottawa Decision Support Tutorial | Boland, Laura; LÃ©garÃ©, France; Carley, Meg; Graham, Ian D.; O'Connor, Annette M.; Lawson, Margaret L.; Stacey, Dawn | 2019 | Patient Education & Counseling | Exclusion reason: Wrong intervention; |
| Boland 2020 | Building an integrated knowledge translation (IKT) evidence base: colloquium proceedings and research direction | Boland, L.; Kothari, A.; McCutcheon, C.; Graham, I. D.; Integrated Knowledge Translation Research, Network | 2020 | Health Research Policy & Systems | Exclusion reason: Wrong intervention; |
| Bonder 2020 | Putting positive weightâ€related conversations into practice: The pilot implementation of a Knowledge Translation Casebook | Bonder, Revi; Provvidenza, Christine F.; Hubley, Darlene; McPherson, Amy C. | 2020 | Child: Care, Health & Development | Exclusion reason: Wrong intervention; |
| Bonner 2019 | Implementing cardiovascular disease prevention guidelines to translate evidence-based medicine and shared decision making into general practice: theory-based intervention development, qualitative piloting and quantitative feasibility | Bonner, C.; Fajardo, M. A.; Doust, J.; McCaffery, K.; Trevena, L. | 2019 | Implementation Science | Exclusion reason: Wrong intervention; |
| Bornbaum 2015 | Erratum to: 'Exploring the function and effectiveness of knowledge brokers as facilitators of knowledge translation in health related settings: a systematic review and thematic analysis' | Bornbaum, Catherine C.; Kornas, Kathy; Peirson, Leslea; Rosella, Laura C. | 2015 | Implementation Science | Exclusion reason: Duplicate; |
| Bornbaum 2015 | Exploring the function and effectiveness of knowledge brokers as facilitators of knowledge translation in health-related settings: a systematic review and thematic analysis | Bornbaum, Catherine C.; Kornas, Kathy; Peirson, Leslea; Rosella, Laura C. | 2015 | Implementation Science | Exclusion reason: Wrong population; |
| Bornstein 2017 | Putting research in place: an innovative approach to providing contextualized evidence synthesis for decision makers | Bornstein, S.; Baker, R.; Navarro, P.; Mackey, S.; Speed, D.; Sullivan, M. | 2017 | Systematic Reviews | Exclusion reason: Wrong outcomes; |
| Borst 2019 | Envisioning and shaping translation of knowledge into action: A comparative case-study of stakeholder engagement in the development of a European tobacco control tool | Borst, Robert A. J.; Kok, Maarten Olivier; O'Shea, Alison J.; Pokhrel, Subhash; Jones, Teresa H.; Boaz, Annette | 2019 | Health Policy | Exclusion reason: Wrong intervention; |
| Bou-Karroum 2017 | Using media to impact health policy-making: an integrative systematic review | Bou-Karroum, Lama; El-Jardali, Fadi; Hemadi, Nour; Faraj, Yasmine; Ojha, Utkarsh; Shahrour, Maher; Darzi, Andrea; Ali, Maha; Doumit, Carine; Langlois, Etienne V.; Melki, Jad; AbouHaidar, Gladys Honein; Akl, Elie A. | 2017 | Implementation Science | Exclusion reason: Wrong intervention; |
| Boulet 2008 | Improving knowledge transfer on chronic respiratory diseases: a Canadian perspective. How to translate recent advances in respiratory diseases into day-to-day care | Boulet, L. P. | 2008 | Journal of Nutrition, Health & Aging | Exclusion reason: Wrong intervention; |
| Bowden 2006 | Health information Hispanic outreach in the Texas Lower Rio Grande Valley | Bowden, Virginia M.; Wood, Frederick B.; Warner, Debra G.; Olney, Cynthia A.; Olivier, Evelyn R.; Siegel, Elliot R. | 2006 | Journal of the Medical Library Association | Exclusion reason: Wrong intervention; |
| Bowen 2005 | Demystifying knowledge translation: learning from the community | Bowen, S.; Martens, P.; Need to Know, Team | 2005 | Journal of Health Services & Research Policy | Exclusion reason: Wrong intervention; |
| Bowen 2019 | Experience of Health Leadership in Partnering With University-Based Researchers in Canada â€“ A Call to â€œRe-imagineâ€ Research | Bowen, Sarah; Botting, Ingrid; Graham, Ian D.; MacLeod, Martha; de Moissac, Danielle; Harlos, Karen; Leduc, Bernard; Ulrich, Catherine; Knox, Janet | 2019 | International Journal of Health Policy & Management | Exclusion reason: Wrong intervention; |
| Brantnell 2015 | Research funders' roles and perceived responsibilities in relation to the implementation of clinical research results: a multiple case study of Swedish research funders | Brantnell, Anders; Baraldi, Enrico; van Achterberg, Theo; Winblad, Ulrika | 2015 | Implementation Science | Exclusion reason: Wrong intervention; |
| Brawley 2007 | [Physical activity guidelines for Canadians: strategies for dissemination of the message, expectations for change and evaluation] | Brawley, L. R.; Latimer, A. E. | 2007 | Applied Physiology, Nutrition, & Metabolism = Physiologie Appliquee, Nutrition et Metabolisme | Exclusion reason: Duplicate; |
| Brawley 2007 | Physical activity guides for Canadians: messaging strategies, realistic expectations for change, and evaluation | Brawley, L. R.; Latimer, A. E. | 2007 | Canadian Journal of Public Health. Revue Canadienne de Sante Publique | Exclusion reason: Wrong study design; |
| Bredin 2013 | Physical Activity Line: effective knowledge translation of evidence-based best practice in the real-world setting | Bredin, S. S.; Warburton, D. E. | 2013 | Canadian Family Physician | Exclusion reason: Wrong study design; |
| Brennan 2016 | Design and formative evaluation of the Policy Liaison Initiative: a long-term knowledge translation strategy to encourage and support the use of Cochrane systematic reviews for informing health policy | Brennan, Sue E.; Cumpston, Miranda; Misso, Marie L.; McDonald, Steve; Murphy, Matthew J.; Green, Sally E. | 2016 | Evidence & Policy: A Journal of Research, Debate & Practice | Exclusion reason: Wrong population; |
| Breslau 2015 | The implementation road: engaging community partnerships in evidence-based cancer control interventions | Breslau, E. S.; Weiss, E. S.; Williams, A.; Burness, A.; Kepka, D. | 2015 | Health Promotion Practice | Exclusion reason: Wrong intervention; |
| Briand 2020 | Benefits of a Recovery-Oriented Knowledge Translation Program for Mental Health Community Support Teams: A Qualitative Study | Briand, Catherine; Routhier, Danielle; Hakin, RÃ©gis; Vachon, Brigitte; St-Paul, Rose-Anne; Gilbert, Michel | 2020 | Canadian Journal of Community Mental Health | Exclusion reason: Wrong intervention; |
| Brimmer 2018 | Evaluation of myalgic encephalomyelitis/chronic fatigue syndrome (ME/CFS) education materials in local health departments | Brimmer, Dana J.; Hagaman, Ashley; Campbell, Charlotte; Hsu, Joanne; Lin, Jin-Mann S. | 2018 | Fatigue: Biomedicine, Health & Behavior | Exclusion reason: Wrong intervention; |
| Broner 2001 | Knowledge transfer, policymaking and community empowerment: a consensus model approach for providing public mental health and substance abuse services | Broner, N.; Franczak, M.; Dye, C.; McAllister, W. | 2001 | Psychiatric Quarterly | Exclusion reason: Wrong study design; |
| Brooke-Sumner 2019 | 'Doing more with less': a qualitative investigation of perceptions of South African health service managers on implementation of health innovations | Brooke-Sumner, Carrie; Petersen-Williams, Petal; Kruger, James; Mahomed, Hassan; Myers, Bronwyn | 2019 | Health Policy & Planning | Exclusion reason: Wrong outcomes; |
| Brooy 2017 | The research-policy-deliberation nexus: a case study approach | Brooy, Camille La; Kelaher, Margaret; La Brooy, Camille | 2017 | Health Research Policy & Systems | Exclusion reason: Wrong intervention; |
| Brownson 2006 | Translating scientific discoveries into public health action: how can schools of public health move us forward? | Brownson, R. C.; Kreuter, M. W.; Arrington, B. A.; True, W. R. | 2006 | Public Health Reports | Exclusion reason: Wrong study design; |
| Brownson 2007 | The effect of disseminating evidence-based interventions that promote physical activity to health departments | Brownson, R. C.; Ballew, P.; Brown, K. L.; Elliott, M. B.; Haire-Joshu, D.; Heath, G. W.; Kreuter, M. W. | 2007 | American Journal of Public Health | Exclusion reason: Wrong intervention; |
| Brownson 2009 | Bridging the gap: translating research into policy and practice | Brownson, R. C.; Jones, E. | 2009 | Preventive Medicine | Exclusion reason: Wrong study design; |
| Brownson 2018 | Getting the Word Out: New Approaches for Disseminating Public Health Science | Brownson, R. C.; Eyler, A. A.; Harris, J. K.; Moore, J. B.; Tabak, R. G. | 2018 | Journal of Public Health Management & Practice | Exclusion reason: Wrong study design; |
| Bruen 2020 | "We're not there to protect ourselves, we're there to talk about workforce planning": A qualitative study of policy dialogues as a mechanism to inform medical workforce planning | Bruen, Carlos; Brugha, Ruairi | 2020 | Health Policy | Exclusion reason: Wrong intervention; |
| Buller 2012 | Enhancing industry-based dissemination of an occupational sun protection program with theory-based strategies employing personal contact | Buller, D. B.; Andersen, P. A.; Walkosz, B. J.; Scott, M. D.; Cutter, G. R.; Dignan, M. B.; Kane, I. L.; Zhang, X. | 2012 | American Journal of Health Promotion | Exclusion reason: Wrong intervention; |
| Buscemi 2014 | Adaptation and dissemination of an evidence-based obesity prevention intervention: design of a comparative effectiveness trial | Buscemi, J.; Odoms-Young, A.; Stolley, M. L.; Blumstein, L.; Schiffer, L.; Berbaum, M. L.; McCaffrey, J.; Montoya, A. M.; Braunschweig, C.; Fitzgibbon, M. L. | 2014 | Contemporary Clinical Trials | Exclusion reason: Wrong study design; |
| Businger 2015 | Observing and Forecasting Vog Dispersion from KÄ«lauea Volcano, Hawaii | Businger, Steven; Huff, Roy; Pattantyus, Andre; Horton, Keith; Sutton, A. Jeff; Elias, Tamar; Cherubini, Tiziana | 2015 | Bulletin of the American Meteorological Society | Exclusion reason: Wrong outcomes; |
| Buskamp 2021 | [Promotion of physical activity among the elderly as an example of knowledge translation: How do scientific findings enter community practice?] | Buskamp, A.; Vonstein, C.; Tillmann, J.; Rosmann, C.; De Bock, F. | 2021 | Bundesgesundheitsblatt, Gesundheitsforschung, Gesundheitsschutz | Exclusion reason: Full text not available in English; |
| Buttriss 2011 | Translating complex science into life-course health promoting strategies | Buttriss, J. L. | 2011 | Proceedings of the Nutrition Society | Exclusion reason: Wrong population; |
| Caburnay 2001 | Disseminating effective health promotion programs from prevention research to community organizations | Caburnay, C. A.; Kreuter, M. W.; Donlin, M. J. | 2001 | Journal of Public Health Management & Practice | Exclusion reason: Wrong intervention; |
| Callister 2019 | Tweeting the Headache Meetings: Cross-Sectional Analysis of Twitter Activity Surrounding American Headache Society Conferences | Callister, M. N.; Robbins, M. S.; Callister, N. R.; Vargas, B. B. | 2019 | Headache | Exclusion reason: Wrong intervention; |
| Campbell 2006 | Public education on hypertension: a new initiative to improve the prevention, treatment and control of hypertension in Canada | Campbell, N. R.; Petrella, R.; Kaczorowski, J. | 2006 | Canadian Journal of Cardiology | Exclusion reason: Wrong study design; |
| Campbell 2014 | Taking knowledge for health the extra mile: participatory evaluation of a mobile phone intervention for community health workers in Malawi | Campbell, N.; Schiffer, E.; Buxbaum, A.; McLean, E.; Perry, C.; Sullivan, T. M. | 2014 | Global Health Science & Practice | Exclusion reason: Wrong intervention; |
| Campbell 2014 | Adult protection training for community nurses: evaluating knowledge following delivery using participant-favoured training methods | Campbell, Martin | 2014 | Journal of Adult Protection | Exclusion reason: Wrong intervention; |
| Campbell 2017 | Assessing healthcare professional knowledge, attitudes, and practices on hypertension management. Announcing a new World Hypertension League resource | Campbell, N. R. C.; Dashdorj, N.; Baatarsuren, U.; Myanganbayar, M.; Dashtseren, M.; Unurjargal, T.; Zhang, X. H.; Veiga, E. V.; Beheiry, H. M.; Mohan, S.; Almustafa, B.; Niebylski, M.; Lackland, D. | 2017 | Journal of Clinical Hypertension | Exclusion reason: Wrong study design; |
| Campbell 2018 | Practical strategies and perceptions from community pharmacists following their experiences with conducting pharmacy practice research: a qualitative content analysis | Campbell, Natasha K. J.; De Vera, Mary A.; Galo, Jessica S.; Chhina, Harpreet; Marra, Carlo | 2018 | International Journal of Pharmacy Practice | Exclusion reason: Wrong intervention; |
| CasajuanaKogel 2014 | [Evaluation of the Health Observatory of Asturias (Spain): web and social network metrics and health professionals' opinions] | Casajuana Kogel, C.; Cofino, R.; Lopez, M. J. | 2014 | Gaceta Sanitaria | Exclusion reason: Full text not available in English; |
| Chambers 2011 | Maximizing the Impact of Systematic Reviews in Health Care Decision Making: A Systematic Scoping Review of Knowledge-Translation Resources | Chambers, Duncan; Wilson, Paul M.; Thompson, Carl A.; Hanbury, Andria; Farley, Katherine; Light, Kate | 2011 | Milbank Quarterly | Exclusion reason: Wrong intervention; |
| Chatfield 2013 | Translating research into practice: the introduction of the INTERGROWTH-21st package of clinical standards, tools and guidelines into policies, programmes and services | Chatfield, A.; Caglia, J. M.; Dhillon, S.; Hirst, J.; Cheikh Ismail, L.; Abawi, K.; Kac, G.; Al Dhaheri, A. S.; Villar, J.; Kennedy, S.; Langer, A. | 2013 | BJOG: An International Journal of Obstetrics & Gynaecology | Exclusion reason: Wrong study design; |
| Chew-Graham 2014 | Aiming to improve the quality of primary mental health care: developing an intervention for underserved communities | Chew-Graham, Carolyn; Burroughs, Heather; Hibbert, Derek; Gask, Linda; Beatty, Susan; Gravenhorst, Katja; Waheed, Waquas; KovandÅ¾iÄ‡, Marija; Gabbay, Mark; Dowrick, Chris | 2014 | BMC Family Practice | Exclusion reason: Wrong intervention; |
| Choi 2004 | Speaker's corner: Two information dissemination approaches for public health decision makers: encyclopaedia and fire alarm | Choi, Bernard C. K.; Orlova, Anna; Marsh, Marsha; Issa, Nabil; Morrison, Howard | 2004 | Journal of Epidemiology & Community Health | Exclusion reason: Wrong study design; |
| Choi 2016 | Bridging the gap between science and policy: an international survey of scientists and policy makers in China and Canada | Choi, B. C.; Li, L.; Lu, Y.; Zhang, L. R.; Zhu, Y.; Pak, A. W.; Chen, Y.; Little, J. | 2016 | Implementation Science | Exclusion reason: Wrong intervention; |
| Clark 2013 | Evidence and obesity prevention: developing evidence summaries to support decision making | Clark, Rachel; Waters, Elizabeth; Armstrong, Rebecca; Conning, Rebecca; Allender, Steven; Swinburn, Boyd | 2013 | Evidence & Policy: A Journal of Research, Debate & Practice | Exclusion reason: Duplicate; |
| Clark 2013 | Evidence and obesity prevention: Developing evidence summaries to support decision makng | Clark, Rachel; Waters, Elizabeth; Armstrong, Rebecca; Conning, Rebecca; Allender, Steven; Swinburn, Boyd | 2013 | Evidence and Policy | Exclusion reason: Wrong study design; |
| Clifford 2009 | Disseminating best-evidence health-care to Indigenous health-care settings and programs in Australia: identifying the gaps | Clifford, A.; Pulver, L. Jackson; Richmond, R.; Shakeshaft, A.; Ivers, R. | 2009 | Health Promotion International | Exclusion reason: Wrong outcomes; |
| Close 2010 | Barriers to the Adoption and Implementation of Preventive Dental Services in Primary Medical Care | Close, Kelly; Rozier, R. Gary; Zeldin, Leslie P.; Gilbert, Allison R. | 2010 | Pediatrics | Exclusion reason: Wrong intervention; |
| Collie 2016 | Academic perspectives and experiences of knowledge translation: A qualitative study of public health researchers | Collie, Alex; Zardo, Pauline; McKenzie, Donna Margaret; Ellis, Niki | 2016 | Evidence and Policy | Exclusion reason: Wrong intervention; |
| Colquhoun 2016 | Evaluation of a training program for medicines-oriented policymakers to use a database of systematic reviews | Colquhoun, Heather L.; Lowe, Dianne; Helis, Eftyhia; Belanger, Denis; Ens, Brendalynn; Hill, Sophie; Mayhew, Alain; Taylor, Michael; Grimshaw, Jeremy M. | 2016 | Health Research Policy & Systems | Exclusion reason: Wrong intervention; |
| Conklin 2013 | Knowledge brokers in a knowledge network: the case of Seniors Health Research Transfer Network knowledge brokers | Conklin, James; Lusk, Elizabeth; Harris, Megan; Stolee, Paul | 2013 | Implementation Science | Exclusion reason: Wrong outcomes; |
| Conn 2010 | Data-driven. HHS unleashes wave of public health data so software developers can create tools to raise awareness, spur action | Conn, J. | 2010 | Modern Healthcare | Exclusion reason: Wrong study design; |
| Connell 2003 | The Development of an Alzheimer's Disease Channel for the Michigan Interactive Health Kiosk Project | Connell, Cathleen M.; Shaw, Benjamin A.; Holmes, Sara B.; Hudson, Margaret L.; Derry, Holly A.; Strecher, Victor J. | 2003 | Journal of Health Communication | Exclusion reason: Wrong population; |
| Crick 2015 | Preferences of Knowledge Users for Two Formats of Summarizing Results from Systematic Reviews: Infographics and Critical Appraisals | Crick, Katelynn; Hartling, Lisa | 2015 | PLoS ONE [Electronic Resource] | Exclusion reason: Wrong population; |
| Croker 2004 | Delivering prostate cancer prevention messages to the public: How the National Cancer Institute (NCI) effectively spread the word about the Prostate Cancer Prevention Trial (PCPT) results | Croker, Kara Smigel; Ryan, Anne; Morzenti, Thuy; Cave, Lynn; Maze-Gallman, Tamara; Ford, Leslie | 2004 | Urologic Oncology | Exclusion reason: Wrong population; |
| Cross 2010 | Predicting dissemination of a disaster mental health "Train-the-Trainer" program | Cross, W.; Cerulli, C.; Richards, H.; He, H.; Herrmann, J. | 2010 | Disaster Medicine & Public Health Preparedness | Exclusion reason: Wrong intervention; |
| Crutzen 2008 | Internet-delivered interventions aimed at adolescents: a Delphi study on dissemination and exposure | Crutzen, R.; de Nooijer, J.; Brouwer, W.; Oenema, A.; Brug, J.; de Vries, N. K. | 2008 | Health Education Research | Exclusion reason: Wrong population; |
| Curry 2011 | Academic detailing to increase colorectal cancer screening by primary care practices in Appalachian Pennsylvania | Curry, W. J.; Lengerich, E. J.; Kluhsman, B. C.; Graybill, M. A.; Liao, J. Z.; Schaefer, E. W.; Spleen, A. M.; Dignan, M. B. | 2011 | BMC Health Services Research | Exclusion reason: Wrong intervention; |
| Curtis 2018 | Factors influencing application of behavioural science evidence by public health decision-makers and practitioners, and implications for practice | Curtis, K.; Fulton, E.; Brown, K. | 2018 | Preventive Medicine Reports | Exclusion reason: Wrong intervention; |
| D'Adamo 2012 | Meeting the Health Information Needs of Health Workers: What Have We Learned? | D'Adamo, Margaret; Fabic, MadeleineShort; Ohkubo, Saori | 2012 | Journal of Health Communication | Exclusion reason: Wrong study design; |
| Dagenais 2013 | Knowledge transfer on complex social interventions in public health: a scoping study | Dagenais, C.; Malo, M.; Robert, E.; Ouimet, M.; Berthelette, D.; Ridde, V. | 2013 | PLoS ONE [Electronic Resource] | Exclusion reason: Wrong outcomes; |
| Dagenais 2016 | A Knowledge Brokering Program in Burkina Faso (West Africa): Reflections from Our Experience | Dagenais, C.; McSween-Cadieux, E.; Some, P. A.; Ridde, V. | 2016 | Health Systems & Reform | Exclusion reason: Wrong study design; |
| Dakhesh 2018 | Knowledge Translation Process among Academic Researchers: A Case Study of Bushehr University of Medical Sciences | Dakhesh, Sara; Ostovar, Afshin; Hamidi, Ali; Yazdizadeh, Bahareh | 2018 | Libri: International Journal of Libraries & Information Services | Exclusion reason: Wrong population; |
| daSilvaCorrÃªaDias 2015 | EstratÃ©gias para estimular o uso de evidÃªncias cientÃ­ficas na tomada de decisÃ£o | da Silva CorrÃªa Dias, Raphael Igor; Maia Barreto, Jorge OtÃ¡vio; Vanni, Tazio; Costa Candido, Ana Maria Silveira; Hentzy Moraes, Luciana; Rodrigues Gomes, Maria Augusta | 2015 | Encouraging the use of scientific evidence in decision making. | Exclusion reason: Full text not available in English; |
| deGoede 2010 | Knowledge in process? Exploring barriers between epidemiological research and local health policy development | de Goede, Joyce; Putters, Kim; van der Grinten, Tom; van Oers, Hans A. M.; van Oers, Hans Am | 2010 | Health Research Policy & Systems | Exclusion reason: Wrong study design; |
| Dent 2014 | Information-sharing to improve learning about community-based management of acute malnutrition (CMAM) and its impact | Dent, N.; Deconinck, H.; Golden, K.; Brown, R.; Walsh, A. | 2014 | Food & Nutrition Bulletin | Exclusion reason: Wrong intervention; |
| Derananian 2012 | Perceived versus actual factors associated with adoption and maintenance of an evidence-based physical activity program | Derananian, C. A.; Desai, P.; Smith-Ray, R.; Seymour, R. B.; Hughes, S. L. | 2012 | Translational Behavioral Medicine | Exclusion reason: Wrong outcomes; |
| Deschesnes 2010 | How divergent conceptions among health and education stakeholders influence the dissemination of healthy schools in Quebec | Deschesnes, M.; Couturier, Y.; Laberge, S.; Campeau, L. | 2010 | Health Promotion International | Exclusion reason: Wrong study design; |
| Deshpande 2015 | Comparing the Influence of Dynamic and Static Versions of Media in Evaluating Physical-Activity-Promotion Ads | Deshpande, Sameer; Berry, Tanya R.; Faulkner, Guy E. J.; Latimer-Cheung, Amy E.; Rhodes, Ryan E.; Tremblay, Mark S. | 2015 | Social Marketing Quarterly | Exclusion reason: Wrong population; |
| deSousaMata 2021 | Training in communication skills for self-efficacy of health professionals: a systematic review | de Sousa Mata, Ãdala Nayana; de Azevedo, Kesley Pablo Morais; Braga, Liliane Pereira; de Medeiros, Gidyenne Christine Bandeira Silva; de Oliveira Segundo, Victor Hugo; Bezerra, Isaac Newton Machado; Pimenta, Isac Davidson Santiago Fernandes; NicolÃ¡s, Ismael Martinez; Piuvezam, Grasiela | 2021 | Human Resources for Health | Exclusion reason: Wrong intervention; |
| Dhanasekaran 2020 | Capacity Building of Gynecologists in Cancer Screening Through Hybrid Training Approach | Dhanasekaran, K.; Babu, R.; Kumar, V.; Mehrotra, R.; Hariprasad, R. | 2020 | Journal of Cancer Education | Exclusion reason: Wrong intervention; |
| Diamond 2011 | The Development of Building Wellnessâ„¢, a Youth Health Literacy Program | Diamond, Catherine; Saintonge, Sandy; August, Phyllis; Azrack, Adeline | 2011 | Journal of Health Communication | Exclusion reason: Wrong population; |
| Dobbins 2001 | Factors of the innovation, organization, environment, and individual that predict the influence five systematic reviews had on public health decisions | Dobbins, M.; Cockerill, R.; Barnsley, J.; Ciliska, D. | 2001 | Int J Technol Assess Health Care | Exclusion reason: Wrong outcomes; |
| Dobbins 2009 | A description of a knowledge broker role implemented as part of a randomized controlled trial evaluating three knowledge translation strategies | Dobbins, M.; Robeson, P.; Ciliska, D.; Hanna, S.; Cameron, R.; O'Mara, L.; DeCorby, K.; Mercer, S. | 2009 | Implementation Science | Exclusion reason: Wrong outcomes; |
| Dorman 2011 | A Skin Cancer Prevention and Early Detection Program Disseminated Through Cosmetologists Using Evidence-Based Curriculum: Talkin' About Better Skin (TABS) | Dorman, Melody; Wernicke, Meghan; Wood, Richard; PeÃ±a-Purcell, Ninfa; Ory, Marcia | 2011 | Texas Public Health Journal | Exclusion reason: Wrong intervention; |
| Dubois 2020 | Canada's National Collaborating Centres: Facilitating evidence-informed decision-making in public health | Dubois, A.; Levesque, M. | 2020 | Canada Communicable Disease Report | Exclusion reason: Wrong study design; |
| Duggan 2019 | Disseminating Cultural Neuropsychology Research: Five Key Recommendations for Skill Development | Duggan, E. | 2019 | Archives of Clinical Neuropsychology | Exclusion reason: Wrong study design; |
| Dumitrescu 2005 | Knowledge transfer in the WHO Regional Office for Europe: the Health Evidence Network | Dumitrescu, Anca | 2005 | European Journal of Public Health | Exclusion reason: Wrong study design; |
| Duncan 2008 | Knowledge translation: Empowering health professionals to take the lead | Duncan, Carol; Langlais, S. U. E.; Danyluk-Hall, Joanne; Simonson, Kari | 2008 | Journal of Continuing Education in the Health Professions | Exclusion reason: Wrong population; |
| Dykeman 2014 | Pre-implementation knowledge tool development for health services providers: A qualitative study of Canadian social workers | Dykeman, Sarah; Williams, Allison; Crooks, Valorie | 2014 | Qualitative Social Work | Exclusion reason: Wrong intervention; |
| Dzewaltowski 2004 | The future of physical activity behavior change research: what is needed to improve translation of research into health promotion practice? | Dzewaltowski, D. A.; Estabrooks, P. A.; Glasgow, R. E. | 2004 | Exercise & Sport Sciences Reviews | Exclusion reason: Wrong study design; |
| Early 2019 | Use of Mobile Health (mHealth) Technologies and Interventions Among Community Health Workers Globally: A Scoping Review | Early, Jody; Gonzalez, Carmen; Gordon-Dseagu, Vanessa; Robles-Calderon, Laura | 2019 | Health Promotion Practice | Exclusion reason: Wrong intervention; |
| Economos 2019 | Dissemination of healthy kids out of school principles for obesity prevention: A RE-AIM analysis | Economos, C. D.; Anzman-Frasca, S.; Koomas, A. H.; Bakun, P. J.; Brown, C. M.; Brown, D.; Folta, S. C.; Fullerton, K. J.; Sacheck, J. M.; Sharma, S.; Nelson, M. E. | 2019 | Preventive Medicine | Exclusion reason: Wrong intervention; |
| Edwards 2015 | Online Continuing Education for Expanding Clinicians' Roles in Breastfeeding Support | Edwards, R. A.; Colchamiro, R.; Tolan, E.; Browne, S.; Foley, M.; Jenkins, L.; Mainello, K.; Vallu, R.; Hanley, L. E.; Boisvert, M. E.; Forgit, J.; Ghiringhelli, K.; Nordstrom, C. | 2015 | Journal of Human Lactation | Exclusion reason: Wrong intervention; |
| Edwards 2019 | Evidence map of knowledge translation strategies, outcomes, facilitators and barriers in African health systems | Edwards, Amanda; Olivier, Jill; Zweigenthal, Virginia | 2019 | Health Research Policy & Systems | Exclusion reason: Wrong intervention; |
| Einarson 2006 | Do we have a knowledge transfer and translation plan at Teratogen Information Services? | Einarson, A.; Lockett, D. | 2006 | Reproductive Toxicology | Exclusion reason: Wrong study design; |
| Eldredge 2008 | The effect of training on question formulation among public health practitioners: results from a randomized controlled trial | Eldredge, J. D.; Carr, R.; Broudy, D.; Voorhees, R. E. | 2008 | Journal of the Medical Library Association | Exclusion reason: Wrong intervention; |
| El-Jardali 2012 | Use of health systems and policy research evidence in the health policymaking in eastern Mediterranean countries: views and practices of researchers | El-Jardali, Fadi; Lavis, John N.; Ataya, Nour; Jamal, Diana | 2012 | Implementation Science | Exclusion reason: Wrong intervention; |
| Ellen 2011 | Determining research knowledge infrastructure for healthcare systems: a qualitative study | Ellen, Moriah E.; Lavis, John N.; Ouimet, Mathieu; Grimshaw, Jeremy; BÃ©dard, Pierre-Olivier | 2011 | Implementation Science | Exclusion reason: Wrong study design; |
| Ellen 2014 | Health systems and policy research evidence in health policy making in Israel: what are researchers' practices in transferring knowledge to policy makers? | Ellen, Moriah E.; Lavis, John N.; Sharon, Assaf; Shemer, Joshua | 2014 | Health Research Policy & Systems | Exclusion reason: Wrong population; |
| Ellen 2018 | How is the use of research evidence in health policy perceived? A comparison between the reporting of researchers and policy-makers | Ellen, Moriah E.; Lavis, John N.; Horowitz, Einav; Berglas, Robin | 2018 | Health Research Policy & Systems | Exclusion reason: Wrong population; |
| Erickson 2015 | Translation of obesity practice guidelines: measurement and evaluation | Erickson, K. J.; Monsen, K. A.; Attleson, I. S.; Radosevich, D. M.; Oftedahl, G.; Neely, C.; Thorson, D. R. | 2015 | Public Health Nursing | Exclusion reason: Wrong intervention; |
| Eriksson 2011 | Newborn care and knowledge translationperceptions among primary healthcare staff in northern Vietnam | Eriksson, Leif; Nguyen Thu, Nga; Hoa, Dinh P.; Persson, Lars-Ã…ke; Ewald, Uwe; Wallin, Lars | 2011 | Implementation Science | Exclusion reason: Wrong intervention; |
| Erismann 2021 | How to bring research evidence into policy? Synthesizing strategies of five research projects in low-and middle-income countries | Erismann, SÃ©verine; Pesantes, Maria Amalia; Beran, David; Leuenberger, Andrea; Farnham, Andrea; Berger Gonzalez de White, Monica; Labhardt, Niklaus Daniel; Tediosi, Fabrizio; Akweongo, Patricia; Kuwawenaruwa, August; Zinsstag, Jakob; Brugger, Fritz; Somerville, Claire; Wyss, Kaspar; Prytherch, Helen | 2021 | Health Research Policy & Systems | Exclusion reason: Wrong intervention; |
| Eschbach 2013 | Knowledge and Translation Core - Principal Investigators | Eschbach, Karl; DiNuzzo, Anthony; Zwelling, Leonard | 2013 | Texas Public Health Journal | Exclusion reason: Wrong intervention; |
| Fabrizio 2014 | Disseminating a cervical cancer screening program through primary physicians in Hong Kong: a qualitative study | Fabrizio, C. S.; Shea, C. M. | 2014 | BMC Health Services Research | Exclusion reason: Wrong intervention; |
| Farewell 2020 | An exploration of constructs related to dissemination and implementation of an early childhood systems-level intervention | Farewell, C. V.; Puma, J.; Bergling, E.; Webb, J.; Quinlan, J.; Shah, P.; Maiurro, E. | 2020 | Health Education Research | Exclusion reason: Wrong intervention; |
| Farkas 2003 | Knowledge dissemination and utilization in gerontology: An organizing framework | Farkas, Marianne; Jette, Alan M.; Tennstedt, Sharon; Haley, Stephen M.; Quinn, Virginia | 2003 | The Gerontologist | Exclusion reason: Wrong intervention; |
| Faulkner 2018 | ParticipACTION after 5 years of relaunch: a quantitative survey of Canadian organizational awareness and capacity regarding physical activity initiatives | Faulkner, G.; Ramanathan, S.; Plotnikoff, R. C.; Berry, T.; Deshpande, S.; Latimer-Cheung, A. E.; Rhodes, R. E.; Tremblay, M. S.; Spence, J. C. | 2018 | Health Promotion and Chronic Disease Prevention in Canada | Exclusion reason: Wrong intervention; |
| Ferlie 2003 | Novel mode of knowledge production? Producers and consumers in health services research | Ferlie, Ewan; Wood, Martin | 2003 | Journal of Health Services Research & Policy | Exclusion reason: Wrong population; |
| Fernandez 2019 | Promoting meal planning through mass media: awareness of a nutrition campaign among Canadian parents | Fernandez, M. A.; Desroches, S.; Marquis, M.; Lebel, A.; Turcotte, M.; Provencher, V. | 2019 | Public Health Nutrition | Exclusion reason: Wrong population; |
| Field 2019 | Accessible continued professional development for maternal mental health | Field, Sally; Abrahams, Zulfa; Woods, David L.; Turner, Roseanne; Onah, Michael N.; Kaura, Doreen K.; Honikman, Simone | 2019 | African Journal of Primary Health Care & Family Medicine | Exclusion reason: Wrong intervention; |
| Fillon 2012 | Dermatologists start skin cancer awareness initiative | Fillon, M. | 2012 | Journal of the National Cancer Institute | Exclusion reason: Wrong population; |
| Finlay 2005 | Physical activity promotion through the mass media: inception, production, transmission and consumption | Finlay, S. J.; Faulkner, G. | 2005 | Preventive Medicine | Exclusion reason: Wrong population; |
| Finnell 2018 | A Social Marketing Approach to 1% Milk Use: Resonance Is the Key | Finnell, K. J.; John, R. | 2018 | Health Promotion Practice | Exclusion reason: Wrong population; |
| Fitzgerald 2003 | Innovation in healthcare: how does credible evidence influence professionals? | Fitzgerald, Louise; Ferlie, Ewan; Hawkins, Christine | 2003 | Health & Social Care in the Community | Exclusion reason: Wrong intervention; |
| Fleischhacker 2012 | Tools for healthy tribes: improving access to healthy foods in Indian country | Fleischhacker, S.; Byrd, R. R.; Ramachandran, G.; Vu, M.; Ries, A.; Bell, R. A.; Evenson, K. R. | 2012 | American Journal of Preventive Medicine | Exclusion reason: Wrong outcomes; |
| Fleuren 2015 | A systematic approach to implementing and evaluating clinical guidelines: The results of fifteen years of preventive child health care guidelines in the Netherlands | Fleuren, Margot A.; van Dommelen, Paula; Dunnink, Trudy | 2015 | Social Science & Medicine | Exclusion reason: Wrong intervention; |
| Formoso 2007 | Social marketing: should it be used to promote evidence-based health information? | Formoso, G.; Marata, A. M.; Magrini, N. | 2007 | Social Science & Medicine | Exclusion reason: Wrong study design; |
| Fretheim 2006 | Improving the use of research evidence in guideline development: 15. Disseminating and implementing guidelines | Fretheim, Atle; SchÃ¼nemann, Holger J.; Oxman, Andrew D. | 2006 | Health Research Policy & Systems | Exclusion reason: Wrong intervention; |
| Fulford 2020 | Service Provider Perspectives Regarding Knowledge Sharing Activities in Community-Based Services | Fulford, Casey; Cobigo, Virginie | 2020 | Canadian Journal of Community Mental Health | Exclusion reason: Wrong intervention; |
| Gabbay 2020 | Uncovering the processes of knowledge transformation: the example of local evidence-informed policy-making in United Kingdom healthcare | Gabbay, John; le May, AndrÃ©e; Pope, Catherine; Brangan, Emer; Cameron, Ailsa; Klein, Jonathan H.; Wye, Lesley | 2020 | Health Research Policy & Systems | Exclusion reason: Wrong intervention; |
| Gagliardi 2016 | Identifying the conditions needed for integrated knowledge translation (IKT) in health care organizations: qualitative interviews with researchers and research users | Gagliardi, Anna R.; Dobrow, Mark J. | 2016 | BMC Health Services Research | Exclusion reason: Wrong intervention; |
| Gagliardi 2016 | Integrated knowledge translation (IKT) in health care: a scoping review | Gagliardi, Anna R.; Berta, Whitney; Kothari, Anita; Boyko, Jennifer; Urquhart, Robin | 2016 | Implementation Science | Exclusion reason: Wrong intervention; |
| Gaines 2011 | Sharing MedlinePlusÂ®/MEDLINEÂ® for Information Literacy Education (SMILE): A Dental Public Health Information Project | Gaines, Julie K.; Levy, Linda S.; Cogdill, Keith W. | 2011 | Medical Reference Services Quarterly | Exclusion reason: Wrong intervention; |
| Gainforth 2014 | The role of interpersonal communication in the process of knowledge mobilization within a community-based organization: a network analysis | Gainforth, H. L.; Latimer-Cheung, A. E.; Athanasopoulos, P.; Moore, S.; Ginis, K. A. | 2014 | Implementation Science | Exclusion reason: Wrong population; |
| Gallagher 2012 | Health message framing effects on attitudes, intentions, and behavior: a meta-analytic review | Gallagher, K. M.; Updegraff, J. A. | 2012 | Annals of Behavioral Medicine | Exclusion reason: Wrong population; |
| Galvin 2011 | Internet-Based Dementia Resources: Physician Attitudes and Practices | Galvin, James E.; Meuser, Thomas M.; Boise, Linda; Connell, Cathleen M. | 2011 | Journal of Applied Gerontology | Exclusion reason: Wrong intervention; |
| Gardner 2010 | Clinic Consortia Media Advocacy Capacity: Partnering with the Media and Increasing Policymaker Awareness | Gardner, Annette; Geierstanger, Sara; Brindis, Claire; McConnel, Coline | 2010 | Journal of Health Communication | Exclusion reason: Wrong intervention; |
| Garzon-Orjuela 2019 | Effectiveness of knowledge translation strategies in audiovisual language compared with other languages to improve health outcomes in individuals and the general population: Systematic review | Garzon-Orjuela, Nathaly; Sanchez Bello, Nubia Fernanda; Bonilla Mahecha, Lina Paola; Moreno Hernandez, Leydy Angelica; Suarez Angel, Maria Cristina; Murcia Ardila, Natalia Valentina; Luque Angulo, Silvia Catalina; Eslava-Schmalbach, Javier | 2019 | Revista Colombiana de Psiquiatria | Exclusion reason: Wrong population; |
| Gatewood 2020 | Social Media in Public Health: Strategies to Distill, Package, and Disseminate Public Health Research | Gatewood, J.; Monks, S. L.; Singletary, C. R.; Vidrascu, E.; Moore, J. B. | 2020 | Journal of Public Health Management & Practice | Exclusion reason: Wrong study design; |
| Gavens 2019 | Processes of local alcohol policy-making in England: Does the theory of policy transfer provide useful insights into public health decision-making? | Gavens, Lucy; Holmes, John; Buykx, Penny; de Vocht, Frank; Egan, Matt; Grace, Daniel; Lock, Karen; Mooney, John D.; Brennan, Alan | 2019 | Health & Place | Exclusion reason: Wrong intervention; |
| Gentry 2019 | How can we achieve impact from public health research? A meta-ethnography of case studies | Gentry, S. V.; Milden, L.; Kelly, M. P. | 2019 | Journal of Public Health | Exclusion reason: Wrong intervention; |
| Gerrish 2011 | The role of advanced practice nurses in knowledge brokering as a means of promoting evidence-based practice among clinical nurses | Gerrish, Kate; McDonnell, Ann; Nolan, Mike; Guillaume, Louise; Kirshbaum, Marilyn; Tod, Angela | 2011 | Journal of Advanced Nursing (John Wiley & Sons, Inc.) | Exclusion reason: Wrong population; |
| Gholami 2013 | Knowledge translation in Iranian universities: need for serious interventions | Gholami, Jaleh; Ahghari, Sharareh; Motevalian, Abbas; Yousefinejad, Vahid; Moradi, Ghobad; Keshtkar, Abbasali; Alami, Ali; Mazloomzadeh, Saeideh; Masoud Vakili, Mohammad; Chaman, Reza; Salehi, Bahman; Fazelzadeh, Omid; Majdzadeh, Reza | 2013 | Health Research Policy & Systems | Exclusion reason: Wrong population; |
| Giles-Corti 2015 | Translating active living research into policy and practice: one important pathway to chronic disease prevention | Giles-Corti, B.; Sallis, J. F.; Sugiyama, T.; Frank, L. D.; Lowe, M.; Owen, N. | 2015 | Journal of Public Health Policy | Exclusion reason: Wrong study design; |
| Gill 2019 | HealthPathways improving access to care | Gill, S. D.; Mansfield, S.; McLeod, M.; von Treuer, K.; Dunn, M.; Quirk, F. | 2019 | Australian Health Review | Exclusion reason: Wrong intervention; |
| Glanz 2013 | Randomized trial of tailored skin cancer prevention for children: the Project SCAPE family study | Glanz, K.; Steffen, A. D.; Schoenfeld, E.; Tappe, K. A. | 2013 | Journal of Health Communication | Exclusion reason: Wrong population; |
| Glenton 2013 | Cochrane i Norge -- Hvordan formidler vi resultatene fra Cochrane-oversikter? | Glenton, Claire; Rosenbaum, Sarah | 2013 | Cochrane in Norway -- How do we disseminate findings from Cochrane reviews? | Exclusion reason: Full text not available in English; |
| Glowacki 2016 | Targeting Type 2: Linguistic Agency Assignment in Diabetes Prevention Policy Messaging | Glowacki, Elizabeth M.; McGlone, Matthew S.; Bell, Robert A. | 2016 | Journal of Health Communication | Exclusion reason: Wrong population; |
| Glowacki 2017 | E-Cigarette Topics Shared by Medical Professionals: A Comparison of Tweets from the United States and United Kingdom | Glowacki, Elizabeth M.; Lazard, Allison J.; Wilcox, Gary B. | 2017 | CyberPsychology, Behavior & Social Networking | Exclusion reason: Wrong intervention; |
| Godbee 2020 | Refined conceptual model for implementing dementia risk reduction: incorporating perspectives from Australian general practice | Godbee, Kali; Gunn, Jane; Lautenschlager, Nicola T.; Palmer, Victoria J. | 2020 | Australian Journal of Primary Health | Exclusion reason: Wrong outcomes; |
| Goedert 2013 | HHS Wants Input on Speeding Health Information Exchange Adoption | Goedert, Joseph | 2013 | Healthdatamanagement.com | Exclusion reason: Wrong study design; |
| Golden 2021 | A Dissemination Strategy to Identify Communities Ready to Implement a Pediatric Weight Management Intervention in Medically Underserved Areas | Golden, C. A.; Hill, J. L.; Heelan, K. A.; Bartee, R. T.; Abbey, B. M.; Malmkar, A.; Estabrooks, P. A. | 2021 | Preventing Chronic Disease | Exclusion reason: Wrong intervention; |
| Golden-Biddle 2003 | Toward a communicative perspective of collaborating in research: the case of the researcherâ€“decision-maker partnership | Golden-Biddle, Karen; Reay, Trish; Petz, Steve; Witt, Christine; Casebeer, Ann; Pablo, Amy; Hinings, C. R. | 2003 | Journal of Health Services Research & Policy | Exclusion reason: Wrong intervention; |
| Goyet 2014 | Knowledge translation: a case study on pneumonia research and clinical guidelines in a low- income country | Goyet, Sophie; Barennes, Hubert; Libourel, Therese; van Griensven, Johan; Frutos, Roger; Tarantola, Arnaud | 2014 |  | Exclusion reason: Wrong intervention; |
| Gray 2011 | Narrative and framing: a test of an integrated message strategy in the exercise context | Gray, J. B.; Harrington, N. G. | 2011 | Journal of Health Communication | Exclusion reason: Wrong population; |
| Gray 2017 | Building Competency and Capacity for Promotion of Effective Physical Activity in Diabetes Care in Canada | Gray, E.; Shields, C.; Fowles, J. R. | 2017 | Canadian Journal of Diabetes | Exclusion reason: Wrong intervention; |
| Grayson 2020 | Developing pathways for community-led research with big data: a content analysis of stakeholder interviews | Grayson, Shira; Doerr, Megan; Yu, Joon-Ho | 2020 | Health Research Policy & Systems | Exclusion reason: Wrong intervention; |
| Green 2006 | Inferring strategies for disseminating physical activity policies, programs, and practices from the successes of tobacco control | Green, L. W.; Orleans, C. T.; Ottoson, J. M.; Cameron, R.; Pierce, J. P.; Bettinghaus, E. P. | 2006 | American Journal of Preventive Medicine | Exclusion reason: Wrong study design; |
| Grimshaw 2012 | Knowledge translation of research findings | Grimshaw, Jeremy M.; Eccles, Martin P.; Lavis, John N.; Hill, Sophie J.; Squires, Janet E. | 2012 | Implementation Science | Exclusion reason: Wrong study design; |
| Guell 2017 | Negotiating multisectoral evidence: a qualitative study of knowledge exchange at the intersection of transport and public health | Guell, Cornelia; Mackett, Roger; Ogilvie, David | 2017 | BMC Public Health | Exclusion reason: Wrong intervention; |
| HÃ¤mÃ¤lÃ¤inen 2015 | Exploring the use of research evidence in health-enhancing physical activity policies | HÃ¤mÃ¤lÃ¤inen, Riitta-Maija; Aro, Arja R.; van de Goor, Ien; Juel Lau, Cathrine; Jakobsen, Mette Winge; Chereches, Razvan M.; Syed, Ahmed M.; Lau, Cathrine Juel; Repopa Consortium | 2015 | Health Research Policy & Systems | Exclusion reason: Wrong intervention; |
| Haalboom 2006 | Research as intervention in heart health promotion | Haalboom, B. J.; Robinson, K. L.; Elliott, S. J.; Cameron, R.; Eyles, J. D. | 2006 | Canadian Journal of Public Health. Revue Canadienne de Sante Publique | Exclusion reason: Wrong intervention; |
| Hagell 2004 | An evaluation of an innovative audiotape method for keeping social care staff up to date with the latest research findings | Hagell, Ann; Spencer, Liz | 2004 | Child & Family Social Work | Exclusion reason: Wrong population; |
| Hambrick 2014 | Towards Successful Dissemination of Psychological First Aid: A Study of Provider Training Preferences | Hambrick, Erin; Rubens, Sonia; Vernberg, Eric; Jacobs, Anne; Kanine, Rebecca | 2014 | Journal of Behavioral Health Services & Research | Exclusion reason: Wrong intervention; |
| Hansen 2009 | Using Online Components to Facilitate Program Implementation: Impact of Technological Enhancements to All Stars on Ease and Quality of Program Delivery | Hansen, William; Bishop, Dana; Bryant, Kelvin | 2009 | Prevention Science | Exclusion reason: Wrong intervention; |
| Harada 2011 | [Longitudinal change in awareness levels of Japanese exercise guidelines and physical activity] | Harada, K.; Shibata, A.; Lee, E.; Oka, K.; Nakamura, Y. | 2011 | Nippon Koshu Eisei Zasshi - Japanese Journal of Public Health | Exclusion reason: Full text not available in English; |
| Harden 2017 | Improving physical activity program adoption using integrated research-practice partnerships: an effectiveness-implementation trial | Harden, S. M.; Johnson, S. B.; Almeida, F. A.; Estabrooks, P. A. | 2017 | Translational Behavioral Medicine | Exclusion reason: Wrong intervention; |
| Hariprasad 2018 | Retention of Knowledge Levels of Health Care Providers in Cancer Screening Through Telementoring | Hariprasad, R.; Arora, S.; Babu, R.; Sriram, L.; Sardana, S.; Hanumappa, S.; Mehrotra, R. | 2018 | Journal of Global Oncology | Exclusion reason: Wrong intervention; |
| Harris 2012 | A framework for disseminating evidence-based health promotion practices | Harris, J. R.; Cheadle, A.; Hannon, P. A.; Forehand, M.; Lichiello, P.; Mahoney, E.; Snyder, S.; Yarrow, J. | 2012 | Preventing Chronic Disease | Exclusion reason: Wrong intervention; |
| Harris 2013 | The network of web 2.0 connections among state health departments: new pathways for dissemination | Harris, J. K. | 2013 | Journal of Public Health Management & Practice | Exclusion reason: Wrong intervention; |
| Harris 2014 | Are public health organizations tweeting to the choir? Understanding local health department Twitter followership | Harris, J. K.; Choucair, B.; Maier, R. C.; Jolani, N.; Bernhardt, J. M. | 2014 | Journal of Medical Internet Research | Exclusion reason: Wrong outcomes; |
| Harris 2016 | Screening, brief intervention, and referral to treatment for adolescents: Attitudes, perceptions, and practice of New York school-based health center providers | Harris, Brett R.; Shaw, Benjamin A.; Sherman, Barry R.; Lawson, Hal A. | 2016 | Substance Abuse | Exclusion reason: Wrong intervention; |
| Harrop 2012 | Translating cancer prevention and control research into the community setting: workforce implications | Harrop, J. P.; Nelson, D. E.; Kuratani, D. G.; Mullen, P. D.; Paskett, E. D. | 2012 | Journal of Cancer Education | Exclusion reason: Wrong study design; |
| Harvey 2019 | Mobilising evidence to improve nursing practice: A qualitative study of leadership roles and processes in four countries | Harvey, Gill; Gifford, Wendy; Cummings, Greta; Kelly, Janet; Kislov, Roman; Kitson, Alison; Pettersson, Lena; Wallin, Lars; Wilson, Paul; Ehrenberg, Anna | 2019 | International Journal of Nursing Studies | Exclusion reason: Wrong population; |
| Hasan 2012 | Diabetologist's perspective on practice of evidence based diabetes management in India | Hasan, Habib; Zodpey, Sanjay; Saraf, Abhay | 2012 | Diabetes Research & Clinical Practice | Exclusion reason: Wrong intervention; |
| Haynes 2018 | A cross-Canada knowledge transfer and exchange workplace intervention targeting the adoption of sun safety programs and practices: Sun Safety at Work Canada | Haynes, Emily; Kramer, Desre M.; Strahlendorf, Peter; Holness, D. Linn; Kushner, Rivka; Tenkate, Thomas | 2018 | Safety Science | Exclusion reason: Wrong intervention; |
| Heinrich 2017 | How to initiate dementia care networks? Processes, barriers, and facilitators during the development process of a practice-oriented website toolkit out of research results | Heinrich, Steffen; Sommerfeld, Ulrike; Michalowsky, Bernhard; Hoffmann, Wolfgang; Thyrian, Jochen Rene; Wolf-Ostermann, Karin; Roes, Martina | 2017 | International Quarterly of Community Health Education | Exclusion reason: Wrong intervention; |
| Henderson 2006 | Closing the Research-Practice Gap: Factors Affecting Adoption and Implementation of a Children's Mental Health Program | Henderson, Joanna L.; MacKay, Sherri; Peterson-Badali, Michele | 2006 | Journal of Clinical Child & Adolescent Psychology | Exclusion reason: Wrong intervention; |
| Henderson 2015 | Program manager perspectives on the service system to meet the needs of youth with concurrent disorders: findings from a Canadian national survey | Henderson, Joanna L.; Chaim, Gloria; Luca, Stephanie; Brownlie, E. B.; Rosenkranz, Susan; Skilling, Tracey A.; Beitchman, Joseph H. | 2015 | BMC Health Services Research | Exclusion reason: Wrong population; |
| Hendriks 2014 | Predicting Health: The Interplay Between Interpersonal Communication and Health Campaigns | Hendriks, Hanneke; van den Putte, Bas; de Bruijn, Gert-Jan; de Vreese, ClaesH | 2014 | Journal of Health Communication | Exclusion reason: Wrong population; |
| Hennink 2005 | Using Research to Inform Health Policy: Barriers and Strategies in Developing Countries | Hennink, Monique; Stephenson, R. O. B. | 2005 | Journal of Health Communication | Exclusion reason: Wrong population; |
| Herie 2000 | Attitudes Toward Substance Abuse Treatment Among Probation and Parole Officers | Herie, Marilyn; Cunningham, John; Martin, Garth W. | 2000 | Journal of Offender Rehabilitation | Exclusion reason: Wrong intervention; |
| Herie 2012 | Changing practitioner behavior and building capacity in tobacco cessation treatment: The TEACH project | Herie, Marilyn; Connolly, Hillary; Voci, Sabrina; Dragonetti, Rosa; Selby, Peter | 2012 | Patient Education & Counseling | Exclusion reason: Wrong intervention; |
| Hermes 2019 | Beliefs and Attitudes About the Dissemination and Implementation of Internet-Based Self-Care Programs in a Large Integrated Healthcare System | Hermes, Eric D. A.; Burrone, Laura; Heapy, Alicia; Martino, Steve; Perez, Elliottnell; Rosenheck, Robert; Rowe, Michael; Ruzek, Josef I.; Greene, Carolyn | 2019 | Administration & Policy in Mental Health & Mental Health Services Research | Exclusion reason: Wrong intervention; |
| HernÃ¡ndez-Aguado 2020 | The role of the media in the health policymaking process: perspectives of key actors in Spain | HernÃ¡ndez-Aguado, Ildefonso; Chilet-Rosell, Elisa | 2020 | Critical Public Health | Exclusion reason: Wrong population; |
| Hernandez 2020 | What do health providers and patients tweet about schizophrenia? | Hernandez, M. Y.; Hernandez, M.; Lopez, D. H.; Gamez, D.; Lopez, S. R. | 2020 | Early intervention in psychiatry | Exclusion reason: Wrong outcomes; |
| Hinton 2012 | Evaluation of a Culturally Adapted Training in Indigenous Mental Health and Wellbeing for the Alcohol and Other Drug Workforce | Hinton, Rachael; Nagel, Tricia | 2012 | Isrn Public Healthonline | Exclusion reason: Wrong intervention; |
| Hobden 2006 | Barriers to the dissemination of four harm reduction strategies: a survey of addiction treatment providers in Ontario | Hobden, Karen L.; Cunningham, John A. | 2006 | Harm Reduction Journal | Exclusion reason: Wrong intervention; |
| Hobin 2012 | Maximising the use of evidence: exploring the intersection between population health intervention research and knowledge translation from a Canadian perspective | Hobin, Erin P.; Hayward, Sarah; Riley, Barbara; Di Ruggiero, Erica; Birdsell, Judy | 2012 | Evidence & Policy: A Journal of Research, Debate & Practice | Exclusion reason: Wrong intervention; |
| Hoeijmakers 2013 | Academic Collaborative Centre Limburg: a platform for knowledge transfer and exchange in public health policy, research and practice? | Hoeijmakers, M.; Harting, J.; Jansen, M. | 2013 | Health Policy | Exclusion reason: Wrong outcomes; |
| Hoek 2010 | Lessons from New Zealand's introduction of pictorial health warnings on tobacco packaging | Hoek, J.; Wilson, N.; Allen, M.; Edwards, R.; Thomson, G.; Li, J. | 2010 | Bulletin of the World Health Organization | Exclusion reason: Wrong intervention; |
| Honeycutt 2012 | Research to reality: a process evaluation of a mini-grants program to disseminate evidence-based nutrition programs to rural churches and worksites | Honeycutt, S.; Carvalho, M.; Glanz, K.; Daniel, S. D.; Kegler, M. C. | 2012 | Journal of Public Health Management & Practice | Exclusion reason: Wrong intervention; |
| Honeycutt 2015 | Evaluating Policy, Systems, and Environmental Change Interventions: Lessons Learned From CDC's Prevention Research Centers | Honeycutt, S.; Leeman, J.; McCarthy, W. J.; Bastani, R.; Carter-Edwards, L.; Clark, H.; Garney, W.; Gustat, J.; Hites, L.; Nothwehr, F.; Kegler, M. | 2015 | Preventing Chronic Disease | Exclusion reason: Wrong outcomes; |
| Honeycutt 2017 | Practice to Evidence: Using Evaluability Assessment to Generate Practice-Based Evidence in Rural South Georgia | Honeycutt, S.; Hermstad, A.; Carvalho, M. L.; Arriola, K. R. J.; Ballard, D.; Escoffery, C.; Kegler, M. C. | 2017 | Health Education & Behavior | Exclusion reason: Wrong intervention; |
| Hong 2020 | Influence of Presumed Media Influence for Health Prevention: How Mass Media Indirectly Promote Health Prevention Behaviors through Descriptive Norms | Hong, Yangsun; Kim, Sunghak | 2020 | Health Communication | Exclusion reason: Wrong population; |
| Hoogland 2017 | WAT LEVERT EEN TRAINING VOOR VOEDSELBANKKLANTEN OP? BEVINDINGEN UIT EEN KWALITATIEVE STUDIE | Hoogland, Hille; Mul, Dorien | 2017 | What are the results of a training programme for food bank clients? Findings from a qualitative study. | Exclusion reason: Full text not available in English; |
| Hoover 2018 | Enhancing Smoking Risk Communications: The Influence of Health Literacy and Message Content | Hoover, Diana Stewart; Wetter, David W.; Vidrine, Damon J.; Nguyen, Nga; Frank, Summer G.; Li, Yisheng; Waters, Andrew J.; Meade, Cathy D.; Vidrine, Jennifer I. | 2018 | Annals of Behavioral Medicine | Exclusion reason: Wrong population; |
| Hople 2011 | Food based dietary guidelines in Vietnam: progress and lessons learned | Hop le, T.; Van, T. K.; Thanh, H. K. | 2011 | Asia Pacific Journal of Clinical Nutrition | Exclusion reason: Wrong study design; |
| Horn 2014 | Oral health promotion and education messages in Live.Learn.Laugh. projects | Horn, V.; Phantumvanit, P. | 2014 | International Dental Journal | Exclusion reason: Wrong population; |
| Huber 2020 | Social Media Research Strategy to Understand Clinician and Public Perception of Health Care Messages | Huber, J.; Woods, T.; Fushi, A.; Duong, M. T.; Eidelman, A. S.; Zalal, A. R.; Urquhart, O.; Colangelo, E.; Quinn, S.; Carrasco-Labra, A. | 2020 | Jdr Clinical & Translational Research | Exclusion reason: Wrong intervention; |
| Hudson 2013 | Value of social media in reaching and engaging employers in Total Worker Health | Hudson, H.; Hall, J. | 2013 | Journal of Occupational & Environmental Medicine | Exclusion reason: Wrong intervention; |
| Hugo 2018 | InforMD: a new initiative to raise public awareness about breast density | Hugo, Honor J.; Zysk, Aneta; Dasari, Pallave; Britt, Kara; Hopper, John L.; Stone, Jennifer; Thompson, Erik W.; Ingman, Wendy V. | 2018 | Ecancermedicalscience | Exclusion reason: Wrong population; |
| Hung 2018 | A meta-analysis of the evaluations of social marketing interventions addressing smoking, alcohol drinking, physical activity, and eating | Hung, Chia-Ling | 2018 | Dissertation Abstracts International: Section B: The Sciences and Engineering | Exclusion reason: Wrong population; |
| Hunt 2020 | An integrated knowledge translation project to develop, implement, and evaluate a train-the-trainer program at a community rehabilitation program in Tamil Nadu, India | Hunt, Matthew; Ponnusamy, Ramasubramanian; Goulet, Anik; Anthonypillai, Chamila; Muthukaruppan, Sankar Sahayaraj; Bharathwaj, Aravind; Thomas, Aliki; Archambault, Philippe S.; Garnett, Crystal; Storr, Caroline; Krishna, Dinesh | 2020 | Disability & Rehabilitation | Exclusion reason: Wrong population; |
| Icard 2003 | Designing social marketing strategies to increase African Americans' access to health promotion programs | Icard, L. D.; Bourjolly, J. N.; Siddiqui, N. | 2003 | Health & Social Work | Exclusion reason: Wrong population; |
| Ilic 2013 | What is the evidence that poster presentations are effective in promoting knowledge transfer? A state of the art review | Ilic, D.; Rowe, N. | 2013 | Health Information & Libraries Journal | Exclusion reason: Wrong intervention; |
| Instituteof 2013 | | Institute of, Medicine | 2013 | National Academies Press | Exclusion reason: Wrong intervention; |
| Ir 2010 | Translating knowledge into policy and action to promote health equity: The Health Equity Fund policy process in Cambodia 2000-2008 | Ir, P.; Bigdeli, M.; Meessen, B.; Van Damme, W. | 2010 | Health Policy | Exclusion reason: Wrong intervention; |
| IrishHauser 2010 | Comparison of Online and Face-to-Face Dissemination of a Theory-Based After School Nutrition and Physical Activity Training and Curriculum | Irish Hauser, Sonya; Goldberg, JeanneP; Wilde, Parke; Bers, Marina; Ioannone, Lori; Economos, ChristinaD | 2010 | Journal of Health Communication | Exclusion reason: Wrong intervention; |
| Jacobs 2010 | Barriers to evidence-based decision making in public health: a national survey of chronic disease practitioners | Jacobs, J. A.; Dodson, E. A.; Baker, E. A.; Deshpande, A. D.; Brownson, R. C. | 2010 | Public Health Reports | Exclusion reason: Wrong intervention; |
| Jacobson 2005 | Consulting as a Strategy for Knowledge Transfer | Jacobson, Nora; Butterill, Dale; Goering, Paula | 2005 | Milbank Quarterly | Exclusion reason: Wrong intervention; |
| Jessani 2021 | Integrated knowledge translation to advance noncommunicable disease policy and practice in South Africa: application of the Exploration, Preparation, Implementation, and Sustainment (EPIS) framework | Jessani, N. S.; Rohwer, A.; Schmidt, B. M.; Delobelle, P. | 2021 | Health Research Policy & Systems | Exclusion reason: Wrong intervention; |
| Johnstone 2006 | System-wide adoption of health promotion practices by schools: evaluation of a telephone and mail-based dissemination strategy in Australia | Johnstone, E.; Knight, J.; Gillham, K.; Campbell, E.; Nicholas, C.; Wiggers, J. | 2006 | Health Promotion International | Exclusion reason: Wrong intervention; |
| Jolliffe 2020 | Stroke rehabilitation research translation in Australia: a survey of clinical trialists | Jolliffe, Laura; Hoffmann, Tammy; Laver, Kate; McCluskey, Annie; Lannin, Natasha A. | 2020 | Disability & Rehabilitation | Exclusion reason: Wrong population; |
| Jones 2012 | It's all in the lens: differences in views on obesity prevention between advocates and policy makers | Jones, E.; Eyler, A. A.; Nguyen, L.; Kong, J.; Brownson, R. C.; Bailey, J. H. | 2012 | Childhood Obesity | Exclusion reason: Wrong study design; |
| Jones 2015 | Knowledge Translation for researchers: developing training to support public health researchers KTE efforts | Jones, K.; Armstrong, R.; Pettman, T.; Waters, E. | 2015 | Journal of Public Health | Exclusion reason: Wrong intervention; |
| Jones 2015 | Interventions to reach underscreened populations: A narrative review for planning cancer screening initiatives | Jones, Mavis; Ross, Brenda; Cloth, Alyssa; Heller, Laura | 2015 | International Journal of Public Health | Exclusion reason: Wrong population; |
| Jordans 2014 | New frontiers in mental health and psychosocial wellbeing in low resource and conflict affected settings | Jordans, Mark J. D.; Tol, Wietse A.; Ventevogel, Peter | 2014 | Intervention (15718883) | Exclusion reason: Wrong intervention; |
| JudyHuei-yu 2018 | Physician Intervention and Chinese Americans' Colorectal Cancer Screening | Judy Huei-yu, Wang; Ma, Grace X.; Wenchi, Liang; Yin, Tan; Makambi, Kepher H.; Roucheng, Dong; Vernon, Sally W.; Shin-Ping, Tu; Mandelblatt, Jeanne S. | 2018 | American Journal of Health Behavior | Exclusion reason: Wrong outcomes; |
| Kaftarian 2000 | Bridging the gap between research and practice in community-based substance abuse prevention | Kaftarian, Shakeh Jackie; Wandersman, Abraham | 2000 | Journal of Community Psychology | Exclusion reason: Wrong study design; |
| Kalef 2016 | Employers' Perspectives on the Canadian National Standard for Psychological Health and Safety in the Workplace | Kalef, Laura; Rubin, Courtney; Malachowski, Cindy; Kirsh, Bonnie | 2016 | Employee Responsibilities & Rights Journal | Exclusion reason: Wrong intervention; |
| Kameg 2020 | Technology-Based Educational Approaches to Address Opioid Use Management by Advanced Practice Registered Nurses | Kameg, Brayden N.; Mitchell, Ann | 2020 | Issues in Mental Health Nursing | Exclusion reason: Wrong intervention; |
| Kandula 2012 | A Community and Culture-Centered Approach to Developing Effective Cardiovascular Health Messages | Kandula, Namratha; Khurana, Neerja; Makoul, Gregory; Glass, Sara; Baker, David | 2012 | JGIM: Journal of General Internal Medicine | Exclusion reason: Wrong population; |
| Karacabeyli 2020 | The Live 5-2-1-0 Toolkit for family physicians: Mixed methods evaluation of a resource to facilitate health promotion in a primary care setting | Karacabeyli, Derin; Shea, Stephanie; Keidar, Shelly; Pinkney, Susan; Bepple, Katrina; Edwards, Danielle; Hale, Ilona; Suleman, Selina; Amed, Shazhan | 2020 | British Columbia Medical Journal | Exclusion reason: Wrong intervention; |
| Karlin 2020 | Improving engagement in evidence-based psychological treatments among Veterans: Direct-to-consumer outreach and pretreatment shared decision-making | Karlin, Bradley E.; Brenner, Lisa A. | 2020 | Clinical Psychology: Science and Practice Vol 27(4), 2020, ArtID e12344 | Exclusion reason: Wrong population; |
| KaroliinaEnwald 2010 | Preventing the obesity epidemic by second generation tailored health communication: An interdisciplinary review | Karoliina Enwald, Heidi Paivyt; Aulikki Huotari, Maija-Leena | 2010 | Journal of Medical Internet Research | Exclusion reason: Wrong population; |
| Katz 2018 | Tourette syndrome: The impact of training on psychologists' knowledge, efficacy and diagnostic accuracy and the status of scholarly dissemination | Katz, Nicole G. | 2018 | Dissertation Abstracts International: Section B: The Sciences and Engineering | Exclusion reason: Wrong intervention; |
| Keddem 2020 | The Gears of Knowledge Translation: Process Evaluation of the Dissemination and Implementation of a Patient Engagement Toolkit | Keddem, Shimrit; Agha, Aneeza Z.; Long, Judith A.; Shasha, Becky; Hausmann, Leslie R. M.; Shea, Judy A. | 2020 | JGIM: Journal of General Internal Medicine | Exclusion reason: Wrong population; |
| Kennedy 2015 | Osteoporosis Prescribing in Long-Term Care: Impact of a Provincial Knowledge Translation Strategy | Kennedy, Courtney C.; Ioannidis, George; Thabane, Lehana; Adachi, Jonathan D.; Oâ€™Donnell, Denis; Giangregorio, Lora M.; Pickard, Laura E.; Papaioannou, Alexandra | 2015 | Canadian Journal on Aging | Exclusion reason: Wrong population; |
| Kennedy 2017 | Process evaluation of a preschool physical activity intervention using web-based delivery | Kennedy, A. B.; Schenkelberg, M.; Moyer, C.; Pate, R.; Saunders, R. P. | 2017 | Evaluation & Program Planning | Exclusion reason: Wrong intervention; |
| King 2011 | Best practice principles for community-based obesity prevention: development, content and application | King, L.; Gill, T.; Allender, S.; Swinburn, B. | 2011 | Obesity Reviews | Exclusion reason: Wrong study design; |
| Kinley 2004 | Changing practice: use of audit to change oral care practice | Kinley, Julie; Brennan, Sonya | 2004 | International Journal of Palliative Nursing | Exclusion reason: Wrong intervention; |
| Kirkeby 2015 | Designing for health in school buildings: between research and practice | Kirkeby, I. M.; Jensen, B. B.; Larsen, K.; Kural, R. | 2015 | Scandinavian Journal of Public Health | Exclusion reason: Wrong outcomes; |
| Kirkpatrick 2014 | Florida Red Tide Knowledge and Risk Perception: Is there a need for tailored messaging? | Kirkpatrick, B.; Kohler, K.; Byrne, M. M.; Studts, J. | 2014 | Harmful Algae | Exclusion reason: Wrong population; |
| Kirkpatrick 2021 | The impact of source and message relevance on audience responses to health podcasts | Kirkpatrick, Ciera E.; Lee, Sungkyoung | 2021 | Communication Reports | Exclusion reason: Wrong population; |
| Kislov 2014 | BOUNDARY DISCONTINUITY IN A CONSTELLATION OF INTERCONNECTED PRACTICES | Kislov, Roman | 2014 |  | Exclusion reason: Wrong population; |
| Kite 2016 | Please Like Me: Facebook and Public Health Communication | Kite, J.; Foley, B. C.; Grunseit, A. C.; Freeman, B. | 2016 | PLoS ONE [Electronic Resource] | Exclusion reason: Wrong population; |
| Kliche 2012 | [Knowledge transfer methods in German disease prevention and health promotion. A survey of experts in the federal prevention research program] | Kliche, T.; Post, M.; Pfitzner, R.; Plaumann, M.; Dubben, S.; Nocker, G.; Walter, U. | 2012 | Gesundheitswesen | Exclusion reason: Full text not available in English; |
| Ko 2019 | An application of the Science Impact Framework to the Cancer Prevention and Control Research Network from 2014-2018 | Ko, L. K.; Jang, S. H.; Friedman, D. B.; Glanz, K.; Leeman, J.; Hannon, P. A.; Shannon, J.; Cole, A.; Williams, R.; Vu, T. | 2019 | Preventive Medicine | Exclusion reason: Wrong intervention; |
| Kobus 2007 | Communications strategies to broaden the reach of tobacco use research: examples from the Transdisciplinary Tobacco Use Research Centers | Kobus, Kimberly; Mermelstein, Robin; Ponkshe, Prabhu | 2007 | Nicotine & Tobacco Research | Exclusion reason: Wrong study design; |
| Kohr 2008 | The use of programme planning and social marketing models by a state public health agency: A case study | Kohr, J. M.; Strack, R. W.; Newton-Ward, M.; Cooke, C. H. | 2008 |  | Exclusion reason: Wrong intervention; |
| Kondo 2009 | Folic acid in the prevention of neural tube defects: awareness among laywomen and healthcare providers in Japan | Kondo, A.; Yamamoto, S.; Inoue, H.; Watanabe, J.; Tada, K.; Yoshimoto, N. | 2009 | Congenital Anomalies | Exclusion reason: Wrong intervention; |
| Kong 2010 | Addressing disparities in sudden cardiac arrest care and the underutilization of effective therapies | Kong, M. H.; Peterson, E. D.; Fonarow, G. C.; Sanders, G. D.; Yancy, C. W.; Russo, A. M.; Curtis, A. B.; Sears, S. F., Jr.; Thomas, K. L.; Campbell, S.; Carlson, M. D.; Chiames, C.; Cook, N. L.; Hayes, D. L.; LaRue, M.; Hernandez, A. F.; Lyons, E. L.; Al-Khatib, S. M. | 2010 | American Heart Journal | Exclusion reason: Wrong study design; |
| Kothari 2009 | Increasing capacity for knowledge translation: understanding how some researchers engage policy makers | Kothari, Anita; MacLean, Lynne; Edwards, Nancy | 2009 | Evidence & Policy: A Journal of Research, Debate & Practice | Exclusion reason: Wrong population; |
| Koumpouros 2015 | The importance of patient engagement and the use of Social Media marketing in healthcare | Koumpouros, Yiannis; Toulias, Thomas L.; Koumpouros, Nicholas | 2015 | Technology & Health Care | Exclusion reason: Wrong population; |
| Kraft 2012 | Active Living by Design Sustainability Strategies | Kraft, M. Katherine; Lee, Joanne J.; Brennan, Laura K. | 2012 | American Journal of Preventive Medicine | Exclusion reason: Wrong intervention; |
| Kramer 2015 | It takes a state: Best practices for children exposed to trauma | Kramer, Teresa L.; Sigel, Benjamin A.; Conners-Burrow, Nicola; Worley, Karen B.; Church, Jan K.; Helpenstill, Kathy | 2015 | Best Practices in Mental Health: An International Journal | Exclusion reason: Wrong intervention; |
| Kramer 2017 | Sun Safety at Work Canada: Baseline evaluation of outdoor workplaces recruited to participate in a sun safety knowledge transfer and exchange intervention | Kramer, Desre M.; Haynes, Emily; Holness, D. Linn; Strahlendorf, Peter; Kushner, Rivka; Tenkate, Thomas | 2017 | Safety Science | Exclusion reason: Wrong intervention; |
| Kreps 2005 | Disseminating relevant health information to underserved audiences: implications of the Digital Divide Pilot Projects | Kreps, G. L. | 2005 | Journal of the Medical Library Association | Exclusion reason: Wrong outcomes; |
| Kreps 2005 | Narrowing the Digital Divide to Overcome Disparities in Care | Kreps, Gary L. | 2005 | Health communication in practice: A case study approach | Exclusion reason: Wrong study design; |
| Kreps 2007 | Future directions for the cancer information service and cancer education | Kreps, G. L.; Bright, M. A.; Fleisher, L.; Marcus, A.; Morra, M. E.; Perocchia, R. S. | 2007 | Journal of Cancer Education | Exclusion reason: Wrong study design; |
| Kreps 2012 | Health communication inquiry and health outcomes | Kreps, Gary L. | 2012 | ComunicaÃ§Ã£o e Sociedade | Exclusion reason: Wrong study design; |
| Kreps 2012 | Translating Health Communication Research Into Practice: The Importance of Implementing and Sustaining Evidence-Based Health Communication Interventions | Kreps, GaryL | 2012 | Atlantic Journal of Communication | Exclusion reason: Wrong study design; |
| Kwan 2007 | A systematic evaluation of payback of publicly funded health and health services research in Hong Kong | Kwan, Patrick; Johnston, Janice; Fung, Anne Y. K.; Chong, Doris S. Y.; Collins, Richard A.; Lo, Su V. | 2007 | BMC Health Services Research | Exclusion reason: Wrong intervention; |
| Kyoon-Achan 2021 | Early childhood oral health promotion for First Nations and Metis communities and caregivers in Manitoba | Kyoon-Achan, G.; Schroth, R. J.; Sanguins, J.; Campbell, R.; Demare, D.; Sturym, M.; Edwards, J.; Bertone, M.; Dufour, L.; Hai Santiago, K.; Chartrand, F.; Dhaliwal, T.; Patterson, B.; Levesque, J.; Moffatt, M.; Scaling Up the Healthy Smile Happy Child, Team | 2021 | Health Promotion and Chronic Disease Prevention in Canada | Exclusion reason: Wrong population; |
| LÃ¤hteenmÃ¤ki 2004 | Consumers and Health: Getting the Probiotic Message Across | LÃ¤hteenmÃ¤ki, Liisa | 2004 | Microbial Ecology in Health & Disease | Exclusion reason: Wrong population; |
| LÃ©garÃ© 2017 | Responsiveness of a simple tool for assessing change in behavioral intention after continuing professional development activities | LÃ©garÃ©, France; Freitas, Adriana; Turcotte, StÃ©phane; Borduas, Francine; Jacques, AndrÃ©; Luconi, Francesca; Godin, Gaston; Boucher, AndrÃ©e; Sargeant, Joan; Labrecque, Michel | 2017 | PLoS ONE [Electronic Resource] | Exclusion reason: Wrong outcomes; |
| Laird 2020 | Stakeholders' experiences of the public health research process: time to change the system? | Laird, Y.; Manner, J.; Baldwin, L.; Hunter, R.; McAteer, J.; Rodgers, S.; Williamson, C.; Jepson, R. | 2020 | Health Research Policy & Systems | Exclusion reason: Wrong intervention; |
| Laird 2021 | Recognition and Management of Protracted Bacterial Bronchitis in Australian Aboriginal Children: A Knowledge Translation Approach | Laird, Pam; Walker, Roz; Lane, Mary; Totterdell, James; Chang, Anne B.; Schultz, AndrÃ© | 2021 | Chest | Exclusion reason: Wrong intervention; |
| Lalonde 2007 | Media training for diabetes prevention: a participatory evaluation | Lalonde, J.; Jeambey, Z.; Starkey, L. J. | 2007 | Canadian Journal of Dietetic Practice & Research | Exclusion reason: Wrong intervention; |
| Lamb 2018 | Evaluation of Entertainment Education Strategies to Promote Cervical Cancer Screening and Knowledge in Colombian Women | Lamb, R. L. B.; Ramos Jaraba, S. M.; Graciano Tangarife, V.; Garces-Palacio, I. C. | 2018 | Journal of Cancer Education | Exclusion reason: Wrong population; |
| Lambert 2018 | Exploring the impact of efficacy messages on cessation-related outcomes using Ecological Momentary Assessment | Lambert, Victoria; Ferguson, Stuart G.; Niederdeppe, Jeff; Hammond, David; Hardin, James W.; Thrasher, James F. | 2018 | Tobacco Induced Diseases | Exclusion reason: Wrong population; |
| Lane 2012 | Applying the interactive systems framework to the dissemination and adoption of national and state recommendations for hypertension | Lane, R. I.; Berkowitz, J. M.; Sullivan, S. T.; Rose, J.; Bernichon, T.; Favoretto, A.; Shifflett, P.; Miles, E.; Jones, M. | 2012 | American Journal of Community Psychology | Exclusion reason: Wrong intervention; |
| Larme 2001 | Evidence-based guidelines meet the real world: the case of diabetes care | Larme, A. C.; Pugh, J. A.; Larme, A. C.; Pugh, J. A. | 2001 | Diabetes Care | Exclusion reason: Wrong intervention; |
| Laroche 2011 | Transfer activities among Canadian researchers: Evidence in occupational safety and health | Laroche, Elena; Amara, Nabil | 2011 | Safety Science | Exclusion reason: Wrong intervention; |
| Larsen 2011 | [Good experiences with collaboration between research and practice in public health] | Larsen, M.; Gulis, G.; Bak, C. K.; Andersen, P. T.; Aro, A. R. | 2011 | Ugeskrift for Laeger | Exclusion reason: Full text not available in English; |
| Larsen 2012 | Use of evidence in local public health work in Denmark | Larsen, M.; Gulis, G.; Pedersen, K. M. | 2012 | International Journal of Public Health | Exclusion reason: Wrong outcomes; |
| Larsson 2015 | The Montana Radon Study: social marketing via digital signage technology for reaching families in the waiting room | Larsson, L. S. | 2015 | American Journal of Public Health | Exclusion reason: Wrong population; |
| Latimer 2005 | Motivating cancer prevention and early detection behaviors using psychologically tailored messages | Latimer, A. E.; Katulak, N. A.; Mowad, L.; Salovey, P. | 2005 | Journal of Health Communication | Exclusion reason: Wrong population; |
| Latimer-Cheung 2013 | Evidence-informed recommendations for constructing and disseminating messages supplementing the new Canadian Physical Activity Guidelines | Latimer-Cheung, Amy E.; Rhodes, Ryan E.; Kho, Michelle E.; Tomasone, Jennifer R.; Gainforth, Heather L.; Kowalski, Kristina; Nasuti, Gabriella; Perrier, Marie-JosÃ©e; Duggan, Mary; Canadian Physical Activity Guidelines Messaging Recommendation, Workgroup | 2013 | BMC Public Health | Exclusion reason: Wrong study design; |
| Latimer-Cheung 2016 | The Canadian 24-Hour Movement Guidelines for Children and Youth: Implications for practitioners, professionals, and organizations | Latimer-Cheung, A. E.; Copeland, J. L.; Fowles, J.; Zehr, L.; Duggan, M.; Tremblay, M. S. | 2016 | Applied Physiology, Nutrition, & Metabolism = Physiologie Appliquee, Nutrition et Metabolisme | Exclusion reason: Wrong study design; |
| Lawrence 2019 | Integrated Knowledge Translation with Public Health Policy Makers: A Scoping Review | Lawrence, L. M.; Bishop, A.; Curran, J. | 2019 | Healthcare Policy = Politiques de sante | Exclusion reason: Wrong intervention; |
| Laycock 2018 | Application of the i-PARIHS framework for enhancing understanding of interactive dissemination to achieve wide-scale improvement in Indigenous primary healthcare | Laycock, Alison; Cunningham, Frances; Harvey, Gillian; Percival, Nikki; Bailie, Jodie; Matthews, Veronica; Bailie, Ross; Copley, Kerry; Patel, Louise | 2018 | Health Research Policy & Systems | Exclusion reason: Wrong intervention; |
| Lazard 2019 | Website Designs for Communicating About Chemicals in Cigarette Smoke | Lazard, Allison J.; Byron, M. Justin; Vu, Huyen; Peters, Ellen; Schmidt, Annie; Brewer, Noel T. | 2019 | Health Communication | Exclusion reason: Wrong population; |
| Le 2018 | CervixCheck: A Spiritually-Based Text Messaging Intervention to Promote Cervical Cancer Awareness and Pap Test Screening Intention among African-American Women | Le, Daisy; Holt, Cheryl L. | 2018 | Journal of Health Communication | Exclusion reason: Wrong population; |
| LeCraw 2021 | How U.S. teams advanced communication and resolution program adoption at local, state and national levels | LeCraw, Florence R.; Stearns, Sally C.; McCoy, Michael J. | 2021 | Journal of Patient Safety & Risk Management | Exclusion reason: Wrong intervention; |
| Lederer 2009 | An Electronic Infrastructure for Research and Treatment of the Thalassemias and Other Hemoglobinopathies: The Euro-Mediterranean Ithanet Project | Lederer, Carsten W.; Basak, A. Nazli; Aydinok, Yesim; Christou, Soteroula; El-Beshlawy, Amal; Eleftheriou, Androulla; Fattoum, Slaheddine; Felice, Alex E.; Fibach, Eitan; Galanello, Renzo; Gambari, Roberto; Gavrila, Lucian; Giordano, Piero C.; Grosveld, Frank; Hassapopoulou, Helen; Hladka, Eva; Kanavakis, Emmanuel; Locatelli, Franco; Old, John; Patrinos, George P. | 2009 | Hemoglobin | Exclusion reason: Wrong intervention; |
| Lee 2010 | Dentists' Perceptions of Barriers to Providing Dental Care to Pregnant Women | Lee, Rosanna Shuk-Yin; Milgrom, Peter; Huebner, Colleen E.; Conrad, Douglas A. | 2010 | Women's Health Issues | Exclusion reason: Wrong intervention; |
| Lee 2014 | Relationships between core factors of knowledge management in hospital nursing organisations and outcomes of nursing performance | Lee, Eun Ju; Kim, Hong Soon; Kim, Hye Young | 2014 | Journal of Clinical Nursing (John Wiley & Sons, Inc.) | Exclusion reason: Wrong population; |
| Lehoux 2005 | Dissemination of Health Technology Assessments: Identifying the Visions Guiding an Evolving Policy Innovation in Canada | Lehoux, Pascale; Denis, Jean-Louis; Tailliez, StÃ©phanie; Hivon, Myriam | 2005 | Journal of Health Politics, Policy & Law | Exclusion reason: Wrong intervention; |
| Leser 2021 | Adoption, Sustainability, and Dissemination of Chronic Disease Prevention Policies in Community-Based Organizations | Leser, Kendall A.; Liu, Sherry T.; Smathers, Carol A.; Graffagnino, Cheryl L.; Pirie, Phyllis L. | 2021 | Health Promotion Practice | Exclusion reason: Wrong intervention; |
| Leshner 2009 | Scare' Em or Disgust 'Em: The Effects of Graphic Health Promotion Messages | Leshner, Glenn; Bolls, Paul; Thomas, Erika | 2009 | Health Communication | Exclusion reason: Wrong population; |
| Lester 2020 | Evaluation of the performance and achievements of the WHO Evidence-informed Policy Network (EVIPNet) Europe | Lester, Louise; Haby, Michelle M.; Chapman, Evelina; KuchenmÃ¼ller, Tanja | 2020 | Health Research Policy & Systems | Exclusion reason: Wrong intervention; |
| Leung 2010 | The trainees' perspective on developing an end-of- grant knowledge translation plan | Leung, Brenda M. Y.; Catallo, Cristina; Riediger, Natalie D.; Cahill, Naomi E.; Kastner, Monika | 2010 | Implementation Science | Exclusion reason: Wrong study design; |
| Lewis 2005 | Fluoride varnish use in primary care: what do providers think? | Lewis, C.; Lynch, H.; Richardson, L. | 2005 | Pediatrics | Exclusion reason: Wrong outcomes; |
| Leyden 2008 | Changing the hearts and minds of policy makers: an exploratory study associated with the West Virginia Walks campaign | Leyden, K. M.; Reger-Nash, B.; Bauman, A.; Bias, T. | 2008 | American Journal of Health Promotion | Exclusion reason: Wrong intervention; |
| Lim 2012 | Effects of workshop trainings on evidence-based practice knowledge and attitudes among youth community mental health providers | Lim, A.; Nakamura, B. J.; Higa-McMillan, C. K.; Shimabukuro, S.; Slavin, L. | 2012 | Behaviour Research & Therapy | Exclusion reason: Wrong intervention; |
| LimÃ³n 2016 | Training for caring complex health needs in the community: the impact of the synergy between top and bottom | LimÃ³n, Esther; Blay, Carles; Burdoy, Emili; PepiÃ³, Josep Maria; Carrillo, Ricard; Lozano, Joan | 2016 | International Journal of Integrated Care (IJIC) | Exclusion reason: Wrong study design; |
| Lineker 2019 | Getting a Grip on Arthritis Online: Responses of rural/remote primary care providers to a web-based continuing medical education programme | Lineker, Sdydney C.; Fleet, Lisa J.; Bell, Mary J.; Sweezie, Raquel; Curran, Vernon; Brock, Gordon; Badley, Elizabeth M. | 2019 | Canadian Journal of Rural Medicine (Wolters Kluwer India Pvt Ltd) | Exclusion reason: Wrong intervention; |
| Liu 2012 | Facilitating knowledge transfer: decision support tools in environment and health | Liu, H. Y.; Bartonova, A.; Neofytou, P.; Yang, A.; Kobernus, M. J.; Negrenti, E.; Housiadas, C. | 2012 | Environmental Health: A Global Access Science Source | Exclusion reason: Wrong intervention; |
| Liverani 2018 | The making of evidence-informed health policy in Cambodia: knowledge, institutions and processes | Liverani, M.; Chheng, K.; Parkhurst, J. | 2018 | BMJ Global Health | Exclusion reason: Wrong intervention; |
| Lloyd 2009 | Building capacity for evidence-based practice in the health promotion workforce: evaluation of a train-the-trainer initiative in NSW | Lloyd, B.; Rychetnik, L.; Maxwell, M.; Nove, T. | 2009 | Health Promotion Journal of Australia | Exclusion reason: Wrong intervention; |
| Lloyd 2011 | Smart use of data, information and communication: The INFORM-ed Best Local Practice Project - Grafton Base Hospital | Lloyd, Sheree; Collie, Jean; McInnes, Alastair; King, Kevin; Lollback, Alison; Garland, Angie | 2011 | Health Information Management Journal | Exclusion reason: Wrong population; |
| Loitz 2017 | Network analysis of inter-organizational relationships and policy use among active living organizations in Alberta, Canada | Loitz, C. C.; Stearns, J. A.; Fraser, S. N.; Storey, K.; Spence, J. C. | 2017 | BMC Public Health | Exclusion reason: Wrong intervention; |
| Long 2013 | Bridges, brokers and boundary spanners in collaborative networks: a systematic review | Long, Janet C.; Cunningham, Frances C.; Braithwaite, Jeffrey | 2013 | BMC Health Services Research | Exclusion reason: Wrong population; |
| Lopez 2012 | Developing the ePromotora: Increasing Promotora's Access to Breast Cancer Electronic Resources | Lopez, A. M.; Ryan, J.; Valencia, A.; El-Khayat, Y.; Nunez, A. | 2012 | Cancer Research | Exclusion reason: Wrong study design; |
| Lu 2010 | Innovative strategies to reduce disparities in the quality of prenatal care in underresourced settings | Lu, M. C.; Kotelchuck, M.; Hogan, V. K.; Johnson, K.; Reyes, C. | 2010 | Medical Care Research & Review | Exclusion reason: Wrong intervention; |
| Lubans 2016 | A school-based intervention incorporating smartphone technology to improve health-related fitness among adolescents: rationale and study protocol for the NEAT and ATLAS 2.0 cluster randomised controlled trial and dissemination study | Lubans, D. R.; Smith, J. J.; Peralta, L. R.; Plotnikoff, R. C.; Okely, A. D.; Salmon, J.; Eather, N.; Dewar, D. L.; Kennedy, S.; Lonsdale, C.; Hilland, T. A.; Estabrooks, P.; Finn, T. L.; Pollock, E.; Morgan, P. J. | 2016 | BMJ Open | Exclusion reason: Wrong study design; |
| Ludolph 2015 | Does regulatory fit lead to more effective health communication? A systematic review | Ludolph, R.; Schulz, P. J. | 2015 | Social Science & Medicine | Exclusion reason: Wrong population; |
| Lugtenberg 2016 | Occupational physicians' perceived barriers and suggested solutions to improve adherence to a guideline on mental health problems: analysis of a peer group training | Lugtenberg, Marjolein; van Beurden, Karlijn M.; Brouwers, Evelien P. M.; Terluin, Berend; van Weeghel, Jaap; van der Klink, Jac J. L.; Joosen, Margot C. W. | 2016 | BMC Health Services Research | Exclusion reason: Wrong intervention; |
| Lundkvist 2021 | Policy-makers' views on translating burden of disease estimates in health policies: bridging the gap through data visualization | Lundkvist, A.; El-Khatib, Z.; Kalra, N.; Pantoja, T.; Leach-Kemon, K.; Gapp, C.; Kuchenmuller, T. | 2021 | Archives of Public Health | Exclusion reason: Wrong intervention; |
| Ma 2008 | DIFFUSION OF PHILADELPHIA'S NO-SMOKING POLICY TO CHINESE BUSINESSES | Ma, Grace X.; Poon, Adrienne N.; Toubbeh, Jamil I. | 2008 | American Journal of Health Studies | Exclusion reason: Wrong intervention; |
| Ma 2014 | The impact of a community-based clinical trial educational intervention among underrepresented Chinese Americans | Ma, G. X.; Tan, Y.; Blakeney, N. C.; Seals, B. F.; Ma, X. S.; Zhai, S.; Liu, A.; Tai, Y.; Michaels, M. | 2014 | Cancer Epidemiology, Biomarkers & Prevention | Exclusion reason: Wrong intervention; |
| Ma 2019 | Positive Facts, Negative Stories: Message Framing as a Moderator of Narrative Persuasion in Antismoking Communication | Ma, Zexin; Nan, Xiaoli | 2019 | Health Communication | Exclusion reason: Wrong population; |
| MacDonald 2006 | A NOVEL INTERDISCIPLINARY INTENSIVE COURSE IN CANCER RISK COUNSELING AND COMMUNITY-BASED RESEARCH | MacDonald, Deborah; Blazer, Kathleen; Lowstuter, Katrina; Culver, Julie; Palomares, Melanie; Weitzel, Jeffrey | 2006 | Oncology Nursing Forum | Exclusion reason: Wrong study design; |
| MacGregor 2014 | Strategies to promote uptake and use of intimate partner violence and child maltreatment knowledge: an integrative review | MacGregor, J. C.; Wathen, N.; Kothari, A.; Hundal, P. K.; Naimi, A. | 2014 | BMC Public Health | Exclusion reason: Wrong intervention; |
| MacKinnon 2020 | Improving detection of work-related asthma: a review of gaps in awareness, reporting and knowledge translation | MacKinnon, Madison; To, Teresa; Ramsey, Clare; LemiÃ¨re, Catherine; Lougheed, M. Diane | 2020 | Allergy, Asthma & Clinical Immunology | Exclusion reason: Wrong study design; |
| Magdalinou 2019 | A Framework for Enhancing and Updating Study Programs in Public Health and Medical Informatics Fields in Montenegro | Magdalinou, A.; Mantas, J.; Sendelj, R.; Ognjanovic, I.; Knaup, P.; Ammenwerth, E.; Varga, O.; Nikolic, G.; Stojanovic, A. J.; uric, D. | 2019 | Studies in Health Technology & Informatics | Exclusion reason: Wrong intervention; |
| Maher 2016 | User-Centered Design Groups to Engage Patients and Caregivers with a Personalized Health Information Technology Tool | Maher, Molly; Kaziunas, Elizabeth; Ackerman, Mark; Derry, Holly; Forringer, Rachel; Miller, Kristen; O'Reilly, Dennis; An, Larry C.; Tewari, Muneesh; Hanauer, David A.; Choi, Sung Won | 2016 | Biology of Blood & Marrow Transplantation | Exclusion reason: Wrong intervention; |
| Maibach 2006 | A marketing perspective on disseminating evidence-based approaches to disease prevention and health promotion | Maibach, E. W.; Van Duyn, M. A.; Bloodgood, B. | 2006 | Preventing Chronic Disease | Exclusion reason: Wrong study design; |
| Maindonald 2020 | Mental health crisis training for non-mental health professionals | Maindonald, Rebecca; Attoe, Chris; Gasston-Hales, Melanie; Memon, Perah; Barley, Elizabeth | 2020 | Journal of Mental Health Training, Education & Practice | Exclusion reason: Wrong population; |
| Mairs 2013 | Online strategies to facilitate health-related knowledge transfer: a systematic search and review | Mairs, Katie; McNeil, Heather; McLeod, Jordache; Prorok, Jeanette C.; Stolee, Paul | 2013 | Health Information & Libraries Journal | Exclusion reason: Wrong intervention; |
| Majdzadeh 2008 | Knowledge translation for research utilization: Design of a knowledge translation model at Tehran University of Medical Sciences | Majdzadeh, Reza; Sadighi, Jila; Nejat, Saharnaz; Mahani, Ali Shahidzade; Gholami, Jaleh | 2008 | Journal of Continuing Education in the Health Professions | Exclusion reason: Wrong intervention; |
| Makris 2021 | A Scoping Review of Behavior Change Interventions to Decrease Health Care Disparities for Patients With Disabilities in a Primary Care Setting: Can Social Marketing Play a Role? | Makris, Angela; Khaliq, Mahmooda; Perkins, Elizabeth | 2021 | Social Marketing Quarterly | Exclusion reason: Wrong intervention; |
| Malcolm 2019 | Why, What, and How: Training Community Clinicians on Evidence-Based Practice for Youth Mental Health | Malcolm, Kenya T.; Taylor, Katelyn K.; Mitchell, Jessica; Saile, Meagan; Heatly, Melissa; Alpert-Gillis, Linda J. | 2019 | Training & Education in Professional Psychology | Exclusion reason: Wrong intervention; |
| Malekinejad 2018 | The discordance between evidence and health policy in the United States: the science of translational research and the critical role of diverse stakeholders | Malekinejad, Mohsen; Horvath, Hacsi; Snyder, Harry; Brindis, Claire D. | 2018 | Health Research Policy & Systems | Exclusion reason: Wrong study design; |
| Mammen 2019 | Shaping Core Health Messages: Rural, Low-Income Mothers Speak Through Participatory Action Research | Mammen, Sheila; Sano, Yoshie; Braun, Bonnie; Maring, Elisabeth Fost | 2019 | Health Communication | Exclusion reason: Wrong population; |
| Mamykina 2019 | P28 Qualitative Study: User Experiences with Platano, a Dietary Self-Monitoring App for Underserved Patients with Type 2 Diabetes | Mamykina, Lena; Almonte, Amenda; Sepulveda, Jhack; Heitkemper, Elizabeth; Mitchell, Elliot G.; Burgermaster, Marissa; Juul, Filippa | 2019 | Journal of Nutrition Education & Behavior | Exclusion reason: Wrong population; |
| Mamzer 2017 | Partnering with patients in translational oncology research: ethical approach | Mamzer, Marie-France; Duchange, Nathalie; Sylviane, Darquy; Marvanne, Patrice; Rambaud, Claude; Marsico, Giovanna; Cerisey, Catherine; ScottÃ©, Florian; Burgun, Anita; Badoual, CÃ©cile; Laurent-Puig, Pierre; HervÃ©, Christian; Darquy, Sylviane | 2017 | Journal of Translational Medicine | Exclusion reason: Wrong intervention; |
| Mancera-Cuevas 2018 | Addressing Lupus Health Disparities: The MONARCAS Community and Academic Collaborative Program | Mancera-Cuevas, K.; Canessa, P.; Chmiel, J. S.; Hahn, E. A.; Chung, A. H.; Erickson, D. L.; Ramsey-Goldman, R. | 2018 | Health Equity | Exclusion reason: Wrong intervention; |
| Mandal 2021 | Understanding the spread of prevention and cessation messages on social media for substance use in youth | Mandal, Soumik; Shah, Chirag; PeÃ±a-Alves, Stephanie; Hecht, Michael L.; Glenn, Shannon D.; Ray, Anne E.; Greene, Kathryn | 2021 | Aslib Journal of Information Management | Exclusion reason: Wrong population; |
| Mandel 2013 | Exploring local public health workflow in the context of automated translation technologies | Mandel, H.; Turner, A. M. | 2013 | AMIA ... Annual Symposium Proceedings/AMIA Symposium | Exclusion reason: Wrong intervention; |
| Manderscheid 2014 | Healthy People 2020: Developing the potential of mobile and digital communication tools to touch the life of every American | Manderscheid, Ron; Wukitsch, Kimber | 2014 | Journal of Communication in Healthcare | Exclusion reason: Wrong population; |
| Manno 2018 | Health risk communication message comprehension is influenced by image inclusion | Manno, Francis A. M.; Lively, Malcolm B.; Manno, Sinai H. C.; Cheng, Shuk H.; Lau, Condon; Manno, Francis A. M., 3rd | 2018 | Journal of Visual Communication in Medicine | Exclusion reason: Wrong population; |
| Mansilla 2017 | The Evidence-Informed Policy Network (EVIPNet) in Chile: lessons learned from a year of coordinated efforts | Mansilla, CristiÃ¡n; Herrera, Cristian A.; Basagoitia, Andrea; Pantoja, TomÃ¡s | 2017 | La Red de PolÃ­ticas Informadas por la Evidencia (EVIPNet) en Chile: enseÃ±anzas extraÃ­das en un aÃ±o de esfuerzos coordinados. | Exclusion reason: Wrong study design; |
| Mapa-Tassou 2018 | Two decades of tobacco use prevention and control policies in Cameroon: results from the analysis of non-communicable disease prevention policies in Africa | Mapa-Tassou, Clarisse; Bonono, Cecile RÃ©nÃ©e; Assah, Felix; Wisdom, Jennifer; Juma, Pamela A.; Katte, Jean-Claude; Njoumemi, Zakariaou; Ongolo-Zogo, Pierre; Fezeu, Leopold K.; Sobngwi, Eugene; Mbanya, Jean Claude | 2018 | BMC Public Health | Exclusion reason: Wrong intervention; |
| MarÃ­n-GonzÃ¡lez 2017 | The Role of Dissemination as a Fundamental Part of a Research Project | MarÃ­n-GonzÃ¡lez, Esther; Malmusi, Davide; CamprubÃ­, LluÃ­s; Borrell, Carme | 2017 | International Journal of Health Services | Exclusion reason: Wrong intervention; |
| Marcus 2000 | Interactive communication strategies: implications for population-based physical-activity promotion | Marcus, B. H.; Nigg, C. R.; Riebe, D.; Forsyth, L. H. | 2000 | American Journal of Preventive Medicine | Exclusion reason: Wrong study design; |
| Marcus 2007 | Step into Motion: a randomized trial examining the relative efficacy of Internet vs. print-based physical activity interventions | Marcus, B. H.; Lewis, B. A.; Williams, D. M.; Whiteley, J. A.; Albrecht, A. E.; Jakicic, J. M.; Parisi, A. F.; Hogan, J. W.; Napolitano, M. A.; Bock, B. C. | 2007 | Contemporary Clinical Trials | Exclusion reason: Wrong population; |
| Marcus 2019 | A Brochure to Improve Understanding of Incomplete Mammogram Results Among Black Women at a Public Hospital in Miami, Florida | Marcus, Erin N.; Sanders, Lee M.; Jones, Beth A.; Koru-Sengul, Tulay | 2019 | Southern Medical Journal | Exclusion reason: Wrong population; |
| Mareno 2014 | An early-phase translation study of the ways to enhance children's activity and nutrition (We Can!) programme for low-income families | Mareno, Nicole | 2014 | Journal of Clinical Nursing (John Wiley & Sons, Inc.) | Exclusion reason: Wrong population; |
| Margolis 2010 | Designing a large-scale multilevel improvement initiative: The improving performance in practice program | Margolis, Peter A.; DeWalt, Darren A.; Simon, Janet E.; Horowitz, Sheldon; Scoville, Richard; Kahn, Norman; Perelman, Robert; Bagley, Bruce; Miles, Paul | 2010 | Journal of Continuing Education in the Health Professions | Exclusion reason: Wrong intervention; |
| Markham 2016 | Internet-Based Delivery of Evidence-Based Health Promotion Programs Among American Indian and Alaska Native Youth: A Case Study | Markham, C. M.; Craig Rushing, S.; Jessen, C.; Gorman, G.; Torres, J.; Lambert, W. E.; Prokhorov, A. V.; Miller, L.; Allums-Featherston, K.; Addy, R. C.; Peskin, M. F.; Shegog, R. | 2016 | JMIR Research Protocols | Exclusion reason: Wrong population; |
| Masuda 2009 | Disseminating chronic disease prevention "to or with" Canadian public health systems | Masuda, J. R.; Robinson, K.; Elliott, S.; Eyles, J. | 2009 | Health Education & Behavior | Exclusion reason: Wrong outcomes; |
| Mavoa 2012 | Knowledge exchange in the Pacific: The TROPIC (Translational Research into Obesity Prevention Policies for Communities) project | Mavoa, Helen; Waqa, Gade; Moodie, Marj; Kremer, Peter; McCab, Marita; Snowdon, Wendy; Swinburn, Boyd | 2012 | BMC Public Health | Exclusion reason: Wrong study design; |
| Maxwell 2012 | Evaluating the Training of Filipino American Community Health Advisors to Disseminate Colorectal Cancer Screening | Maxwell, Annette; Danao, Leda; Cayetano, Reggie; Crespi, Catherine; Bastani, Roshan | 2012 | Journal of Community Health | Exclusion reason: Wrong intervention; |
| Maxwell 2014 | Adoption of an evidence-based colorectal cancer screening promotion program by community organizations serving Filipino Americans | Maxwell, A. E.; Danao, L. L.; Cayetano, R. T.; Crespi, C. M.; Bastani, R. | 2014 | BMC Public Health | Exclusion reason: Wrong intervention; |
| May 2016 | Addressing Low Colorectal Cancer Screening in African Americans: Using Focus Groups to Inform the Development of Effective Interventions | May, F. P.; Whitman, C. B.; Varlyguina, K.; Bromley, E. G.; Spiegel, B. M. | 2016 | Journal of Cancer Education | Exclusion reason: Wrong population; |
| Maynard 2016 | Inefficiency of didactic training workshops as a tool to implement addiction services in primary healthcare services | Maynard, Serge; Campbell, Emily; Boodhoo, Katie; Xenocostas, Spyrldoula; Gill, Kathryn | 2016 | Drogues, sante et societe | Exclusion reason: Full text not available in English; |
| Mbachu 2016 | Analysing key influences over actors' use of evidence in developing policies and strategies in Nigeria: a retrospective study of the Integrated Maternal Newborn and Child Health strategy | Mbachu, Chinyere O.; Onwujekwe, Obinna; Chikezie, Ifeanyi; Ezumah, Nkoli; Das, Mahua; Uzochukwu, Benjamin S. C. | 2016 | Health Research Policy & Systems | Exclusion reason: Wrong intervention; |
| McCalman 2013 | Tailoring a response to youth binge drinking in an Aboriginal Australian community: a grounded theory study | McCalman, J.; Tsey, K.; Bainbridge, R.; Shakeshaft, A.; Singleton, M.; Doran, C. | 2013 | BMC Public Health | Exclusion reason: Wrong population; |
| McCannon 2007 | The Science of Large-Scale Change in Global Health | McCannon, C. Joseph; Berwick, Donald M.; Massoud, M. Rashad | 2007 | JAMA: Journal of the American Medical Association | Exclusion reason: Wrong study design; |
| McCarthy 2013 | Impact of innovations in national public health markets in Europe | McCarthy, M.; Alexanderson, K.; Voss, M.; Conceicao, C.; Grimaud, O.; Narkauskaite, L.; Katreniakova, Z.; Saliba, A.; Sammut, M. | 2013 | European Journal of Public Health | Exclusion reason: Wrong intervention; |
| McDavid 2016 | Growing Fit: Georgia's model for engaging early care environments in preventing childhood obesity | McDavid, K.; Piedrahita, C.; Hashima, P.; Vall, E. A.; Kay, C.; O'Connor, J. | 2016 | Journal of the Georgia Public Health Association | Exclusion reason: Wrong intervention; |
| McDonald 2007 | Circle of research and practice. From evidence-based practice making to practice-based evidence making: creating communities of (research) and practice | McDonald, P. W.; Viehbeck, S. | 2007 | Health Promotion Practice | Exclusion reason: Wrong study design; |
| McLean 2018 | Translating research into action: an international study of the role of research funders | McLean, Robert K. D.; Graham, Ian D.; Tetroe, Jacqueline M.; Volmink, Jimmy A. | 2018 | Health Research Policy & Systems | Exclusion reason: Wrong population; |
| McLennan 2019 | Effects of Workshop Trainings on Practice Element Utilization among Therapists in a Youth Public Mental Health System | McLennan, Priya; Mueller, Charles W.; Heck, Ronald H.; Nakamura, Brad J. | 2019 | Evidence-Based Practice in Child & Adolescent Mental Health | Exclusion reason: Wrong intervention; |
| McLoughlin 2021 | Transdisciplinary approaches for the dissemination of the SWITCH school wellness initiative through a distributed 4-h/extension network | McLoughlin, G.; Vazou, S.; Liechty, L.; Torbert, A.; Lanningham-Foster, L.; Rosenkranz, R.; Welk, G. | 2021 | Child & Youth Care Forum | Exclusion reason: Wrong intervention; |
| McWilliam 2003 | Promoting Evidence-Based Health Policy, Programming, and Practice for Seniors: Lessons from a National Knowledge Transfer Project | McWilliam, Carol L.; Stewart, Moira; Brown, Judith Belle; Feightner, John; Rosenberg, Mark; Gutman, Gloria; Penning, Margaret; Stewart, Miriam; Tamblyn, Robyn; Morfitt, Grace | 2003 | Canadian Journal on Aging | Exclusion reason: Wrong intervention; |
| Mei-yu 2007 | Culturally Competent Training Program: A Key to Training Lay Health Advisors for Promoting Breast Cancer Screening | Mei-yu, Yu; Lixin, Song; Seetoo, Amy; Cuijuan, Cai; Smith, Gary; Oakley, Deborah | 2007 | Health Education & Behavior | Exclusion reason: Wrong intervention; |
| Merrill 2011 | Growth of a Scientific Community of Practice: Public Health Services and Systems Research | Merrill, Jacqueline A.; Keeling, Jonathan W.; Wilson, Rosalind V.; Chen, Tianle V. | 2011 | American Journal of Preventive Medicine | Exclusion reason: Wrong outcomes; |
| Michaud 2005 | Beyond the insiders' circle: disseminating the results of adolescent health surveys | Michaud, P. A.; Jeannin, A. | 2005 | Acta Paediatrica | Exclusion reason: Wrong outcomes; |
| Mickan 2011 | Patterns of 'leakage' in the utilisation of clinical guidelines: a systematic review | Mickan, S.; Burls, A.; Glasziou, P. | 2011 | Postgraduate Medical Journal | Exclusion reason: Wrong intervention; |
| Milat 2014 | Increasing the scale and adoption of population health interventions: experiences and perspectives of policy makers, practitioners, and researchers | Milat, A. J.; King, L.; Newson, R.; Wolfenden, L.; Rissel, C.; Bauman, A.; Redman, S. | 2014 | Health Research Policy & Systems | Exclusion reason: Wrong intervention; |
| Miller 2005 | Health working with industry to promote fruit and vegetables: a case study of the Western Australian Fruit and Vegetable Campaign with reflection on effectiveness of inter-sectoral action | Miller, M.; Pollard, C. | 2005 | Australian & New Zealand Journal of Public Health | Exclusion reason: Wrong population; |
| Miller 2007 | Knowledge dissemination and evaluation in a cervical cancer screening implementation program in Nigeria | Miller, Dianne; Okolo, Clement A.; Mirabal, Yvette; Guillaud, Martial; Arulogun, Oyedunni S.; Oladepo, Oladimeji; Crain, Brian; Follen, Michele; Adewole, Isaac F. | 2007 | Gynecologic Oncology | Exclusion reason: Wrong intervention; |
| Minian 2021 | The effectiveness of generic emails versus a remote knowledge broker to integrate mood management into a smoking cessation programme in team-based primary care: a cluster randomised trial | Minian, Nadia; Ahad, Sheleza; Ivanova, Anna; Veldhuizen, Scott; Zawertailo, Laurie; Ravindran, Arun; de Oliveira, Claire; Baliunas, Dolly; Mulder, Carol; Bolbocean, Corneliu; Selby, Peter | 2021 | Implementation Science | Exclusion reason: Wrong outcomes; |
| Mitton 2007 | Knowledge Transfer and Exchange: Review and Synthesis of the Literature | Mitton, Craig; Adair, Carol E.; McKenzie, Emily; Patten, Scott B.; Perry, Brenda Waye | 2007 | Milbank Quarterly | Exclusion reason: Wrong intervention; |
| Miyawaki 2018 | Champions of an Older Adult Exercise Program: Believers, Promoters, and Recruiters | Miyawaki, C. E.; Belza, B.; Kohn, M. J.; Petrescu-Prahova, M. | 2018 | Journal of Applied Gerontology | Exclusion reason: Wrong outcomes; |
| Monsivais 2018 | Data visualisation to support obesity policy: Case studies of data tools for planning and transport policy in the UK | Monsivais, Pablo; Francis, Oliver; Lovelace, Robin; Chang, Michael; Strachan, Emma; Burgoine, Thomas | 2018 | International Journal of Obesity | Exclusion reason: Wrong study design; |
| Moosa 2009 | Workshops to disseminate the Canadian Thoracic Society guidelines for chronic obstructive pulmonary disease to health care professionals in Ontario: impact on knowledge, perceived health care practices and participant satisfaction | Moosa, D.; Blouin, M.; Hill, K.; Goldstein, R. | 2009 | Canadian Respiratory Journal | Exclusion reason: Wrong intervention; |
| Morago 2010 | Dissemination and Implementation of Evidence-based Practice in the Social Services: A UK Survey | Morago, Pedro | 2010 | Journal of Evidence-Based Social Work | Exclusion reason: Wrong population; |
| MortÃ©nius 2012 | The utilization of knowledge of and interest in research and development among primary care staff by means of strategic communication - a staff cohort study | MortÃ©nius, Helena; Marklund, Bertil; Palm, Lars; Fridlund, Bengt; Baigi, Amir | 2012 | Journal of Evaluation in Clinical Practice | Exclusion reason: Wrong population; |
| Moskalewicz 2020 | EULAR PARE: Knowledge Transfer Programme | Moskalewicz, BoÅ¼ena; Grygielska, Jolanta | 2020 | Rheumatology / Reumatologia | Exclusion reason: Wrong study design; |
| Moss 2006 | Child health in complex emergencies | Moss, William J.; Ramakrishnan, Meenakshi; Storms, Dory; Siegle, Anne Henderson; Weiss, William M.; Lejnev, Ivan; Muhe, Lulu | 2006 | La salud infantil en las emergencias complejas. | Exclusion reason: Wrong intervention; |
| Motard 2016 | [Initiatives in urban health communities: Health promotion at work in deprived territories] | Motard, C.; Tessier, S. | 2016 | Sante Publique (Vandoeuvre-Les-Nancey) | Exclusion reason: Full text not available in English; |
| Murnaghan 2013 | Knowledge exchange systems for youth health and chronic disease prevention: a tri-provincial case study | Murnaghan, D.; Morrison, W.; Griffith, E. J.; Bell, B. L.; Duffley, L. A.; McGarry, K.; Manske, S. | 2013 |  | Exclusion reason: Wrong outcomes; |
| Murunga 2020 | Review of published evidence on knowledge translation capacity, practice and support among researchers and research institutions in low- and middle-income countries | Murunga, Violet Ibukayo; Oronje, Rose Ndakala; Bates, Imelda; Tagoe, Nadia; Pulford, Justin | 2020 | Health Research Policy & Systems | Exclusion reason: Wrong intervention; |
| NabyongaOrem 2012 | Do guidelines influence the implementation of health programs? -- Uganda's experience | Nabyonga Orem, Juliet; Bataringaya Wavamunno, Juliet; Bakeera, Solome K.; Criel, Bart | 2012 | Implementation Science | Exclusion reason: Wrong intervention; |
| Nakamura 2011 | Knowledge of and Attitudes Towards Evidence-Based Practices in Community Child Mental Health Practitioners | Nakamura, Brad; Higa-McMillan, Charmaine; Okamura, Kelsie; Shimabukuro, Scott | 2011 | Administration & Policy in Mental Health & Mental Health Services Research | Exclusion reason: Wrong intervention; |
| Namazzi 2015 | Designing for action: adapting and implementing a community-based newborn care package to affect national change in Uganda | Namazzi, Gertrude; Waiswa, Peter; Peterson, Stefan; Kerber, Kate | 2015 | Global Health Action | Exclusion reason: Wrong intervention; |
| Nanney 2007 | Awareness and adoption of a nationally disseminated dietary curriculum | Nanney, M. S.; Haire-Joshu, D.; Brownson, R. C.; Kostelc, J.; Stephen, M.; Elliott, M. | 2007 | American Journal of Health Behavior | Exclusion reason: Wrong population; |
| Napolitano 2002 | Targeting and tailoring physical activity information using print and information technologies | Napolitano, M. A.; Marcus, B. H. | 2002 | Exercise & Sport Sciences Reviews | Exclusion reason: Wrong study design; |
| Naylor 2001 | Enhancing capacity for cardiovascular disease prevention: an overview of the British Columbia Heart Health Dissemination Research Project | Naylor, P. J.; Wharf-Higgins, J.; O'Connor, B.; Odegard, L.; Blair, L. | 2001 | Promotion et Education | Exclusion reason: Wrong intervention; |
| Ndumbe-Eyoh 2016 | Social media, knowledge translation, and action on the social determinants of health and health equity: A survey of public health practices | Ndumbe-Eyoh, S.; Mazzucco, A. | 2016 | Journal of Public Health Policy | Exclusion reason: Wrong outcomes; |
| Newman 2020 | A mixed methods examination of knowledge brokers and their use of theoretical frameworks and evaluative practices | Newman, K.; DeForge, R.; Van Eerd, D.; Mok, Y. W.; Cornelissen, E. | 2020 | Health Research Policy & Systems | Exclusion reason: Wrong intervention; |
| Newson 2021 | The how and why of producing policy relevant research: perspectives of Australian childhood obesity prevention researchers and policy makers | Newson, Robyn; Rychetnik, Lucie; King, Lesley; Milat, Andrew J.; Bauman, Adrian E. | 2021 | Health Research Policy & Systems | Exclusion reason: Wrong outcomes; |
| Newton 2007 | Health researchers in Alberta: an exploratory comparison of defining characteristics and knowledge translation activities | Newton, Mandi S.; Estabrooks, Carole A.; Norton, Peter; Birdsell, Judy M.; Adewale, Adeniyi J.; Thornley, Richard | 2007 | Implementation Science | Exclusion reason: Wrong population; |
| Ngamo 2016 | Do knowledge translation (KT) plans help to structure KT practices? | Ngamo, Salomon Tchameni; Souffez, Karine; Lord, Catherine; Dagenais, Christian; Tchameni Ngamo, Salomon | 2016 | Health Research Policy & Systems | Exclusion reason: Wrong intervention; |
| Nhim 2019 | Using a RE-AIM framework to identify promising practices in National Diabetes Prevention Program implementation | Nhim, K.; Gruss, S. M.; Porterfield, D. S.; Jacobs, S.; Elkins, W.; Luman, E. T.; Van Aacken, S.; Schumacher, P.; Albright, A. | 2019 | Implementation Science | Exclusion reason: Wrong intervention; |
| Niehaus 2018 | The PM&R Journal Implements a Social Media Strategy to Disseminate Research and Track Alternative Metrics in Physical Medicine and Rehabilitation | Niehaus, W. N.; Silver, J. K.; Katz, M. S. | 2018 | Pm & R | Exclusion reason: Wrong population; |
| Nordvik 2016 | The use of virtual meetings and a project platform bring researchers and clinicians together in Knowledge Translation (KT) | Nordvik, Jan Egil; Berg, Marie; Mbalilaki, Julia Aneth; BjÃ¸rkli, Cato A.; LÃ¸mo, Linn Lien; Midthaug, Mari; Skjuve, Marita; Havdahl, Richard Tidemann; Moore, Jennifer | 2016 | International Journal of Integrated Care (IJIC) | Exclusion reason: Wrong population; |
| Norton 2016 | Exploratory study of the role of knowledge brokers in translating knowledge to action following global maternal and newborn health technical meetings | Norton, T. C.; Howell, C.; Reynolds, C. | 2016 | Public Health | Exclusion reason: Wrong intervention; |
| Norton 2019 | Applying the Theoretical Domains Framework to understand knowledge broker decisions in selecting evidence for knowledge translation in low- and middle-income countries | Norton, Theresa C.; Rodriguez, Daniela C.; Willems, Sara | 2019 | Health Research Policy & Systems | Exclusion reason: Wrong intervention; |
| Nothwehr 2014 | Statewide dissemination of a rural, non-chain restaurant intervention: adoption, implementation and maintenance | Nothwehr, F.; Haines, H.; Chrisman, M.; Schultz, U. | 2014 | Health Education Research | Exclusion reason: Wrong intervention; |
| Nour 2016 | Evaluation of the effects of health impact assessment practice at the local level in Monteregie | Nour, K.; Dutilly-Simard, S.; Brousselle, A.; Smits, P.; Buregeya, J. M.; Loslier, J.; Denis, J. L. | 2016 | Health Research Policy & Systems | Exclusion reason: Wrong intervention; |
| Novak 2018 | Expertise and development of Croatian mental health policy: The perception of mental health professionals | Novak, Miranda; Petek, Ana | 2018 | Socijalna Psihijatrija | Exclusion reason: Wrong intervention; |
| NystrÃ¶m 2015 | Locally based research and development units as knowledge brokers and change facilitators in health and social care of older people in Sweden | NystrÃ¶m, Monica Elisabeth; Hansson, Johan; Garvare, Rickard; Andersson-BÃ¤ck, Monica | 2015 | Evidence & Policy: A Journal of Research, Debate & Practice | Exclusion reason: Wrong intervention; |
| Oâ€™Brien 2019 | Lessons learned from a cancer knowledge translation grants program: results of an evaluation | Oâ€™Brien, M. A.; Makuwaza, T.; Graham, I. D.; Barbera, L.; Earle, C. C.; Brouwers, M. C.; Grunfeld, E. | 2019 | Current Oncology | Exclusion reason: Wrong intervention; |
| O'Brien 2018 | Variable participation of knowledge users in cancer health services research: results of a multiple case study | O'Brien, Mary Ann; Carson, Andrea; Barbera, Lisa; Brouwers, Melissa C.; Earle, Craig C.; Graham, Ian D.; Mittmann, Nicole; Grunfeld, Eva | 2018 | BMC Medical Research Methodology | Exclusion reason: Wrong intervention; |
| OlivaresCortes 2015 | [Design and Validation of an Image for Dissemination and Implementation of Chilean Dietary Guidelines] | Olivares Cortes, S.; Zacarias Hasbun, I.; Gonzalez Gonzalez, C. G.; Fonseca Moran, L.; Mediano Stoltze, F.; Pinheiro Fernandes, A. C.; Rodriguez Osiac, L. | 2015 | Nutricion Hospitalaria | Exclusion reason: Full text not available in English; |
| Olstad 2015 | A multiple case history and systematic review of adoption, diffusion, implementation and impact of provincial daily physical activity policies in Canadian schools | Olstad, Dana Lee; Campbell, Elizabeth J.; Raine, Kim D.; Nykiforuk, Candace I. J. | 2015 | BMC Public Health | Exclusion reason: Wrong intervention; |
| Ongolo-Zogo 2018 | Assessing the influence of knowledge translation platforms on health system policy processes to achieve the health millennium development goals in Cameroon and Uganda: a comparative case study | Ongolo-Zogo, P.; Lavis, J. N.; Tomson, G.; Sewankambo, N. K. | 2018 | Health Policy & Planning | Exclusion reason: Wrong intervention; |
| Orgill 2019 | A qualitative study of the dissemination and diffusion of innovations: bottom up experiences of senior managers in three health districts in South Africa | Orgill, Marsha; Gilson, Lucy; Chitha, Wezile; Michel, Janet; Erasmus, Ermin; Marchal, Bruno; Harris, Bronwyn | 2019 | International Journal for Equity in Health | Exclusion reason: Wrong intervention; |
| Osterling 2008 | The Dissemination and Utilization of Research for Promoting Evidence-Based Practice | Osterling, Kathy Lemon; Austin, Michael J. | 2008 | Journal of Evidence-Based Social Work | Exclusion reason: Wrong study design; |
| OstersenMukai 2012 | Use of hyperlinks in electronic test result communication: a survey study in general practice | Ostersen Mukai, Thomas; Bro, Flemming; Fenger-Grâ€ºn, Morten; Olesen, Frede; Vedsted, Vedsted | 2012 | BMC Medical Informatics & Decision Making | Exclusion reason: Wrong intervention; |
| Owen 2006 | Evidence-based approaches to dissemination and diffusion of physical activity interventions | Owen, N.; Glanz, K.; Sallis, J. F.; Kelder, S. H. | 2006 | American Journal of Preventive Medicine | Exclusion reason: Wrong study design; |
| Page-Reeves 2015 | Understanding 'Agency' in the Translation of a Health Promotion Program | Page-Reeves, Janet; Davis, Sally; Romero, Camilla; Chrisp, Eric | 2015 | Prevention Science | Exclusion reason: Wrong outcomes; |
| Panda 2015 | System level approaches for mainstreaming tobacco control into existing health programs in India: Perspectives from the field | Panda, Rajmohan; Srivastava, Swati; Persai, Divya; Mendenhall, Emily; Arora, Monika; Mathur, Manu Raj | 2015 | Journal of Family Medicine & Primary Care | Exclusion reason: Wrong intervention; |
| Papadakis 2016 | Increasing Rates of Tobacco Treatment Delivery in Primary Care Practice: Evaluation of the Ottawa Model for Smoking Cessation | Papadakis, Sophia; Cole, Adam G.; Reid, Robert D.; Coja, Mustafa; Aitken, Debbie; Mullen, Kerri-Anne; Gharib, Marie; Pipe, Andrew L. | 2016 | Annals of Family Medicine | Exclusion reason: Wrong intervention; |
| Park 2016 | Information needs of Botswana health care workers and perceptions of wikipedia | Park, Elizabeth; Masupe, Tiny; Joseph, Joseph; Ho-Foster, Ari; Chavez, Afton; Jammalamadugu, Swetha; Marek, Andrew; Arumala, Ruth; Ketshogileng, Dineo; Littman-Quinn, Ryan; Kovarik, Carrie | 2016 | International Journal of Medical Informatics | Exclusion reason: Wrong population; |
| Parrott 2004 | Emphasizing "Communication" in Health Communication | Parrott, Roxanne | 2004 | Journal of Communication | Exclusion reason: Wrong study design; |
| Paul 2007 | From Evidence-Based Practice Making to Practice-Based Evidence Making: Creating Communities of (Research) and Practice | Paul, W. McDonald | 2007 | Health Promotion Practice | Exclusion reason: Wrong study design; |
| Perry 2017 | The adaptation and translation of the PEACHâ„¢ RCT intervention: the process and outcomes of the PEACHâ„¢ in the community trial | Perry, R. A.; Golley, R. K.; Hartley, J.; Magarey, A. M. | 2017 | Public Health (Elsevier) | Exclusion reason: Wrong outcomes; |
| Petkovic 2016 | The effectiveness of evidence summaries on health policymakers and health system managers use of evidence from systematic reviews: a systematic review | Petkovic, Jennifer; Welch, Vivian; Jacob, Maria Helena; Yoganathan, Manosila; Ayala, Ana Patricia; Cunningham, Heather; Tugwell, Peter | 2016 | Implementation Science | Exclusion reason: Wrong population; |
| Pope 2018 | Starting Off on the Best Foot: A Review of Message Framing and Message Tailoring, and Recommendations for the Comprehensive Messaging Strategy for Sustained Behavior Change | Pope, J. Paige; Pelletier, Luc; Guertin, Camille | 2018 | Health Communication | Exclusion reason: Wrong population; |
| PoplaÅ¡en 2020 | INFOGRAFIKA KAO OBLIK KOMUNIKACIJE NA PODRUÄŒJU ZAÅ TITE ZDRAVLJA I SIGURNOSTI NA RADU | PoplaÅ¡en, L. Machala; DrauÅ¡nik, Å½; VoÄanec, D.; BrboroviÄ‡, H. | 2020 | INFOGRAPHICS AS A COMMUNICATION TOOL IN HEALTH AND SAFETY AT WORK. | Exclusion reason: Full text not available in English; |
| Pringle 2004 | An Examination of Infrastructures for Health Information Dissemination in the United States | Pringle, Kristine E.; Wells, Rebecca; Merrill, Sonya | 2004 | Science Communication | Exclusion reason: Wrong outcomes; |
| Purtle 2020 | Toward the data-driven dissemination of findings from psychological science | Purtle, J.; Marzalik, J. S.; Halfond, R. W.; Bufka, L. F.; Teachman, B. A.; Aarons, G. A. | 2020 | American Psychologist | Exclusion reason: Wrong intervention; |
| Purtle 2020 | Dissemination Strategies to Accelerate the Policy Impact of Children's Mental Health Services Research | Purtle, J.; Nelson, K. L.; Bruns, E. J.; Hoagwood, K. E. | 2020 | Psychiatr Serv | Exclusion reason: Wrong study design; |
| Rabin 2006 | Methodologic challenges in disseminating evidence-based interventions to promote physical activity | Rabin, B. A.; Brownson, R. C.; Kerner, J. F.; Glasgow, R. E. | 2006 | American Journal of Preventive Medicine | Exclusion reason: Wrong study design; |
| Raczynski 2009 | Arkansas Act 1220 of 2003 to reduce childhood obesity: its implementation and impact on child and adolescent body mass index | Raczynski, J. M.; Thompson, J. W.; Phillips, M. M.; Ryan, K. W.; Cleveland, H. W. | 2009 | Journal of Public Health Policy | Exclusion reason: Wrong intervention; |
| Rajic 2013 | Improving the utilization of research knowledge in agri-food public health: a mixed-method review of knowledge translation and transfer | Rajic, A.; Young, I.; McEwen, S. A. | 2013 | Foodborne Pathogens & Disease | Exclusion reason: Wrong outcomes; |
| Rashidian 2008 | Falling on stony ground? A qualitative study of implementation of clinical guidelinesâ€™ prescribing recommendations in primary care | Rashidian, Arash; Eccles, Martin P.; Russell, Ian | 2008 | Health Policy | Exclusion reason: Wrong intervention; |
| Raven 2016 | Using guidelines to improve neonatal health in China and Vietnam: a qualitative study | Raven, Joanna; Xiaoyun, Liu; Dan, Hu; Weiming, Zhu; Dinh Thi Phuong, Hoa; Le Minh, Thi; Doan Thi Thuy, Duong; Alonso-Garbayo, Alvaro; Martineau, Tim | 2016 | BMC Health Services Research | Exclusion reason: Wrong intervention; |
| Ray 2014 | Nutrition education and leadership for improved clinical outcomes: training and supporting junior doctors to run 'Nutrition Awareness Weeks' in three NHS hospitals across England | Ray, S.; Laur, C.; Douglas, P.; Rajput-Ray, M.; van der Es, M.; Redmond, J.; Eden, T.; Sayegh, M.; Minns, L.; Griffin, K.; McMillan, C.; Adiamah, A.; Gillam, S.; Gandy, J. | 2014 | BMC Medical Education | Exclusion reason: Wrong intervention; |
| Reich 2010 | The effectiveness of baby books for providing pediatric anticipatory guidance to new mothers | Reich, S. M.; Bickman, L.; Saville, B. R.; Alvarez, J. | 2010 | Pediatrics | Exclusion reason: Wrong population; |
| Reid 2016 | Participants' perception of a unique community of practice for substance abuse education in the Caribbean | Reid, S. D.; Downes, E.; Khenti, A. | 2016 | Substance Abuse | Exclusion reason: Wrong intervention; |
| Remington 2009 | Dissemination research: the University of Wisconsin Population Health Institute | Remington, P. L.; Moberg, D. P.; Booske, B. C.; Ceraso, M.; Friedsam, D.; Kindig, D. A. | 2009 | WMJ | Exclusion reason: Wrong study design; |
| Richard 2018 | Organizational Knowledge Creation in the Context of a Professional Development Program: Mixed-Methods Longitudinal Results From the ALPS Study | Richard, Lucie; Chiocchio, FranÃ§ois; Morales Hudon, Anahi; Fortin-Pellerin, Laurence; Litvak, Ã‰ric; Beaudet, Nicole | 2018 | Pedagogy in Health Promotion | Exclusion reason: Wrong intervention; |
| Ridde 2013 | [An exploratory synthesis of knowledge brokering in public health] | Ridde, V.; Dagenais, C.; Boileau, M. | 2013 | Sante Publique (Vandoeuvre-Les-Nancey) | Exclusion reason: Full text not available in English; |
| Risendal 2020 | Impact of Cancer Survivorship Care Training on Rural Primary Care Practice Teams: a Mixed Methods Approach | Risendal, B.; Westfall, J. M.; Zittleman, L.; Hodgson, C.; Garrington, T.; High Plains Research Network Community Advisory, Council; Sutter, C.; Jarrell, L.; LeBlanc, W.; Overholser, L. | 2020 | Journal of Cancer Education | Exclusion reason: Wrong intervention; |
| Robinson 2007 | From heart health promotion to chronic disease prevention: contributions of the Canadian Heart Health Initiative | Robinson, K.; Farmer, T.; Elliott, S. J.; Eyles, J. | 2007 | Preventing Chronic Disease | Exclusion reason: Wrong intervention; |
| Rod 2016 | A case of standardization? Implementing health promotion guidelines in Denmark | Rod, M. H.; Hoybye, M. T. | 2016 | Health Promotion International | Exclusion reason: Wrong intervention; |
| RodrÃ­guez-SalvanÃ©s 2011 | Efficacy of a strategy for implementing a guideline for the control of cardiovascular risk in a primary healthcare setting: the SIRVA2 study a controlled, blinded community intervention trial randomised by clusters | RodrÃ­guez-SalvanÃ©s, Francisco; Novella, Blanca; Luque, MarÃ­a JesÃºs FernÃ¡ndez; SÃ¡nchez-GÃ³mez, Luis MarÃ­a; Ruiz-DÃ­az, Lourdes; SÃ¡nchez-Alcalde, Rosa; Sierra-GarcÃ­a, BelÃ©n; Mayayo, Soledad; Ruiz-LÃ³pez, Marta; Loeches, Pilar; LÃ³pez-GÃ³nzÃ¡lez, Javier; GonzÃ¡lez-Gamarra, Amelia | 2011 | BMC Family Practice | Exclusion reason: Wrong outcomes; |
| Rogers 2012 | Building the links between surveillance, research, and policy and practice - dental public health experiences in Australia | Rogers, John | 2012 | Community Dentistry & Oral Epidemiology | Exclusion reason: Wrong study design; |
| Rohrbach 2010 | Dissemination of Project Towards No Drug Abuse (TND): findings from a survey of program adopters | Rohrbach, L. A.; Gunning, M.; Grana, R.; Gunning, G.; Sussman, S. | 2010 | Substance Use & Misuse | Exclusion reason: Wrong intervention; |
| Rosella 2018 | Evaluating the process and outcomes of a knowledge translation approach to supporting use of the Diabetes Population Risk Tool (DPoRT) in public health practice | Rosella, Laura C.; Bornbaum, Catherine; Kornas, Kathy; Lebenbaum, Michael; Peirson, Leslea; Fransoo, Randy; Loeppky, Carla; Gardner, Charles; Mowat, David | 2018 | Canadian Journal of Program Evaluation | Exclusion reason: Wrong outcomes; |
| Rositch 2020 | The role of dissemination and implementation science in global breast cancer control programs: Frameworks, methods, and examples | Rositch, Anne F.; Ungerâ€SaldaÃ±a, Karla; DeBoer, Rebecca J.; Ng'ang'a, Anne; Weiner, Bryan J.; Unger-SaldaÃ±a, Karla | 2020 | Cancer (0008543X) | Exclusion reason: Wrong intervention; |
| Rowe 2017 | A complex postnatal mental health intervention: Australian translational formative evaluation | Rowe, Heather J.; Wynter, Karen H.; Burns, Joanna K.; Fisher, Jane R. | 2017 | Health Promotion International | Exclusion reason: Wrong intervention; |
| Rushmer 2014 | Using interactive workshops to prompt knowledge exchange: a realist evaluation of a knowledge to action initiative | Rushmer, R. K.; Hunter, D. J.; Steven, A. | 2014 | Public Health (Elsevier) | Exclusion reason: Wrong intervention; |
| SÃ¡nchez 2016 | Type-2 diabetes primary prevention program implemented in routine primary care: a process evaluation study | SÃ¡nchez, Alvaro; Silvestre, Carmen; Campo, Natalia; Grandes, Gonzalo; Pre, D. E. research group | 2016 | Trials [Electronic Resource] | Exclusion reason: Wrong intervention; |
| Saha 2005 | Effectiveness of different methods of health education: a comparative assessment in a scientific conference | Saha, A.; Poddar, E.; Mankad, M. | 2005 | BMC Public Health | Exclusion reason: Wrong population; |
| Salter 2014 | Using realist evaluation to open the black box of knowledge translation: a state-of-the-art review | Salter, Katherine L.; Kothari, Anita | 2014 | Implementation Science | Exclusion reason: Wrong population; |
| Sarkies 2019 | Video strategies improved health professional knowledge across different contexts: a helix counterbalanced randomized controlled study | Sarkies, M. N.; Maloney, S.; Symmons, M.; Haines, T. P. | 2019 | Journal of Clinical Epidemiology | Exclusion reason: Wrong intervention; |
| Schumacher 2017 | How dietary evidence for the prevention and treatment of CVD is translated into practice in those with or at high risk of CVD: a systematic review | Schumacher, Tracy L.; Burrows, Tracy L.; Neubeck, Lis; Redfern, Julie; Callister, Robin; Collins, Clare E. | 2017 | Public Health Nutrition | Exclusion reason: Wrong outcomes; |
| Scull 2021 | Promoting Sexual Health in High School: A Feasibility Study of A Web-based Media Literacy Education Program | Scull, Tracy; Malik, Christina; Morrison, Abigail; Keefe, Elyse | 2021 | Journal of Health Communication | Exclusion reason: Wrong population; |
| SerranoBarrera 2017 | Sitio web para diseminar contenidos y recursos sobre las aplicaciones clÃ­nicas de las tecnologÃ­as Ã³micas | Serrano Barrera, Orlando Rafael; de la Caridad HernÃ¡ndez Betancourt, Jenny | 2017 | Website to disseminate contents and resources on the clinical applications of genomic technologies. | Exclusion reason: Full text not available in English; |
| Shelton 2020 | What Is Dissemination and Implementation Science?: An Introduction and Opportunities to Advance Behavioral Medicine and Public Health Globally | Shelton, R. C.; Lee, M.; Brotzman, L. E.; Wolfenden, L.; Nathan, N.; Wainberg, M. L. | 2020 | International Journal of Behavioral Medicine | Exclusion reason: Wrong outcomes; |
| Shi 2018 | Identifying Opinion Leaders to Promote Organ Donation on Social Media: Network Study | Shi, J.; Salmon, C. T. | 2018 | Journal of Medical Internet Research | Exclusion reason: Wrong intervention; |
| Shroff 2015 | Incorporating research evidence into decision-making processes: researcher and decision-maker perceptions from five low- and middle-income countries | Shroff, Zubin; Aulakh, Bhupinder; Gilson, Lucy; Agyepong, Irene A.; El-Jardali, Fadi; Ghaffar, Abdul | 2015 | Health Research Policy & Systems | Exclusion reason: Wrong outcomes; |
| Sibley 2017 | A descriptive qualitative examination of knowledge translation practice among health researchers in Manitoba, Canada | Sibley, Kathryn; Roche, Patricia; Bell, Courtney; Temple, Beverley; Wittmeier, Kristy; Sibley, Kathryn M.; Roche, Patricia L.; Bell, Courtney P.; Wittmeier, Kristy D. M. | 2017 | BMC Health Services Research | Exclusion reason: Wrong intervention; |
| Slater 2005 | Incorporation of a successful community-based mammography intervention: dissemination beyond a community trial | Slater, J. S.; Finnegan, J. R., Jr.; Madigan, S. D. | 2005 | Health Psychology | Exclusion reason: Wrong outcomes; |
| SlevinPerocchia 2005 | Raising Awareness of On-line Cancer Information: Helping Providers Empower Patients | Slevin Perocchia, Rosemarie; Rapkin, Bruce; Keany Hodorowski, Julie; Lassalle Davis, Nydia; Redrick Mcfarlane, Anita; Carpenter, Rose | 2005 | Journal of Health Communication | Exclusion reason: Wrong intervention; |
| Smith 2015 | A gender-informed model to train community health workers in maternal mental health | Smith, Megan V.; Kruse-Austin, Anna | 2015 | Evaluation & Program Planning | Exclusion reason: Wrong intervention; |
| SolÃ  2014 | Attitudes and Perceptions about Clinical Guidelines: A Qualitative Study with Spanish Physicians | SolÃ , Ivan; Carrasco, JosÃ© Miguel; DÃ­az del Campo, Petra; Gracia, Javier; Orrego, Carola; MartÃ­nez, Flora; Kotzeva, Anna; GuillamÃ³n, Imma; CalderÃ³n, Enrique; de Gaminde, Idoia; Louro, Arturo; Rotaeche, Rafael; Salcedo, Flavia; VelÃ¡zquez, Paola; Alonso-Coello, Pablo | 2014 | PLoS ONE [Electronic Resource] | Exclusion reason: Wrong population; |
| Spagnolo 2020 | Reflecting on knowledge translation strategies from global health research projects in Tunisia and the Republic of Cote d'Ivoire | Spagnolo, J.; Gautier, L.; Champagne, F.; Leduc, N.; Melki, W.; N'Guessan, K.; Charfi, F. | 2020 | International Journal of Public Health | Exclusion reason: Wrong outcomes; |
| Stephens 2004 | Expanding the Reach of Health Campaigns: Community Organizations as Meta-Channels for the Dissemination of Health Information | Stephens, Kerik; Rimal, Rajivn; Flora, Junea | 2004 | Journal of Health Communication | Exclusion reason: Wrong population; |
| Sugawara 2016 | Medical Institutions and Twitter: A Novel Tool for Public Communication in Japan | Sugawara, Y.; Narimatsu, H.; Tsuya, A.; Tanaka, A.; Fukao, A. | 2016 | JMIR Public Health and Surveillance | Exclusion reason: Wrong population; |
| Sullivan 2009 | The Impact of Training and Other Variables on the Preparation of the Public Welfare Workforce | Sullivan, DanaJ; Antle, BeckyF; Barbee, AnitaP; Egbert, Rich | 2009 | Administration in Social Work | Exclusion reason: Wrong intervention; |
| Sultana 2017 | The eToolkit for Field Workers: A digital library of SBCC materials to support integrated counseling | Sultana, Zeenat; Shahjahan, Mohammad; Biplob, Mohammad Khairul Abedin | 2017 | International Journal of Integrated Care (IJIC) | Exclusion reason: Wrong study design; |
| Summerlin-Long 2009 | Promoting tobacco-free school policies through a statewide media campaign | Summerlin-Long, S. K.; Goldstein, A. O.; Davis, J.; Shah, V. | 2009 | Journal of School Health | Exclusion reason: Wrong intervention; |
| Syed 2017 | A review on community-based knowledge transfer and exchange (KTE) initiatives for promoting well-being in older adults | Syed, M.; Moorhouse, Aynsley; McDonald, Lynn; Hitzig, Sander L. | 2017 | Journal of Evidence-Informed Social Work | Exclusion reason: Wrong intervention; |
| Syed 2019 | Knowledge translation facilitating co-creation of evidence in public health | Syed, M. A. | 2019 | BMJ Evidence-based Medicine | Exclusion reason: Wrong study design; |
| Tate 2019 | Learning to lead: a review and synthesis of literature examining health care managers' use of knowledge | Tate, Kaitlyn; Hewko, Sarah; McLane, Patrick; Baxter, Pamela; Perry, Karyn; Armijo-Olivo, Susan; Estabrooks, Carole; Gordon, Deb; Cummings, Greta | 2019 | Journal of Health Services Research & Policy | Exclusion reason: Wrong intervention; |
| Tetroe 2008 | Health research funding agencies' support and promotion of knowledge translation: An international study | Tetroe, Jacqueline M.; Graham, Ian D.; Foy, Robbie; Robinson, Nicole; Eccles, Martin P.; Wensing, Michel; Durieux, Pierre; Legare, France; Nielson, Camilla Palmhoj; Adily, Armita; Ward, Jeanette E.; Porter, Cassandra; Shea, Beverley; Grimshaw, Jeremy M. | 2008 | Milbank Quarterly | Exclusion reason: Wrong intervention; |
| Tilson 2015 | Dissemination and Adoption of Guidelines: The Experience of Community Care of North Carolina | Tilson, E. C. | 2015 | North Carolina Medical Journal | Exclusion reason: Wrong study design; |
| Tinkle 2013 | Dissemination and Implementation Research Funded by the US National Institutes of Health, 2005-2012 | Tinkle, M.; Kimball, R.; Haozous, E. A.; Shuster, G.; Meize-Grochowski, R. | 2013 | Nursing Research and Practice | Exclusion reason: Wrong outcomes; |
| Tiura 2019 | Impact of Behavioral Risk Factor Surveillance System data on public health outcomes within States in the United States | Tiura, Melanee L. | 2019 | Dissertation Abstracts International: Section B: The Sciences and Engineering | Exclusion reason: Wrong intervention; |
| Toews 2016 | Extent, Awareness and Perception of Dissemination Bias in Qualitative Research: An Explorative Survey | Toews, Ingrid; Glenton, Claire; Lewin, Simon; Berg, Rigmor C.; Noyes, Jane; Booth, Andrew; Marusic, Ana; Malicki, Mario; Munthe-Kaas, Heather M.; Meerpohl, Joerg J. | 2016 | PLoS ONE [Electronic Resource] | Exclusion reason: Wrong intervention; |
| Tomasone 2015 | Changing minds, changing lives from the top down: An investigation of the dissemination and adoption of a Canada-wide educational intervention to enhance health care professionals' intentions to prescribe physical activity | Tomasone, Jennifer R.; Martin Ginis, Kathleen A.; Estabrooks, Paul A.; Domenicucci, Laura | 2015 | International Journal of Behavioral Medicine | Exclusion reason: Wrong intervention; |
| Tomasone 2020 | Knowledge translation of the Canadian 24-Hour Movement Guidelines for Adults aged 18â€“64 years and Adults aged 65 years or older: a collaborative movement guideline knowledge translation process | Tomasone, Jennifer R.; Flood, Stephanie M.; Latimer-Cheung, Amy E.; Faulkner, Guy; Duggan, Mary; Jones, Rebecca; Lane, Kirstin N.; Bevington, Frances; Carrier, Julie; Dolf, Matt; Doucette, Kevin; Faught, Emma; Gierc, Madelaine; Giouridis, Nicole; Gruber, Reut; Johnston, Nora; Kauffeldt, Kaitlyn D.; Kennedy, William; Lorbergs, Amanda; Maclaren, Kaleigh | 2020 | Applied Physiology, Nutrition & Metabolism | Exclusion reason: Wrong intervention; |
| Traynor 2014 | Knowledge brokering in public health: a tale of two studies | Traynor, R.; DeCorby, K.; Dobbins, M. | 2014 | Public Health | Exclusion reason: Wrong outcomes; |
| vanderGraaf 2019 | Performing collaborative research: a dramaturgical reflection on an institutional knowledge brokering service in the North East of England | van der Graaf, Peter; Shucksmith, Janet; Rushmer, Rosemary; Rhodes, Avril; Welford, Mark | 2019 | Health Research Policy & Systems | Exclusion reason: Wrong study design; |
| VanEerd 2016 | Knowledge brokering for healthy aging: a scoping review of potential approaches | Van Eerd, D.; Newman, K.; DeForge, R.; Urquhart, R.; Cornelissen, E.; Dainty, K. N. | 2016 | Implementation Science | Exclusion reason: Wrong outcomes; |
| vanNassau 2016 | Barriers and facilitators to the nationwide dissemination of the Dutch school-based obesity prevention programme DOiT | van Nassau, F.; Singh, A. S.; Broekhuizen, D.; van Mechelen, W.; Brug, J.; Chinapaw, M. J. | 2016 | European Journal of Public Health | Exclusion reason: Wrong intervention; |
| Vaucher 2016 | Meeting physicians' needs: a bottom-up approach for improving the implementation of medical knowledge into practice | Vaucher, Carla; Bovet, Emilie; Bengough, Theresa; Pidoux, Vincent; Grossen, MichÃ¨le; Panese, Francesco; Burnand, Bernard | 2016 | Health Research Policy & Systems | Exclusion reason: Wrong population; |
| Velasco 2015 | A large-scale initiative to disseminate an evidence-based drug abuse prevention program in Italy: Lessons learned for practitioners and researchers | Velasco, V.; Griffin, K. W.; Antichi, M.; Celata, C. | 2015 | Evaluation & Program Planning | Exclusion reason: Wrong intervention; |
| Vine 2019 | An early implementation assessment of Ontario's Healthy Kids Community Challenge: results from a survey of key stakeholders | Vine, M. M.; Jarvis, J. W.; Chong, E.; Laxer, R. E.; Ladak, A.; Manson, H. | 2019 | BMC Public Health | Exclusion reason: Wrong intervention; |
| Visram 2014 | Exploring conceptualizations of knowledge translation, transfer and exchange across public health in one UK region: a qualitative mapping study | Visram, S.; Goodall, D.; Steven, A. | 2014 | Public Health | Exclusion reason: Wrong intervention; |
| Voss 2013 | Tracking uptake of innovations from the European Union Public Health Programme | Voss, M.; Alexanderson, K.; McCarthy, M. | 2013 | European Journal of Public Health | Exclusion reason: Wrong intervention; |
| Waters 2011 | An exploratory cluster randomised controlled trial of knowledge translation strategies to support evidence-informed decision-making in local governments (The KT4LG study) | Waters, E.; Armstrong, R.; Swinburn, B.; Moore, L.; Dobbins, M.; Anderson, L.; Petticrew, M.; Clark, R.; Conning, R.; Moodie, M.; Carter, R. | 2011 | BMC Public Health | Exclusion reason: Wrong study design; |
| Werner 2008 | Healthcare provider back pain beliefs unaffected by a media campaign | Werner, Erik L.; Gross, Douglas P.; Atle Lie, Stein; IhlebÃ¦k, Camilla | 2008 | Scandinavian Journal of Primary Health Care | Exclusion reason: Wrong intervention; |
| Wexler 2019 | Community mobilization for rural suicide prevention: Process, learning and behavioral outcomes from Promoting Community Conversations About Research to End Suicide (PC CARES) in Northwest Alaska | Wexler, Lisa; Rataj, Suzanne; Ivanich, Jerreed; Plavin, Jya; Mullany, Anna; Moto, Roberta; Kirk, Tanya; Goldwater, Eva; Johnson, Rhonda; Dombrowski, Kirk | 2019 | Social Science & Medicine | Exclusion reason: Wrong intervention; |
| White 2014 | Summary of: Continuing professional development and application of knowledge from research findings: a qualitative study of general dental practitioners | White, Deborah | 2014 | British Dental Journal | Exclusion reason: Wrong study design; |
| Wilcox 2010 | Adoption and implementation of physical activity and dietary counseling by community health center providers and nurses | Wilcox, S.; Parra-Medina, D.; Felton, G. M.; Poston, M. B.; McClain, A. | 2010 | Journal of Physical Activity & Health | Exclusion reason: Wrong intervention; |
| Wilkinson 2009 | The role of the information specialist in supporting knowledge transfer: a public health information case study | Wilkinson, A.; Papaioannou, D.; Keen, C.; Booth, A. | 2009 |  | Exclusion reason: Wrong outcomes; |
| Williams 2014 | Characteristics of Community Health Organizations and Decision-Makers Considering the Adoption of Motivational Interviewing | Williams, Jessica; Dusablon, Tracy; Williams, Weston; Blais, Marissa; Hennessy, Kevin | 2014 | Journal of Behavioral Health Services & Research | Exclusion reason: Wrong intervention; |
| Williams 2021 | Using Implementation Science to Disseminate a Lung Cancer Screening Education Intervention Through Community Health Workers | Williams, L. B.; Shelton, B. J.; Gomez, M. L.; Al-Mrayat, Y. D.; Studts, J. L. | 2021 | Journal of Community Health | Exclusion reason: Wrong population; |
| Wolbring 2021 | How to disseminate national recommendations for physical activity: a qualitative analysis of critical change agents in Germany | Wolbring, L.; Reimers, A. K.; Niessner, C.; Demetriou, Y.; Schmidt, S. C. E.; Woll, A.; Wasche, H. | 2021 | Health Research Policy & Systems | Exclusion reason: Wrong intervention; |
| Wright 2018 | A novel approach to sharing all available information from funded health research: the NIHR Journals Library | Wright, David; Williams, Elaine; Bryce, Colin; le May, AndrÃ©e; Stein, Ken; Milne, Ruairidh; Walley, Tom | 2018 | Health Research Policy & Systems | Exclusion reason: Wrong study design; |
| Yancey 2006 | Dissemination of physical activity promotion interventions in underserved populations | Yancey, A. K.; Ory, M. G.; Davis, S. M. | 2006 | American Journal of Preventive Medicine | Exclusion reason: Wrong study design; |
| Yarber 2015 | Evaluating a train-the-trainer approach for improving capacity for evidence-based decision making in public health | Yarber, L.; Brownson, C. A.; Jacob, R. R.; Baker, E. A.; Jones, E.; Baumann, C.; Deshpande, A. D.; Gillespie, K. N.; Scharff, D. P.; Brownson, R. C. | 2015 | BMC Health Services Research | Exclusion reason: Wrong intervention; |
| Yost 2016 | Promoting Awareness of Key Resources for Evidence-Informed Decision-making in Public Health: An Evaluation of a Webinar Series about Knowledge Translation Methods and Tools | Yost, J.; Mackintosh, J.; Read, K.; Dobbins, M. | 2016 | Frontiers in Public Health | Exclusion reason: Wrong intervention; |
| Young 2003 | Randomised trial of intensive academic detailing to promote opportunistic recruitment of women to cervical screening by general practitioners | Young, J. M.; Ward, J. E. | 2003 | Australian & New Zealand Journal of Public Health | Exclusion reason: Wrong intervention; |
| Young 2021 | Harnessing the power of storytelling in online public health campaigns: An evaluation of the bring your brave campaign | Young, Monique P. | 2021 | Dissertation Abstracts International: Section B: The Sciences and Engineering | Exclusion reason: Wrong population; |
| Yu 2018 | Impact of the Canadian Diabetes Association guideline dissemination strategy on clinician knowledge and behaviour change outcomes | Yu, Catherine H.; Lillie, Erin; Mascarenhas-Johnson, Alekhya; Gall Casey, Carolyn; Straus, Sharon E. | 2018 | Diabetes Research & Clinical Practice | Exclusion reason: Wrong intervention; |
| Yu 2019 | Process Evaluation of the Diabetes Canada Guidelines Dissemination Strategy Using the Reach Effectiveness Adoption Implementation Maintenance (RE-AIM) Framework | Yu, C. H.; Gall Casey, C.; Ke, C.; Lebovic, G.; Straus, S. E. | 2019 | Canadian Journal of Diabetes | Exclusion reason: Wrong intervention; |
| Zardo 2014 | Predicting research use in a public health policy environment: results of a logistic regression analysis | Zardo, P.; Collie, A. | 2014 | Implementation Science | Exclusion reason: Wrong population; |
| Zdunek 2021 | Tailored communication methods as key to implementation of evidence-based solutions in primary child health care | Zdunek, Kinga; SchrÃ¶der-BÃ¤ck, Peter; Alexander, Denise; Vlasblom, Eline; Kocken, Paul; Rigby, Michael; Blair, Mitch | 2021 | European Journal of Public Health | Exclusion reason: Wrong intervention; |
| Zhao 2020 | Knowledge translation strategies designed for public health decision-making settings: a scoping review | Zhao, N.; Koch-Weser, S.; Lischko, A.; Chung, M. | 2020 | International Journal of Public Health | Exclusion reason: Wrong outcomes; |
|  | Research, evidence and policymaking: the perspectives of policy actors on improving uptake of evidence in health policy development and implementation in Uganda | | 2012 | BMC Public Health | Exclusion reason: Wrong population; |
|  | Community Outreach and Education Program | | 2003 | Environmental Health Perspectives | Exclusion reason: Wrong study design; |
|  | Substance Abuse Education Web Site for Primary CARE | | 2002 |  | Exclusion reason: Wrong study design; |
