# Supplementary material for: Dissemination of public health research to prevent non-communicable diseases: a scoping review
Source: BMC Public Health. 2023 Apr 24;23:757. doi: 10.1186/s12889-023-15622-x (PMC10123991; doi:10.1186/s12889-023-15622-x)
Supplement: Supplementary file 3 — Supplementary Material 3 [file 12889_2023_15622_MOESM3_ESM.docx]

**Dissemination of public health research to prevent non-communicable diseases: A scoping review**

Supplementary Material 3

List of Included Papers by Study Design

Qualitative/Mixed Methods

1. Andruszkiewicz N, Ogunniyi C, Carfagnini C, Branston A, Hirji MM. Utilizing public health core competencies to share data effectively with community organizations to promote health equity. Canadian Journal of Public Health. 2019;110(3):303-13.
2. Armstrong R, Waters E, Crockett B, Keleher H. The nature of evidence resources and knowledge translation for health promotion practitioners. Health Promot Int. 2007;22(3):254-60.
3. Ballew P, Brownson RC, Haire-Joshu D, Heath GW, Kreuter MW. Dissemination of effective physical activity interventions: are we applying the evidence? Health Education Research. 2010;25(2):185-98.
4. Bickford JJ, Kothari AR. Research and knowledge in Ontario tobacco control networks. Can J Public Health. 2008;99(4):297-300.
5. Boydell KM, Dew A, Hodgins M, Bundy A, Gallego G, Iljadica A, et al. Deliberative Dialogues Between Policy Makers and Researchers in Canada and Australia. Journal of Disability Policy Studies. 2017;28(1):13-22.
6. Boydell KM, Stasiulis E, Barwick M, Greenberg N, Pong R. Challenges of Knowledge Translation in Rural Communities: The Case of Rural Children'S Mental Health. Canadian Journal of Community Mental Health. 2008;27(1):49-63.
7. Brown KM, Elliott SJ, Robertson-Wilson J, Vine MM, Leatherdale ST. "Now What?" Perceived Factors Influencing Knowledge Exchange in School Health Research. Health Promot Pract. 2018;19(4):590-600.
8. Budd EL, deRuyter AJ, Wang Z, Sung-Chan P, Ying X, Furtado KS, et al. A qualitative exploration of contextual factors that influence dissemination and implementation of evidence-based chronic disease prevention across four countries. BMC Health Services Research. 2018;18(1):233.
9. Busert LK, Mütsch M, Kien C, Flatz A, Griebler U, Wildner M, et al. Facilitating evidence uptake: development and user testing of a systematic review summary format to inform public health decision-making in German-speaking countries. Health Research Policy and Systems. 2018;16(1):59.
10. Campbell DM, Redman S, Jorm L, Cooke M, Zwi AB, Rychetnik L. Increasing the use of evidence in health policy: practice and views of policy makers and researchers. Australia and New Zealand Health Policy. 2009;6(1):21.
11. Chapman S, Haynes A, Derrick G, Sturk H, Hall WD, St George A. Reaching "an audience that you would never dream of speaking to": influential public health researchers' views on the role of news media in influencing policy and public understanding. J Health Commun. 2014;19(2):260-73.
12. Colley RC, Brownrigg M, Tremblay MS. A Model of Knowledge Translation in Health: The Active Healthy Kids Canada Report Card on Physical Activity for Children and Youth. Health Promotion Practice. 2012;13(3):320-30.
13. Cueva M, Dignan M, Lanier A, Kuhnley R. Qualitative Evaluation of a Colorectal Cancer Education CD-ROM for Community Health Aides/Practitioners in Alaska. Journal of Cancer Education. 2014;29(4):613-8.
14. Dagenais C, Laurendeau M-C, Briand-Lamarche M. Knowledge brokering in public health: A critical analysis of the results of a qualitative evaluation. Evaluation and Program Planning. 2015;53:10-7.
15. Dobbins M, DeCorby K, Robeson P, Husson H, Tirilis D, Greco L. A knowledge management tool for public health: health-evidence.ca. BMC Public Health. 2010;10(1):496.
16. Dobbins M, DeCorby K, Twiddy T. A knowledge transfer strategy for public health decision makers. Worldviews Evid Based Nurs. 2004;1(2):120-8.
17. Dobbins M, Greco L, Yost J, Traynor R, Decorby-Watson K, Yousefi-Nooraie R. A description of a tailored knowledge translation intervention delivered by knowledge brokers within public health departments in Canada. Health Research Policy and Systems. 2019;17(1):63.
18. Dobbins M, Jack S, Thomas H, Kothari A. Public health decision-makers' informational needs and preferences for receiving research evidence. Worldviews Evid Based Nurs. 2007;4(3):156-63.
19. Dodson EA, Baker EA, Brownson RC. Use of Evidence-Based Interventions in State Health Departments: A Qualitative Assessment of Barriers and Solutions. Journal of Public Health Management and Practice. 2010;16(6).
20. Dodson EA, Geary NA, Brownson RC. State legislators’ sources and use of information: bridging the gap between research and policy. Health Education Research. 2015;30(6):840-8.
21. Downs SM, Farmer A, Quintanilha M, Berry TR, Mager DR, Willows ND, et al. From paper to practice: barriers to adopting nutrition guidelines in schools. J Nutr Educ Behav. 2012;44(2):114-22.
22. El-Jardali F, Ataya N, Jamal D, Jaafar M. A multi-faceted approach to promote knowledge translation platforms in eastern Mediterranean countries: climate for evidence-informed policy. Health Research Policy and Systems. 2012;10(1):15.
23. Evenson KR, Satinsky SB, Valko C, Gustat J, Healy I, Litt JS, et al. In-depth interviews with state public health practitioners on the United States National Physical Activity Plan. International Journal of Behavioral Nutrition and Physical Activity. 2013;10(1):72.
24. Faulkner G, White L, Riazi N, Latimer-Cheung AE, Tremblay MS. Canadian 24-Hour Movement Guidelines for Children and Youth: Exploring the perceptions of stakeholders regarding their acceptability, barriers to uptake, and dissemination. Applied Physiology, Nutrition, and Metabolism. 2016;41(6 (Suppl. 3)):S303-S10.
25. Fernandez MA, Desroches S, Turcotte M, Marquis M, Dufour J, Provencher V. Factors influencing the adoption of a healthy eating campaign by federal cross-sector partners: a qualitative study. BMC Public Health. 2016;16(1):904.
26. Hamel N, Schrecker T. Unpacking capacity to utilize research: A tale of the Burkina Faso public health association. Social Science & Medicine. 2011;72(1):31-8.
27. Hanneke R, Link JM. The complex nature of research dissemination practices among public health faculty researchers. J Med Libr Assoc. 2019;107(3):341-51.5.
28. Haynes E, Holness DL, Tenkate T, Strahlendorf P, Kramer DM. With a little help from our friends: Collaborative research partnerships in three workplace-based occupational disease research projects. Work. 2019;62:261-78.
29. Hennessy M, Byrne M, Laws R, Mc Sharry J, O’Malley G, Heary C. Childhood obesity prevention: priority areas for future research and barriers and facilitators to knowledge translation, coproduced using the nominal group technique. Translational Behavioral Medicine. 2019;9(4):759-67.
30. Higgins JW, Strange K, Scarr J, Pennock M, Barr V, Yew A, et al. “It’s a Feel. That’s What a Lot of Our Evidence Would Consist of ”: Public Health Practitioners’ Perspectives on Evidence. Evaluation & the Health Professions. 2011;34(3):278-96.
31. Kneale D, Rojas-García A, Thomas J. Obstacles and opportunities to using research evidence in local public health decision-making in England. Health Research Policy and Systems. 2019;17(1):61.
32. Koorts H, Naylor P-J, Laws R, Love P, Maple J-L, van Nassau F. What hinders and helps academics to conduct Dissemination and Implementation (D&I) research in the field of nutrition and physical activity? An international perspective. International Journal of Behavioral Nutrition and Physical Activity. 2020;17(1):7.
33. Kothari A, Birch S, Charles C. "Interaction" and research utilisation in health policies and programs: does it work? Health Policy. 2005;71(1):117-25.
34. Macniven R, Olsen B, Shilton T, Bauman A. The initial development and process evaluation of AusPAnet: the Australian Physical Activity Network. Health Promot J Austr. 2008;19(1):36-9.
35. Malama A, Zulu JM, Nzala S, Kombe MM, Silumbwe A. Health research knowledge translation into policy in Zambia: policy-maker and researcher perspectives. Health Research Policy and Systems. 2021;19(1):42.
36. Mijumbi-Deve R, Rosenbaum SE, Oxman AD, Lavis JN, Sewankambo NK. Policymaker experiences with rapid response briefs to address health-system and technology questions in Uganda. Health Research Policy and Systems. 2017;15(1):37.
37. Milat AJ, Laws R, King L, Newson R, Rychetnik L, Rissel C, et al. Policy and practice impacts of applied research: a case study analysis of the New South Wales Health Promotion Demonstration Research Grants Scheme 2000–2006. Health Research Policy and Systems. 2013;11(1):5.
38. Mitton C, Adair CE, McKenzie E, Patten S, Waye-Perry B, Smith N. Designing a knowledge transfer and exchange strategy for the Alberta Depression Initiative: contributions of qualitative research with key stakeholders. International Journal of Mental Health Systems. 2009;3(1):11.
39. Murray A, Kelly P, Morton S, Glover D, Duncan J, Hawkes R, et al. Maximising and evaluating the uptake, use and impact of golf and health studies. British Journal of Sports Medicine. 2020;54(20):1217.
40. Nikolopoulos H, Farmer A, Berry TR, McCargar LJ, Mager DR. Perceptions of the characteristics of the Alberta Nutrition Guidelines for Children and Youth by child care providers may influence early adoption of nutrition guidelines in child care centres. Matern Child Nutr. 2015;11(2):271-82.
41. Oliver KA, de Vocht F. Defining 'evidence' in public health: a survey of policymakers' uses and preferences. Eur J Public Health. 2017;27(suppl_2):112-7.
42. Ponsford R, Ford J, Korjonen H, Hughes E, Keswani A, Pliakas T, et al. Competing for space in an already crowded market: a mixed methods study of why an online community of practice (CoP) for alcohol harm reduction failed to generate interest amongst the group of public health professionals at which it was aimed. Implementation Science. 2017;12(1):91.
43. Radisic S, Newbold KB. Factors influencing health care and service providers’ and their respective “at risk” populations’ adoption of the Air Quality Health Index (AQHI): a qualitative study. BMC Health Services Research. 2016;16(1):107.
44. Reid G, Connolly J, Halliday W, Love A-M, Higgins M, MacGregor A. Minding the gap: the barriers and facilitators of getting evidence into policy when using a knowledge-brokering approach. Evidence & Policy: A Journal of Research, Debate and Practice. 2017;13(1):29-38.
45. Riazi N, Ramanathan S, O’Neill M, Tremblay MS, Faulkner G. Canadian 24-hour movement guidelines for the early years (0–4 years): exploring the perceptions of stakeholders and end users regarding their acceptability, barriers to uptake, and dissemination. BMC Public Health. 2017;17(5):841.
46. Ritchie D, Mallafré-Larrosa M, Ferro G, Schüz J, Espina C. Evaluation of the impact of the European Code against Cancer on awareness and attitudes towards cancer prevention at the population and health promoters’ levels. Cancer Epidemiology. 2021;71:101898.
47. Shooshtari S. Factors that Facilitate and Impede Effective Knowledge Translation in Population Health Promotion: Results from a Consultation Workshop in Iran. Health Promot Perspect. 2012;2(2):126-35.
48. Strayer TE, 3rd, Kennedy LE, Balis LE, Ramalingam NS, Wilson ML, Harden SM. Cooperative Extension Gets Moving, but How? Exploration of Extension Health Educators' Sources and Channels for Information-Seeking Practices. Am J Health Promot. 2020;34(2):198-205.
49. Tremblay MS, Barnes JD, Bonne JC. Impact of the Active Healthy Kids Canada Report Card: A 10-Year Analysis. Journal of Physical Activity and Health. 2014;11(s1):S3-S20.
50. van der Graaf P, Forrest LF, Adams J, Shucksmith J, White M. How do public health professionals view and engage with research? A qualitative interview study and stakeholder workshop engaging public health professionals and researchers. BMC Public Health. 2017;17(1):892.
51. Waqa G, Mavoa H, Snowdon W, Moodie M, Nadakuitavuki R, Mc Cabe M, et al. Participants’ perceptions of a knowledge-brokering strategy to facilitate evidence-informed policy-making in Fiji. BMC Public Health. 2013;13(1):725.
52. Warren-Findlow J, Price AE, Hochhalter AK, Laditka JN. Primary care providers' sources and preferences for cognitive health information in the United States. Health Promotion International. 2010;25(4):464-73.

Quantitative

1. Alanen SI, Johannala-Kemppainen R, Ijäs JJ, Kaila M, Klockars M, Mäkelä M, et al. Evaluation of current care effectiveness: A survey of hypertension guideline implementation in Finnish health centres. Scandinavian Journal of Primary Health Care. 2007;25(4):232-6.
2. Ayah R, Jessani N, Mafuta EM. Institutional capacity for health systems research in East and Central African schools of public health: knowledge translation and effective communication. Health Research Policy and Systems. 2014;12(1):20.
3. Bellew B, Bauman A, Brown W. Evidence-based policy and practice of physical activity in Australia: Awareness and attitudes of attendees at a national physical activity conference (the PAPPA study). Health Promotion Journal of Australia. 2010;21(3):222-8.
4. Bize R, Plotnikoff RC, Scott SD, Karunamuni N, Rodgers W. Adoption of the Healthy Heart Kit by Alberta family physicians. Can J Public Health. 2009;100(2):140-4.
5. Brownson RC, Ballew P, Dieffenderfer B, Haire-Joshu D, Heath GW, Kreuter MW, et al. Evidence-based interventions to promote physical activity: what contributes to dissemination by state health departments. Am J Prev Med. 2007;33(1 Suppl):S66-73; quiz S4-8.
6. Cueva K, Cueva M, Dignan M, Landis K. Print Material in Cancer Prevention: an Evaluation of Three Booklets Designed with and for Alaska’s Community Health Workers. Journal of Cancer Education. 2016;31(2):279-84.
7. Dobbins M, Traynor RL, Workentine S, Yousefi-Nooraie R, Yost J. Impact of an organization-wide knowledge translation strategy to support evidence-informed public health decision making. BMC Public Health. 2018;18(1):1412.
8. Dunn AL, Buller DB, Dearing JW, Cutter G, Guerra M, Wilcox S, et al. Adopting an Evidence-Based Lifestyle Physical Activity Program: Dissemination Study Design and Methods. Transl Behav Med. 2012;2(2):199-208.
9. Dyson MP, Newton AS, Shave K, Featherstone RM, Thomson D, Wingert A, et al. Social Media for the Dissemination of Cochrane Child Health Evidence: Evaluation Study. Journal of medical Internet research. 2017;19(9):e308.
10. Eakin EG, Brown WJ, Marshall AL, Mummery K, Larsen E. Physical activity promotion in primary care: bridging the gap between research and practice. Am J Prev Med. 2004;27(4):297-303.
11. Evenson KR, Satinsky SB, Valko C, Gustat J, Healy I, Litt JS, et al. In-depth interviews with state public health practitioners on the United States National Physical Activity Plan. International Journal of Behavioral Nutrition and Physical Activity. 2013;10(1):72.
12. Ferdinands AR, Olstad DL, Milford KM, Maximova K, Nykiforuk CIJ, Raine KD. A Nutrition Report Card on food environments for children and youth: 5 years of experience from Canada. Public Health Nutrition. 2020;23(12):2088-99.
13. Fernández ME, DeBor M, Candreia M, Flores B. Dissemination of a Breast and Cervical Cancer Early Detection Program Through a Network of Community-Based Organizations. Health Promotion Practice. 2010;11(5):654-64.
14. Hanusaik NA. Organizational capacity and dissemination practices for chronic disease prevention in the Canadian public health system. 2008.
15. Hoelscher DM, Kelder SH, Murray N, Cribb PW, Conroy J, Parcel GS. Dissemination and adoption of the Child and Adolescent Trial for Cardiovascular Health (CATCH): a case study in Texas. J Public Health Manag Pract. 2001;7(2):90-100.
16. Jake-Schoffman DE, Wilcox S, Kaczynski AT, Turner-McGrievy G, Friedman DB, West DS. E-Media Use and Preferences for Physical Activity and Public Health Information: Results of a Web-Based Survey. Journal of Public Health Management and Practice. 2018;24(4).
17. Jessani NS, Siddiqi SM, Babcock C, Davey-Rothwell M, Ho S, Holtgrave DR. Factors affecting engagement between academic faculty and decision-makers: learnings and priorities for a school of public health. Health Research Policy and Systems. 2018;16(1):65.
18. Kelder SH, Mantey DS, Van Dusen D, Vaughn T, Bianco M, Springer AE. Dissemination of CATCH My Breath, a middle school E-Cigarette prevention program. Addictive Behaviors. 2021;113:106698.
19. Kirshbaum M, Beaver K, Luker K. Perspectives of breast care nurses on research dissemination and utilisation. Clinical Effectiveness in Nursing. 2004;8(1):47-58.
20. Kremer P, Mavoa H, Waqa G, Moodie M, McCabe M, Swinburn B. Knowledge-exchange in the Pacific: outcomes of the TROPIC (translational research for obesity prevention in communities) project. BMC Public Health. 2017;17(1):362.
21. Leeman J, Myers AE, Ribisl KM, Ammerman AS. Disseminating Policy and Environmental Change Interventions: Insights from Obesity Prevention and Tobacco Control. International Journal of Behavioral Medicine. 2015;22(3):301-11.
22. Leitlein L, Smit ES, de Vries H, Hoving C. Factors influencing Dutch practice nurses’ intention to adopt a new smoking cessation intervention. Journal of Advanced Nursing. 2012;68(10):2185-94.
23. Little MA, Pokhrel P, Sussman S, Rohrbach LA. The Process of Adoption of Evidence-based Tobacco Use Prevention Programs in California Schools. Prevention Science. 2015;16(1):80-9.
24. Loiselle CG, Semenic S, Côté B. Sharing empirical knowledge to improve breastfeeding promotion and support: description of a research dissemination project. Worldviews on Evidence‐Based Nursing. 2005;2(1):25-32.
25. Mathew M, Goldstein AO, Kramer KD, Ripley-Moffitt C, Mage C. Evaluation of a Direct Mailing Campaign to Increase Physician Awareness and Utilization of a Quitline Fax Referral Service. Journal of Health Communication. 2010;15(8):840-5.
26. Mattran K, Harris C, Jernigan J, Fulton J. Evaluating the awareness, access, and use of the State Indicator Report on Physical Activity, 2010. J Phys Act Health. 2013;10(6):863-70.
27. McBride NT, Farringdon FH, Kennedy CA. Research to practice--formal dissemination of the School Health and Alcohol Harm Reduction Project (SHAHRP) in Australia. Drug Alcohol Rev. 2007;26(6):665-72.
28. McDermott RJ, Berends V, Brown KRM, Agron P, Black KM, Barnes SP. Impact of the California Project LEAN School Board Member Social Marketing Campaign. Social Marketing Quarterly. 2005;11(2):18-40.
29. McVay AB, Stamatakis KA, Jacobs JA, Tabak RG, Brownson RC. The role of researchers in disseminating evidence to public health practice settings: a cross-sectional study. Health Research Policy and Systems. 2016;14(1):42.
30. Miro A, Kishchuk NA, Perrotta K, Swinkels HM. Healthy Canada by Design CLASP: Lessons learned from the first phase of an intersectoral, cross-provincial, built environment initiative. Can J Public Health. 2014;106(1 Suppl 1):eS50-63.
31. Monnard K, Benjamins MR, Hirschtick JL, Castro M, Roesch PT. Co-Creation of Knowledge: A Community-Based Approach to Multilevel Dissemination of Health Information. Health Promotion Practice. 2019;22(2):215-23.
32. Morshed AB, Dodson EA, Tabak RG, Brownson RC. Comparison of Research Framing Preferences and Information Use of State Legislators and Advocates Involved in Cancer Control, United States, 2012-2013. Prev Chronic Dis. 2017;14:E10.
33. Nam CS, Ross A, Ruggiero C, Ferguson M, Mui Y, Lee BY, et al. Process Evaluation and Lessons Learned From Engaging Local Policymakers in the B’More Healthy Communities for Kids Trial. Health Education & Behavior. 2018;46(1):15-23.
34. Nigg C, Geller K, Adams P, Hamada M, Hwang P, Chung R. Successful dissemination of Fun 5 — a physical activity and nutrition program for children. Translational Behavioral Medicine. 2012;2(3):276-85.
35. Scott SD, Plotnikoff RC, Karunamuni N, Bize R, Rodgers W. Factors influencing the adoption of an innovation: An examination of the uptake of the Canadian Heart Health Kit (HHK). Implementation Science. 2008;3(1):41.
36. Tabak RG, Reis RS, Wilson P, Brownson RC. Dissemination of Health-Related Research among Scientists in Three Countries: Access to Resources and Current Practices. BioMed Research International. 2015;2015:179156.
37. Tabak RG, Stamatakis KA, Jacobs JA, Brownson RC. What predicts dissemination efforts among public health researchers in the United States? Public Health Rep. 2014;129(4):361-8.
38. Waldorff FB, Steenstrup AP, Nielsen B, Rubak J, Bro F. Diffusion of an e-learning programme among Danish General Practitioners: A nation-wide prospective survey. BMC Family Practice. 2008;9(1):24.
39. Williams JR, Caceda-Castro LE, Dusablon T, Stipa M. Design, development, and evaluation of printed educational materials for evidence-based practice dissemination. JBI Evidence Implementation. 2016;14(2).
40. Worton SK, Nelson G, Loomis C, Mark Pancer S, Hayward K, Peters RD. Advancing Early Childhood Development and Prevention Programs: A Pan-Canadian Knowledge Transfer Initiative for Better Beginnings, Better Futures. Australian and New Zealand Journal of Family Therapy. 2018;39(3):347-63.

Experimental

1. Athey VL, Suckling RJ, Tod AM, Walters SJ, Rogers TK. Early diagnosis of lung cancer: evaluation of a community-based social marketing intervention. Thorax. 2012;67(5):412.
2. Benjamin SE, Tate DF, Bangdiwala SI, Neelon BH, Ammerman AS, Dodds JM, et al. Preparing Child Care Health Consultants to Address Childhood Overweight: A Randomized Controlled Trial Comparing Web to In-Person Training. Maternal and Child Health Journal. 2008;12(5):662-9.
3. Bosma M, Cassidy KL, Le Clair JK, Helsdingen S, Devichand P. A knowledge transfer study of the utility of the nova scotia seniors' mental health network in implementing seniors' mental health national guidelines. Can Geriatr J. 2011;14(1):12-6.
4. Brownson RC, Dodson EA, Stamatakis KA, Casey CM, Elliott MB, Luke DA, et al. Communicating evidence-based information on cancer prevention to state-level policy makers. J Natl Cancer Inst. 2011;103(4):306-16.
5. Buller DB, Buller MK, Kane I. Web-based strategies to disseminate a sun safety curriculum to public elementary schools and state-licensed child-care facilities. Health Psychol. 2005;24(5):470-6.
6. Buller DB, Walkosz BJ, Andersen PA, Scott MD, Dignan MB, Cutter GR, et al. Sustainability of the Dissemination of an Occupational Sun Protection Program in a Randomized Trial. Health Education & Behavior. 2011;39(4):498-502.
7. Di Noia J, Schwinn TM, Dastur ZA, Schinke SP. The relative efficacy of pamphlets, CD-ROM, and the Internet for disseminating adolescent drug abuse prevention programs: an exploratory study. Prev Med. 2003;37(6 Pt 1):646-53.
8. Dobbins M, Hanna SE, Ciliska D, Manske S, Cameron R, Mercer SL, et al. A randomized controlled trial evaluating the impact of knowledge translation and exchange strategies. Implementation Science. 2009;4(1):61.
9. Friedrich V, Brügger A, Bauer GF. Worksite Tobacco Prevention: A Randomized, Controlled Trial of Adoption, Dissemination Strategies, and Aggregated Health-Related Outcomes across Companies. BioMed Research International. 2015;2015:136505.
10. Funk M, Wutzke S, Kaner E, Anderson P, Pas L, McCormick R, et al. A multicountry controlled trial of strategies to promote dissemination and implementation of brief alcohol intervention in primary health care: findings of a World Health Organization collaborative study. J Stud Alcohol. 2005;66(3):379-88.
11. Ismail AI, Jedele JM, Lim S, Tellez M. A marketing campaign to promote screening for oral cancer. J Am Dent Assoc. 2012;143(9):e57-66.
12. Kirshbaum M. Translation to Practice: A Randomised, Controlled Study of an Evidence-Based Booklet for Breast-Care Nurses in the United Kingdom. Worldviews on Evidence-Based Nursing. 2008;5(2):60-74.
13. Lock CA, Kaner EF. Use of marketing to disseminate brief alcohol intervention to general practitioners: promoting health care interventions to health promoters. J Eval Clin Pract. 2000;6(4):345-57.
14. Tziraki C, Graubard BI, Manley M, Kosary C, Moler JE, Edwards BK. Effect of training on adoption of cancer prevention nutrition‐related activities by primary care practices: results of a randomized, controlled study. Journal of general internal medicine. 2000;15(3):155-62.
15. Williams JR, Williams WO, Dusablon T, Blais MP, Tregear SJ, Banks D, et al. Evaluation of a Randomized Intervention to Increase Adoption of Comparative Effectiveness Research by Community Health Organizations. The Journal of Behavioral Health Services & Research. 2014;41(3):308-23.
